# Design, Synthesis, and *In Silico* and *In Vitro* Cytotoxic Activities of Novel Isoniazid–Hydrazone Analogues Linked to Fluorinated Sulfonate Esters

**DOI:** 10.1021/acsomega.4c00652

**Published:** 2024-04-05

**Authors:** Eyüp Başaran, Gulal Tür, Senem Akkoc, Tugba Taskin-Tok

**Affiliations:** † Department of Chemistry and Chemical Processing Technologies, Vocational School of Technical Sciences, Batman University, Batman 72060, Turkey; ‡ Department of Chemistry, Graduate Education Institute, Batman University, Batman 72100, Turkey; § Faculty of Pharmacy, Department of Basic Pharmaceutical Sciences, Suleyman Demirel University, Isparta 32260, Turkey; ∥ Faculty of Engineering and Natural Sciences, Bahçeşehir University, Istanbul 34353, Turkey; ⊥ Department of Chemistry, Faculty of Arts and Sciences, Gaziantep University, Gaziantep 27310, Turkey; # Department of Bioinformatics and Computational Biology, Institute of Health Sciences, Gaziantep University, Gaziantep 27310, Turkey

## Abstract

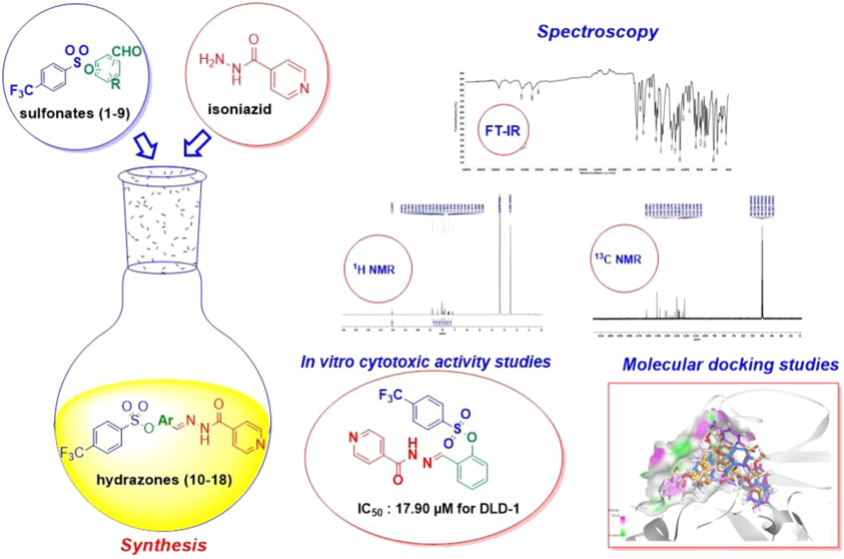

Cancer is a life-threatening disease, and significant efforts are still being made to treat it. In this study, we synthesized and characterized novel hybrid molecules (**10**–**18**) containing hydrazone and sulfonate moieties and tested their cell growth inhibitory effect on human colon cancer cells (DLD-1), human prostate cancer cells (PC3), and human embryonic kidney cells (HEK-293T) using the 3-(4,5-dimethylthiazol-2-yl)-2,5-diphenyl tetrazolium bromide (MTT) method for 72 h. In cell culture studies, all tested hybrid molecules except for **12** and **13** showed significant cytotoxic activities at a micromolar level with IC_50_ values in the range of 10.28–214.0 μM for the PC3 cell line and 13.49–144.30 μM for the DLD-1 cell line. Compounds **4** (10.28 μM) and **5** (11.22 μM) demonstrated the highest cytotoxicity against the PC3 cell line. Against the DLD-1 cell line, compounds **1** (22.53 μM), **4** (13.49 μM), **5** (19.33 μM), **6** (17.82 μM), **8** (24.71 μM), **9** (17.56 μM), and **10** (17.90 μM) in the series showed anticancer activity at lower micromolar levels compared to cisplatin (26.70 μM). Moreover, the study was handled computationally, and molecular docking studies were performed for compounds **1**, **4**, and **5** for PC3-FAK and PC3-Scr and compounds **4**, **6**, and **9** for the DLD-1-TNKS target. In this study, compound **4** was found to be the most effective and promising molecule for both targets.

## 1 Introduction

Cancer is the second cause of death in the world after cardiovascular diseases.^1−3^ It is reported that nearly 20.0 million new cancer cases were detected worldwide in 2020 and nearly 10.0 million people died due to cancer.^3,4^ Today, in cancer treatment, one or more different treatment methods such as chemotherapy, hormone therapy, surgery, immunotherapy, and radiotherapy are applied together, depending on the type and stage of cancer.^5,6^ Long-term use of current chemotherapeutic drugs used in cancer treatment can cause myelotoxicity, hepatotoxicity, urinary toxicity, cardiac toxicity, and neurotoxicity; and also, the effect of these drugs gradually decreases due to the development of drug resistance in cancer cells.^7,8^ Many researchers around the world are making great efforts to discover new effective anticancer agents that can kill cancer cells or limit their proliferation, have minimal side effects, and have high efficiency due to the increasing incidence and mortality of cancer.^9^

**Figure 1:**
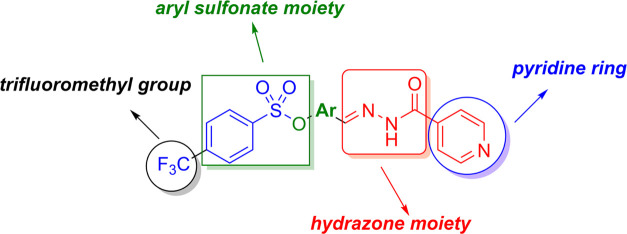
General structure of novel hybrid molecules (**10**–**18**).

The molecular hybridization method is a drug design strategy in medicinal chemistry. A new compound with more effective and more selective properties for a specific target is obtained by starting from two or more biologically active compounds. This method can lead to the development of more effective drugs with fewer side effects.^10−12^ Here, it was targeted to synthesize hybrid molecules containing four biologically active key structural motifs (a pyridine ring, trifluoromethyl group, aryl sulfonate, and hydrazone moieties) by a molecular hybridization strategy and to evaluate their antiproliferative properties (Figure 1).

**Figure 2:**
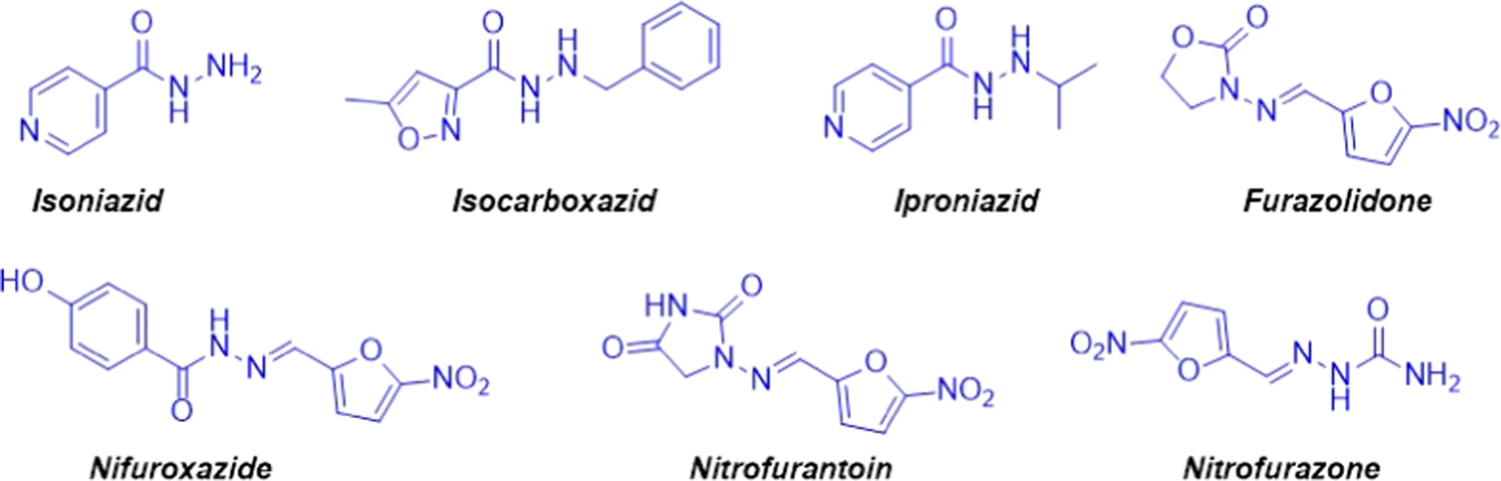
Some medications contain a hydrazide–hydrazone moiety.

Hydrazide–hydrazones are considered a fundamental framework for the development of potent and selective drugs due to their important biological activities such as antimicrobial, antioxidant, anticholinesterase, and anticarbonic anhydrase, as well as their pharmacological properties.^12−19^ In addition, since these compounds are used in the discovery of new chemotherapeutic agents, the anticancer activities of many molecules containing the hydrazide–hydrazone moiety have been investigated to date.^20−22^ Isoniazid is a drug frequently used in the treatment of tuberculosis. This drug is a first-line antimycobacterial drug commonly employed in combination with pyrazinamide, ethambutol, and rifampicin in the initial phase of treatment.^23^ Many analogues of this compound (Figure 2), such as isocarboxazid, iproniazid, furazolidone, nifuroxazide, nitrofurantoin, and nitrofurazone, are also used in the management of various disorders.^24,25^

Fluorine is an atom effective in the structure, reactivity, and functionality of fluorinated compounds that are routinely synthesized in medicinal chemistry.^26^ In recent years, molecules with fluorine atoms and a heterocyclic moiety represent an important motif in medicinal chemistry.^27,28^ The combination of these two parts gives rise to new molecules with various biological and pharmacological activities.^29−31^ In addition, aryl sulfonates are known as useful intermediates in organic synthesis. To date, many studies have reported the anticancer, anticholinesterase, and anticarbonic anhydrase activities of aryl sulfonates.^32,33^

Considering the information provided above and as a continuation of our studies on this subject, we aimed to evaluate the antiproliferative activities of newly synthesized hydrazone compounds (**10**–**18**) against human cell lines. The new hybrid molecules, namely, novel isonicotinic hydrazide–hydrazone derivatives based on trifluoromethyl-substituted aryl sulfonate esters, were facilely synthesized by the condensation reaction of sulfonate compounds (**1**–**9**) with isonicotinic hydrazide and characterized spectroscopically by elemental analysis, nuclear magnetic resonance (^1^H and ^13^C NMR), and Fourier transform infrared (FT-IR) and then tested for their cytotoxic activity in *in vitro* assays. Moreover, molecular modeling studies were performed to support the results obtained in the cytotoxic activity studies.

## 2 Results and Discussion

### 2.1 Synthesis

**Scheme 1:**
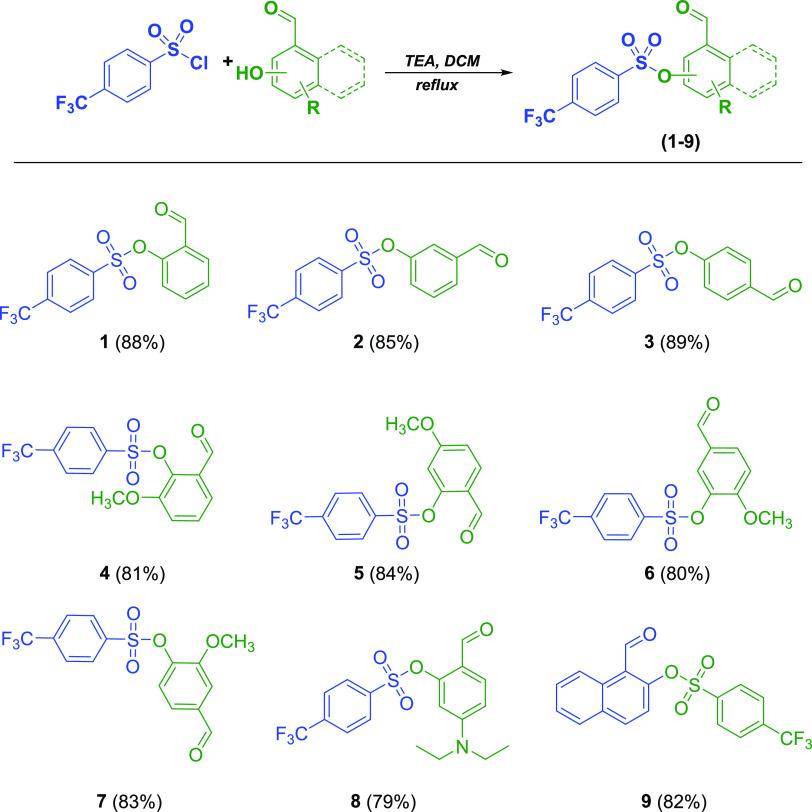
Synthesis of Aryl Sulfonate Derivatives (**1–9**)

**Scheme 2:**
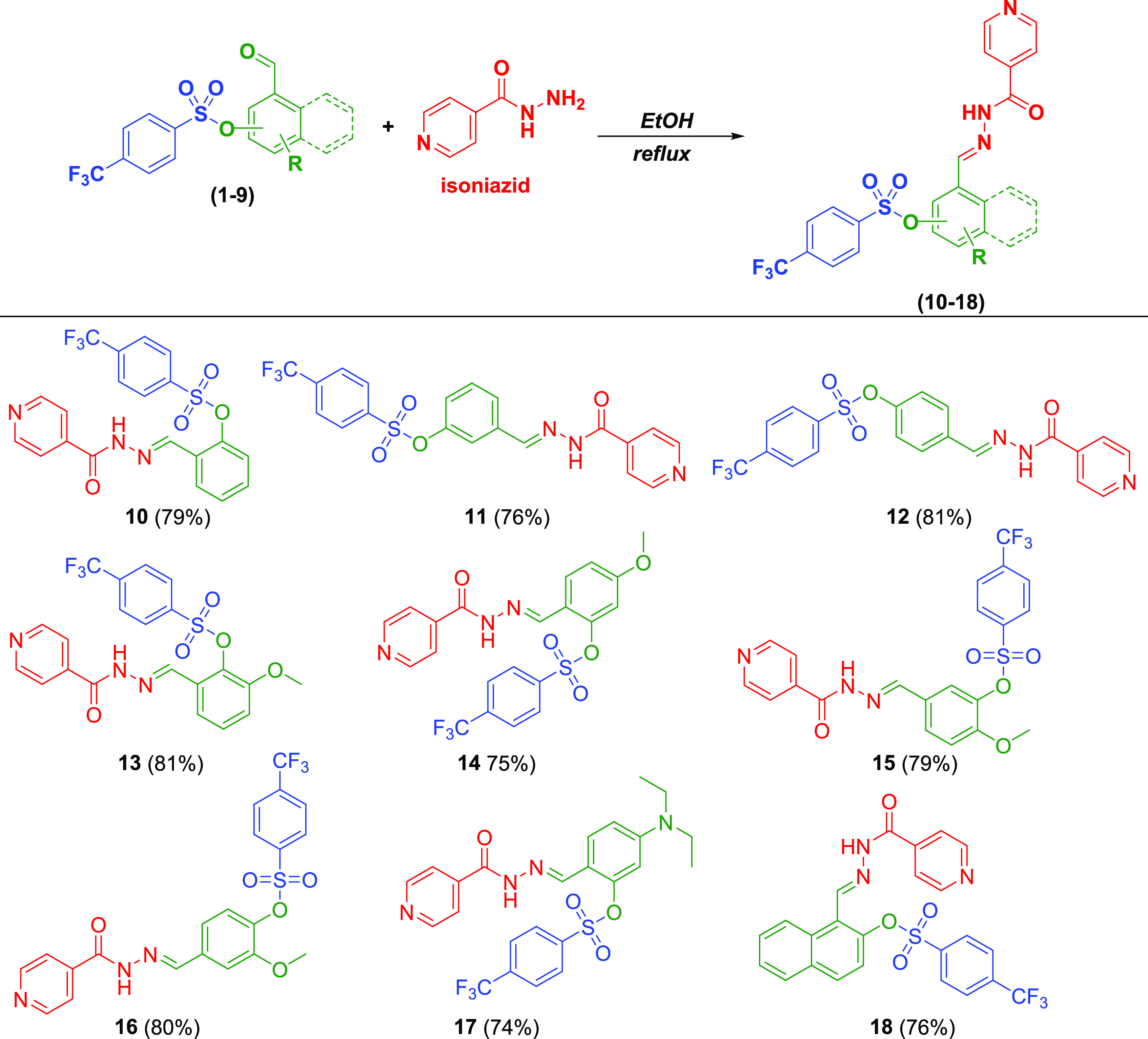
Synthesis of Novel Hydrazone Compounds (**10–18**)

Here, we designed novel isonicotinic hydrazide–hydrazone derivatives (**10**–**18**) as potential anticancer drug candidates and synthesized all tested molecules, except compound **1**,^34^ for the first time. Our approach for the preparation of novel anticancer drug candidates is based on a combination of two active moieties: isoniazid and aryl sulfonate. The synthetic strategies for the intermediate and target compounds are listed in Schemes 1 and 2. The structures of compounds were verified by elemental analysis and FT-IR, ^1^H, and ^13^C NMR. For the synthesis of sulfonate esters (**1**–**9**), nine aromatic aldehydes and 4**-**(trifluoromethyl)benzenesulfonyl chloride were reacted at reflux temperatures of dichloromethane (DCM) in the presence of triethylamine (TEA) for 4 h. The target molecules were then synthesized at reflux temperatures of ethanol for 6 h as a result of a condensation reaction between the sulfonate derivatives and isoniazid. The newly synthesized compounds were purified by recrystallization using ethanol as the solvent.

The FT-IR spectra of all synthesized compounds (**1**–**18**) were obtained using an FT-IR device with an ATR apparatus in the range of 400–4000 cm^–1^, and the stretching bands of the functional groups in their structures were determined. In the FT-IR spectra of the aryl sulfonate esters (**1**–**9**), an absorption peak representing the stretching band of the C=O group of the aldehyde functional group was observed in the range of 1668–1698 cm^–1^. Furthermore, two weak C–H stretching bands of the −CHO group were detected in the ranges of 2817–2896 cm^–1^ and 2733–2788 cm^–1^, respectively. Asymmetric and symmetric stretching bands of the SO_2_ group (O=S=O) were also observed at 1361–1381 and 1137–1178 cm^–1^, respectively. In the FT-IR spectra of the hydrazone derivative compounds (**10**–**18**), which constitute the main basis of our study, it was observed that the stretching bands belonging to the C=O, N–H, and azomethine (−N=CH−) groups were observed in the FT-IR spectra. The N–H stretching band was detected in the range 3218–3448 cm^–1^ in the hydrazone derivative compounds. The C=O stretching band was determined in the range of 1647–1676 cm^–1^, and the –C=N stretching bands, indicating the formation of the hydrazone structure, were identified in the range of 1578–1613 cm^–1^. Furthermore, the sulfonate ester moiety in the structure was found to be around 1361–1386 and 1129–1171 cm^–1^, corresponding to the asymmetric and symmetric stretching bands, respectively, which represent the presence of SO_2_ functional groups in the target compounds.

In the ^1^H NMR spectra of sulfonate ester aldehydes (**1**–**9**), the proton of the −CHO group was found to be singlet resonant at 9.69–10.49 ppm. Peaks of the protons belonging to the aromatic rings were detected between 6.28 and 9.16 ppm. The ^1^H NMR spectra of the hydrazones (**10**–**18**) obtained as a result of the treatment of the isoniazid compound with sulfonate ester aldehydes in the second step of the synthesis study, unlike the isoniazid compound, as a result of the formation of the −CH=N imine structure in the hydrazide compound at approximately 4.69 ppm according to the literature,^35^ the resonant NH_2_ protons, and the disappearance of the peak belonging to the −CHO protons are important evidence for the formation of the structures. In all hydrazone compounds, peaks belonging to the −CH=N protons resonated as singlets at 8.31–9.16 ppm. The −CONH proton resonated as a singlet at 11.85–12.29 ppm. In addition, protons belonging to the pyridine ring resonated in the range of 7.66–8.86 ppm, while protons belonging to aromatic rings resonated in the range of 5.94–8.68 ppm.

In the ^13^C NMR spectra of sulfonate ester aldehydes (**1**–**9**), the carbonyl (C=O) carbon of the −CHO group peaked at 185.08–190.71 ppm. It was determined that the carbons of the aromatic ring showed resonance in the range of 104.12–165.17 ppm. ^13^C NMR spectra of hydrazone compounds (**10**–**18**) revealed that the C=O carbon of the hydrazone structure has resonance in the range of 161.49–162.24 ppm. The carbon of the C=N group exhibited a peak in the range of 143.14–149.80 ppm. The carbons belonging to the pyridine ring, aromatic rings, and −CF_3_ groups were found to be resonant in the range of 104.10–161.78 ppm. FT-IR, ^1^H, and ^13^C NMR spectra of compounds (**1**–**18**) are given in the Supporting Information, Figures S1–S54.

### 2.2 Cytotoxic Activity Studies

Cancer treatment often damages healthy cells and tissues. Every treatment has important and diverse side effects; these depend mainly on the type and extent of treatment, are not the same for everyone, and may even vary from one treatment to another in the same person. While most cancer patients receiving chemotherapy lose hair during the treatment process, other side effects vary depending on the type of drug. Therefore, the discovery of new drugs with side effects lower than those of the chemotherapeutic agents used in treatment has become an important focus of cancer research. In the present study, the cytotoxic activities of new compounds (**1**–**18**) and cisplatin were tested in prostate and colon cancer cell lines and healthy embryonic kidney cell line for 72 h. The MTT results are given in Table 1.

**Table 1:** Experimental IC_50_ (μM) Values for Different Cancer Cell Lines (PC3 and DLD-1) as well as in a Healthy Cell Line (HEK-293T)

	IC_50_ (μM)		
compounds	PC3	DLD-1	HEK-293T
**1**	14.30 ± 1.87	22.53 ± 2.60	19.53 ± 1.57
**2**	24.48 ± 2.09	41.20 ± 3.94	59.99 ± 4.81
**3**	76.62 ± 3.55	115.20 ± 5.78	89.26 ± 0.17
**4**	10.28 ± 0.80	13.49 ± 1.64	35.28 ± 1.15
**5**	11.22 ± 0.94	19.33 ± 2.71	38.57 ± 2.81
**6**	16.03 ± 1.71	17.82 ± 0.53	90.96 ± 2.26
**7**	71.59 ± 3.46	74.05 ± 4.34	110.00 ± 5.97
**8**	28.76 ± 1.85	24.71 ± 1.90	40.80 ± 5.68
**9**	14.82 ± 1.03	17.56 ± 0.62	34.72 ± 2.21
**10**	138.90 ± 5.82	17.90 ± 0.51	52.17 ± 3.52
**11**	80.09 ± 4.59	144.30 ± 4.18	244.60 ± 5.79
**12**	>300	>300	>300
**13**	214.00 ± 6.48	78.81 ± 3.15	>300
**14**	16.48 ± 1.54	28.43 ± 2.81	26.61 ± 2.62
**15**	97.36 ± 4.85	63.75 ± 5.32	165.60 ± 6.71
**16**	120.80 ± 5.71	72.78 ± 3.13	183.30 ± 2.72
**17**	57.65 ± 1.83	110.20 ± 5.09	131.60 ± 5.46
**18**	17.05 ± 0.09	37.41 ± 4.63	15.75 ± 0.11
cisplatin	8.80 ± 0.51	26.70 ± 2.78	N.T.^a^

a N.T.: not tested.

All molecules (**1**–**18**) were screened in a human prostate cancer cell line. Table 1 shows that all but one of the 18 compounds tested inhibited the growth of PC3 cells. Compounds **1**–**9**, especially those prepared in the first step of the synthesis studies and used as reagents in the synthesis of the main targeted products, generally had a higher cytotoxic effect on prostate cancer cells with IC_50_ values of 14.30 ± 1.87, 24.48 ± 2.09, 76.62 ± 3.55, 10.28 ± 0.80, 11.22 ± 0.94, 16.03 ± 1.71, 71.59 ± 3.46, 28.76 ± 1.85, and 14.82 ± 1.03 μM, respectively. In the prepared series, molecules **4** and **5** showed the highest cytotoxic effect value, which was determined by *in vitro* MTT assays; their IC_50_ values were comparable to those of the cisplatin. The cytotoxic activities of compounds **14** and **18**, obtained from the interaction of compounds **5** and **9** with isoniazid, respectively, were close to those of the starting materials (**5**, **9**), and there were no notable decreases as in other compounds (**1**–**4**, **6**–**8**, **10**–**13**, and **15**–**17**). For example, the IC_50_ value of compound **9** was found to be 14.82 ± 1.03 μM, and the IC_50_ value of compound **18**, prepared by using compound **9** as a starting material, was found to be 17.05 ± 0.09 μM.

It was observed that compounds **1**–**9** were generally effective in the colon cancer cell line, as well as the prostate cancer cell line, and inhibited the growth of the cells. Furthermore, six aldehyde derivatives (**1**, **4**–**6**, **8**, and **9**) prepared as starting materials (**1**–**9**) were found to inhibit the growth of colon cancer cells more than cisplatin. Except for compound **12**, all of the others were found to have a cytotoxic effect on colon cancer cells. Compound **4**, which exhibited the highest cytotoxic activity in the PC3 cell line, was also determined to be the most effective compound in the DLD**–**1 cell line with an IC_50_ value of 13.49 ± 1.64 μM. Compounds **6**, **9**, and **10** showed very similar cytotoxic activity effects on colon cancer cells with IC_50_ values of 17.82 ± 0.53, 17.56 ± 0.62, and 17.90 ± 0.51 μM, respectively. Although compound **10**, prepared as the main product using compound **1** and isoniazid did not show high activity against prostate cancer, it showed an antiproliferative effect higher than that of cisplatin against the colon cancer cell line. Moreover, it showed selectivity against healthy cells with a high IC_50_ value of 52.17 ± 3.52 μM. While compounds **14** and **17** demonstrated significant cytotoxic effects against both cell lines, it was found that their selectivity on healthy cells (HEK-293T) was not high, comparable to that of compound **10**.

**Figure 3:**
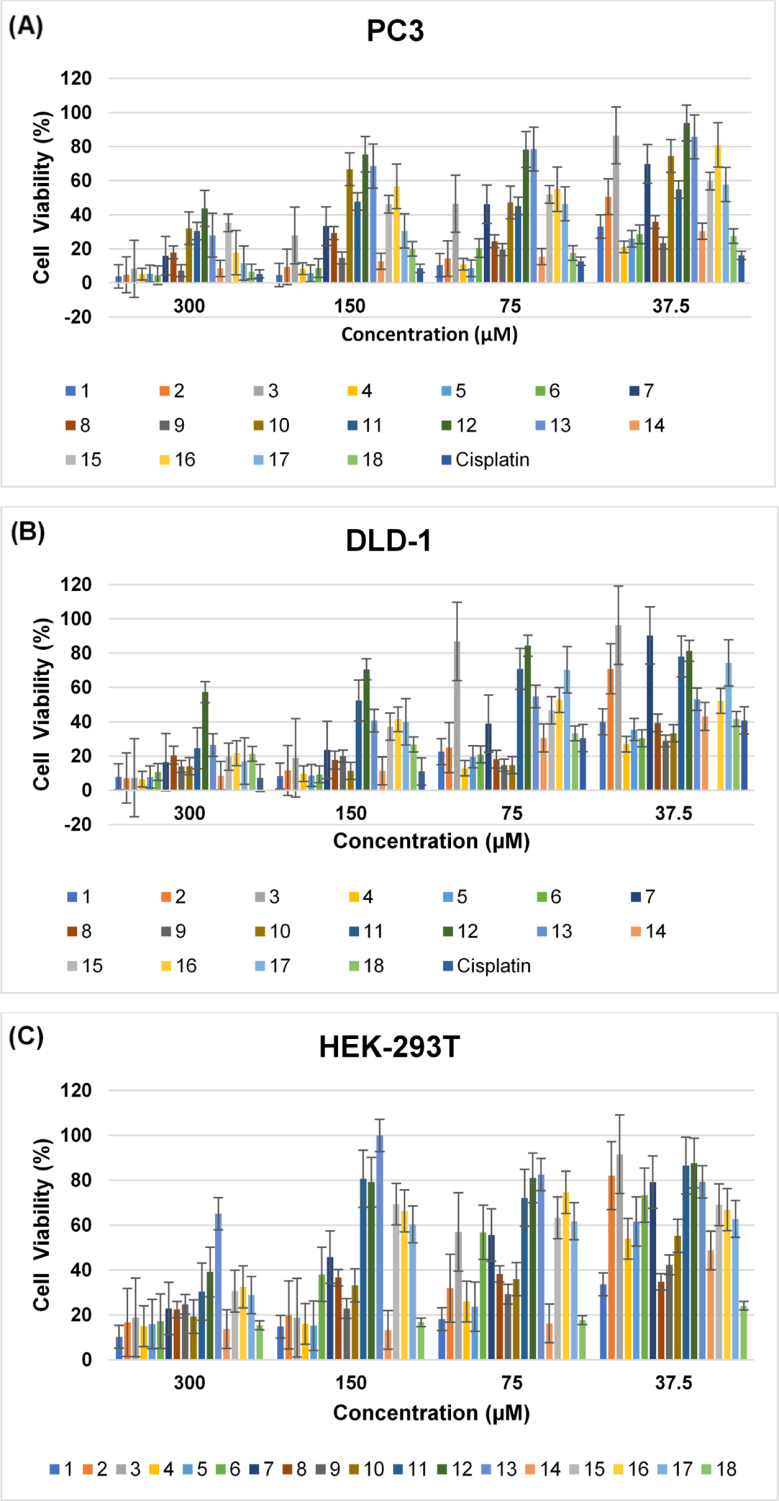
Cell viability ratio changes in the (A) prostate cancer cell line, (B) colon cancer cell line, and (C) healthy cell line depending on the concentrations of the tested compounds.

To determine whether all of the prepared substances had selectivity for healthy cells, the compounds were tested against HEK-293T cells at the same concentrations as those applied to cancer cells. Except for compound **12**, which was already seen to be inactive in the other two cell lines, compound **13** was found to be inactive in this cell line (HEK-293T) because their IC_50_ values were greater than 300 μM. Table 1 shows that other compounds (**1**–**11**, **14**–**18**) have toxic effects on healthy cells, with IC_50_ values ranging from 15.75 to 244.60 μM. Changes in the cell viability ratio depending on concentrations of the tested compounds (**1**–**18**) and cisplatin are given below (Figure 3).

### 2.3 Results of Docking Studies

In this study, geometric and energy optimizations of all synthesized compounds were carried out, and the output data are presented in Tables S1–S18. Then, based on the results of investigating their biological activities against prostate and colon cancer, compounds **1**, **4**, and **5**, which showed potential effectiveness, were first examined at the molecular level with prostate cancer cell models through molecular docking studies. The study results are listed in Table 2 by calculating the binding energy and inhibition constant values.

**Table 2:** Binding Energy and Inhibition Constant (*K*_i_) Values of Compounds **1**, **4**, and **5** for PC3 (FAK and Src), **4**, **6**, **9**, **10**, and **14** for DLD–1-TNKS, and also Cisplatin, as a Positive Control Compound for Both Target Models

PC3-FAK	binding energy (kcal/mol)	*K*_i_ (μM)
**1**	–6.63	13.69
**4**	–6.92	8.46
**5**	–6.82	9.99
cisplatin	–6.48	17.84

PC3-Src	binding energy (kcal/mol)	*K*_i_ (μM)
**1**	–7.21	5.19
**4**	–7.41	3.71
**5**	–7.34	4.2
cisplatin	–6.63	13.8

DLD-1-TNKS	binding energy (kcal/mol)	*K*_i_ (μM)
**4**	–8.86	0.319
**6**	–7.09	6.34
**9**	–8.00	1.36
**10**	–7.00	7.44
**14**	–6.97	7.75
cisplatin	–6.61	14.22

**Figure 4:**
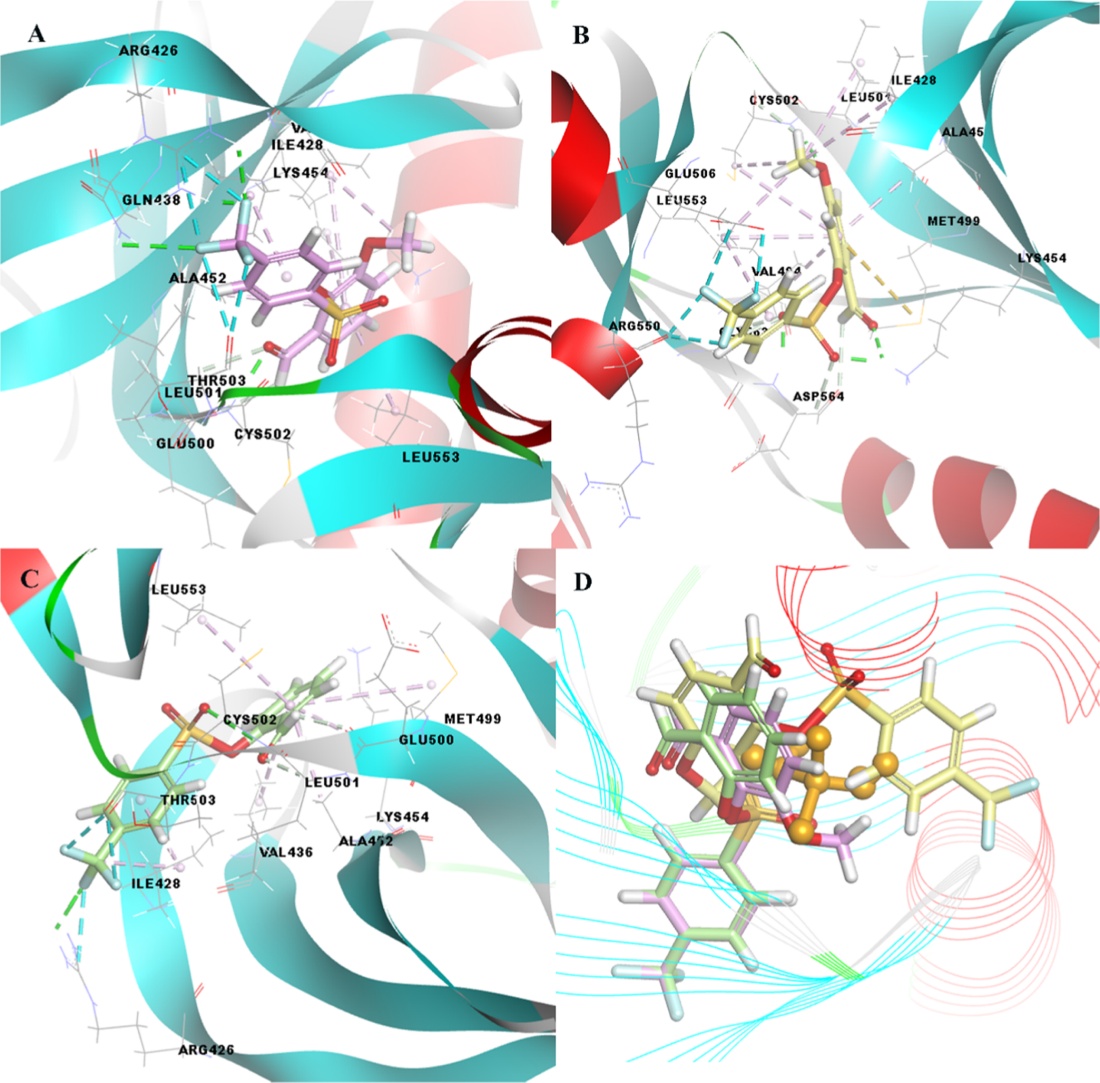
3D docking images of (A) compound **4** (pink color), (B) compound **5** (yellow color), (C) compound **1** (light green color), and (D) superimposed form of the related compounds and cisplatin (orange color, ball, and stick form) as a positive control compound, against PC3-FAK.

Three potential active compound candidates were investigated based on FAK and Src models selected as prostate cancer models, as shown in Figure 4. First, compound **4** exhibited the best binding affinity with the FAK target and showed a binding energy of −6.92 kcal/mol. The binding of the formyl and methoxy groups at the *ortho* positions of the phenyl ring of compound **4** increased the chemical reactivity of the structure. As a result, the formyl group of the compound forms hydrogen bonds with Cys502, Leu501, and Glu500 of the target protein (as given in Figure S58), whereas the methoxy group of the compound forms a hydrophobic interaction with Val436 of the relevant target. In addition, trifluoromethyl at the *para* position of benzene forms both hydrogen and halogen bonds in Gln438 and Arg426 of the related protein. The trifluoromethyl group of compound **4** interacted with Arg426 and Thr503 of the relevant target to form halogen bonds. If the second active compound is compound **5**, the position difference between the formyl and methoxy groups in the phenyl ring changes the chemical reactivity of the relevant compound compared with compound **4**. In other words, it causes the orientation of the interaction with the target enzyme to differ. This situation is illustrated in Figure 4. The last active molecule, compound **1**, showed a lower tendency to bind to the enzyme because it has less reactivity than compound **4**; that is, the methoxy group is not present in the phenyl ring. When the situation of the three active molecules is compared with that of cisplatin, which is the reference compound, the topological surface areas are considerably larger than those of the reference compound, as shown in Figure 4. Therefore, it exhibited a binding tendency of less than three potential molecules with the target structure, namely, −6.48 kcal/mol.

**Figure 5:**
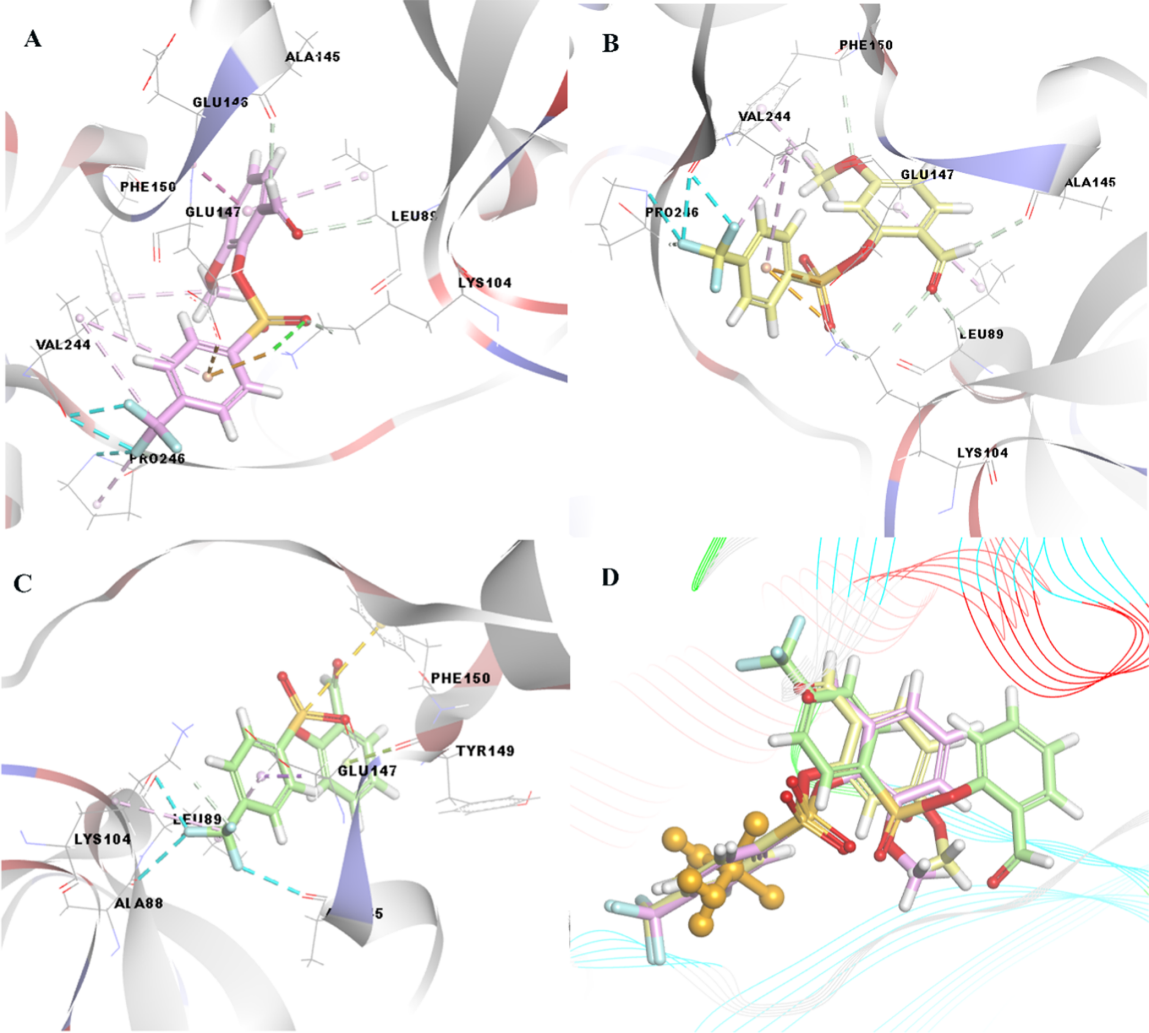
3D docking images of (A) compound **4** (pink color), (B) compound **5** (yellow color), (C) compound **1** (light green color), and (D) superimposed form of the related compounds and cisplatin (orange color, ball, and stick form) as a positive control compound, against PC3-Scr.

Furthermore, when the interactions of the same three compounds with the second target Src, considered for prostate cancer, were evaluated, compound **4** was again the most effective molecule on this target, with a binding energy value of −7.41 kcal/mol, compound **5** showed a binding energy value of −7.34 kcal/mol, while compound **1** showed a binding energy value of −7.21 kcal/mol. The interaction of the compounds with this target protein was stronger than that of the FAK target. Electrostatic interactions are included here as well as hydrogen, halogen, and hydrophobic interactions. The orientation and interactions of the three potential compounds with the target are shown in Figure 5, and detailed interaction data are presented in Table S19.

**Figure 6:**
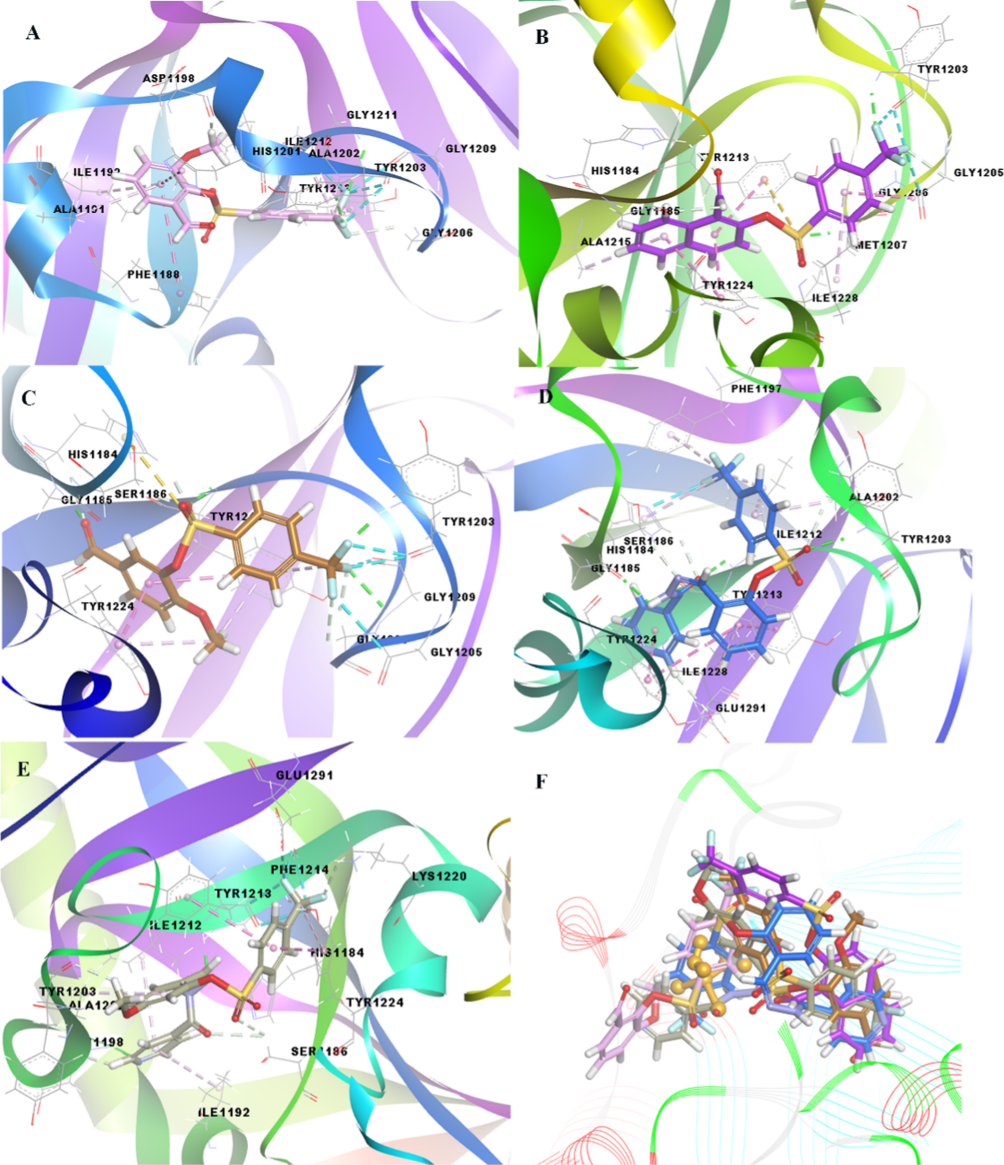
3D docking images of (A) compound **4** (pink color), (B) compound **9** (purple color), (C) compound **6** (brown color), (D) compound **10** (dark blue color), (E) compound **14** (military green color), and (F) superimposed form of the related compounds and cisplatin (orange color, ball, and stick form) as a positive control compound, against DLD-1-TNKS.

In addition to prostate cancer, the biological activity of compounds **4**, **6**, **9**, **10**, and **14**, among the synthesized compounds, on colon cancer (DLD-1-TNKS) was examined by molecular docking. As a result of the evaluations, compounds **4**, **6**, **9**, **10**, and **14**, which exhibited the best activity, exhibited binding energy values of −8.80, −8.00, 7.09, −7.00, and −6.97 kcal/mol, respectively. The structure of compound **4** formed hydrogen and halogen bonds with the formyl and methoxy groups on the phenyl ring and the trifluoromethyl group on the other benzene ring, similar to the targets in prostate cancer. In addition, it creates hydrophobic interactions with aromatic ring systems. The structure of compound **9** remained linear because of the naphthalene group in its structure. As shown in Figure 6, the interaction of compound **9** with the target differed from that of compound **4**. In addition, unlike in the other compounds, sulfur bond formation occurs with the Tyr1213 residue of the target protein. Finally, unlike compound **4**, compound **6** has a formyl group in the *meta* position instead of *ortho*, causing a change in the binding orientation of compound **6** with the target and resulting in interaction differences.

In addition, the hydrazone-derived structures of compounds **1** and **5** exhibited distant bond interactions with different residues of the target as a result of different orientations in the active region of the same target of compounds **10** and **14**, respectively, unlike the other three compounds. However, what should be noted here is that if the formly group is replaced by isonicotinoylhydrazineylidene, the binding tendency with the target protein decreases to −7.00 kcal/mol. When we examined the interaction with the target in more detail in Figure 6, the trifluoromethyl part of compound **10** formed a halogen bond with the target’s His1184 and a hydrophobic interaction with Ile1212, Phe1197, and His1184. In addition, the sulfonyl group hydrogen bonds to Gly1206 of the target, hydrogen bonds in the hydrazone part of the related compound with Gly1185 and Tyr1213, and the Tyr1224 residue exhibits hydrophobic interactions with the ring system. Although compound **10** exhibits a larger molecular weight and surface area than compounds **4**, **6**, and **9**, it interacts with the target in the opposite direction and orientation compared to compound **4** in the active region. For these reasons, the interaction of compound **10** with the target was much weaker than that of compound **4**. In the same situation, compound **14**, that is, the other hydrazone-derived molecular structure, created a binding energy of −6.97 kcal/mol as a result of the orientation difference in the active region due to the greater steric effect. The compound mentioned in Figure 6 interacts with the target, but the distant bond interactions they form cannot exhibit activity with the target, such as other low-molecular-weight compounds **4**, **6**, or **9**. Details of these findings are presented in Table S19.

## 3 Experimental Section

### 3.1 Physical Measurements, Chemicals, and Reagents

All chemicals used in this study were purchased from commercial suppliers (Merck, Sigma-Aldrich, and Thermo Fisher Scientific). ^1^H and ^13^C NMR spectra were recorded for compounds (**1**–**9**) in CDCl_3_ and compounds (**10**–**18**) in DMSO-*d*_6_ solutions on a Bruker AVANCE III 400 MHz spectrometer. Elemental analyses were performed by using a Thermo Scientific Flash 2000 elemental analyzer. Infrared spectra were recorded on an Agilent Cary 630 spectrophotometer.

### 3.2 Synthesis of Aryl Sulfonate Esters (**1**–**9**)

The aryl sulfonate ester synthesis is described in detail in our previously published study.^36^

### 3.3 Synthesis of Trifluoromethyl-Substituted Aryl Sulfonate–Hydrazone Hybrids (**10**–**18**)

Isonicotinic hydrazide (2 mmol) and aryl sulfonate (2 mmol) were dissolved in ethanol (10 mL). The reaction mixture was stirred at reflux for 6 h. The mixture was cooled to 25 °C. The product formed was aspirated by filtration, washed with diethyl ether, and then crystallized in ethanol.

### 3.4 Cytotoxic Activity Studies

The PC3 and DLD-1 cell lines were purchased from the American Type Culture Collection. The PC3, DLD-1, and HEK-293T cells were seeded in 96-well plates at a density of 5 × 10^3^ cells/well for cytotoxic activity studies.^36−38^ Cells were exposed to compounds at concentrations of 300, 150, 75, and 37.5 μM after 24 h. The 50 μL (5 mg/mL) amount of MTT stock solution was added to wells after 72 h and incubated for a further 2 h. The absorbance values were measured by using an Epoch 2 ELISA plate reader at 590 nm.

### 3.5 Molecular Docking Studies

This is a descriptive, experimental, quantitative, *in silico*, and *in vitro* study using DLD-1 and PC3 cell lines. *In silico* data indicate that ligands can interact with focal adhesion kinase, FAK (Protein Data Bank (PDB): 1MP8, https://www.rcsb.org/structure/1mp8, 1.60 Å) and human tyrosine-protein kinase C-Src (PDB: 1FMK, https://www.rcsb.org/structure/1fmk, 1.50 Å) for prostate as well as tankyrase (PDB: 5ETY, https://www.rcsb.org/structure/5ETY, 2.90 Å) for colon cancer.

In this study, we put forth the proposition to assess diverse phenomena linked to the advancement of tumors. These phenomena encompass cell migration and invasion, which exhibit notable associations with the FAK/Src signaling pathway.^39^ At the same time, the Wnt/β-catenin pathway is a widely recognized oncogenic pathway. The inhibition of its growth has been regarded as a notable obstacle, particularly in the management of individuals diagnosed with colon cancer. A transcriptional reporter assay was utilized to perform high-throughput screening in APC mutant DLD-1 cells to uncover small-molecule inhibitors of the Wnt/β-catenin pathway. This screening led to the identification of K-756, which is a selective inhibitor of the Wnt/β-catenin pathway. It was later determined that K-756 is a tyranolysis (TNKS) inhibitor. TKNS, the poly-ADP, a member of the PARP family, exhibits ribosylation activity toward Axin and facilitates Axin degradation through the proteasome pathway. Moreover, assays on enzymes belonging to the PARP family demonstrated the selectivity of K-756 as a TNKS inhibitor. K-756 was observed to hinder cellular proliferation in APC mutant colorectal cancer COLO 320DM and SW403 cells by impeding the Wnt/β-catenin signaling pathway. Furthermore, oral administration of K-756 blocked the Wnt/β-catenin pathway in mouse colon cancer xenografts, according to an *in vivo* study.^36^ Thus, new hybrid molecules (ligands) and FAK/Src, tankyrase (proteins), were subjected to a series of molecular docking experiments. The optimized geometry of **1**–**18** was computed with the help of Gaussian 09, the Revision E.01 program package^40^ at the DFT/B3LYP/Land2DZ level.^41,42^ The potential molecules (**1**, **4**, and **5** for PC3-FAK/Src; and **4**, **6**, **9**, **10**, and **14** for DLD-1) with PC3-FAK, PC3-Src, and DLD-1 protein models were carried out with Auto Dock 4.2.^43^ The spherical grid was centered at X: 37.50 Y: −4.10 Z: 25.50 for FAK; X: −19.20 Y: −5.19 Z: 25.20 for Src; and X: −36.50 Y: 25.10 Z: −25.71 for TNKS with 200 Lamarckian Genetic Algorithm (LGA) runs.

The target models [PC3 (FAK and Src) and (DLD-1-TNKS)] were uploaded to the protein database. The target model was carried out using a predocking process including polar hydrogen atoms inserted, and undesired molecules such as water were removed. Finally, CHARMm force fields^44^ were used to minimize the targets. The lowest binding energy values and 3D interactions were filtered through the generated 200 conformations. Additionally, [(2*R*,3*S*,4*R*,5*R*)-5-(6-aminopurin-9-yl)-3,4-dihydroxy-oxolan-2-yl]methyl phosphono hydrogen phosphate (ADP), (2S)-2- amino-3-(4-phosphonooxyphenyl)propanoic acid (PTR), and 3-[[1-(6,7-dimethoxyquinazolin-4-yl)piperidin-4-yl]methyl]-1,4-dihydroquinazolin-2-one (K56) contain crystallized ligands for FAK, Src, and TNKS, respectively. Each of our docking protocols using these ligands has been validated and the average RMSD values are 1.846, 1.537, and 1.392 Å for each target, respectively. Docking results were viewed and assessed using Discovery Studio (DS) 3.5^45^ based on the lowest binding energy data of the generated complexes. In the biological activity study, interactions were created based on the cisplatin determined for each model target [PC3 (FAK and Src) and DLD-1 (TNKS)], and the obtained 3D view outputs are shown in Figures S55 and S57, respectively.

## 4 Conclusions

In conclusion, we synthesized and characterized novel hybrid compounds (**10**–**18**) containing two crucial pharmacophores (hydrazone and sulfonate moieties). All compounds were evaluated for their cytotoxic activity properties in human cell lines by using the MTT method for 72 h. We determined that aryl sulfonate esters showed greater antiproliferative activity than hydrazone derivatives against cancer cell lines. Among tested molecules, compound **4** was the most potent inhibitor of the tested cancer cell lines. Compounds **4** and **5** were found to have potent antiproliferative activity closest to that of cisplatin in the PC3 cell line. On the other hand, many compounds (**1**, **4**–**6**, and **8–10**) showed better cell growth inhibitory effects than cisplatin in the DLD-1 cell line. The results of the molecular docking study were in agreement with the biological activity results. The docking study showed that compound **4** was the most effective agent against prostate and colon cancer cell lines.

## Supporting Information Available

Analytical, spectral data and spectra (FT-IR, ^1^H, and ^13^C NMR) of compounds (**1**–**18**), and detailed molecular docking studies (PDF)
