# Supplementary material for: Design, Synthesis, and In Silico and In Vitro Cytotoxic Activities of Novel Isoniazid–Hydrazone Analogues Linked to Fluorinated Sulfonate Esters
Source: ACS Omega. 2024 Apr 5;9(15):17551–62. doi: 10.1021/acsomega.4c00652 (PMC11025081; doi:10.1021/acsomega.4c00652)
Supplement: Supplementary file 1 — ao4c00652_si_001.pdf [file ao4c00652_si_001.pdf]

Supporting Information

Design, synthesis, *in silico,* and *in vitro* cytotoxic activities of novel isoniazid-hydrazone analogs linked to fluorinated sulfonate esters

Eyüp Başaran[1,*] , Gulal Tür[2], Senem Akkoc[3,4,*], Tugba Taskin-Tok[5,6]

[1]*Department of Chemistry and Chemical Processing Technologies, Vocational School of Technical Sciences, Batman University, Batman, 72060, Türkiye*

[2] *Department of Chemistry, Graduate Education Institute, Batman University, Batman, 72100, Türkiye*

[3]*Suleyman Demirel University, Faculty of Pharmacy, Department of Basic Pharmaceutical Sciences, Isparta, 32260, Türkiye*

[4]*Bahçeşehir University, Faculty of Engineering and Natural Sciences, Istanbul, 34353, Türkiye*

[5]*Department of Chemistry, Faculty of Arts and Sciences, Gaziantep University, Gaziantep, 27310, Türkiye*

[6]*Department of Bioinformatics and Computational Biology, Institute of Health Sciences, Gaziantep University, Gaziantep, 27310, Türkiye*

Contents ***Pages***

1. Analytical and Spectral Data of Synthesized Compounds…………………..**2-12**

2. FT-IR, ^{1}H- and ^{13}C- NMR spectra of the synthesized compounds………..**13-39**

3. Molecular Docking Studies……………………………………………………**40-74**

* Corresponding authors:

*Eyüp Başaran

Department of Chemistry and Chemical Processing Technologies, Vocational School of Technical Sciences, Batman University, Türkiye.

*Senem Akkoc

Suleyman Demirel University, Faculty of Pharmacy, Department of Basic Pharmaceutical Sciences, Isparta, 32260, Türkiye.

E-mail addresses: eyup.basaran@batman.edu.tr (E.Basaran), senemakkoc@sdu.edu.tr (S. Akkoc)

1. Analytical and Spectral Data of Synthesized Compounds

2-Formylphenyl 4-(trifluoromethyl)benzenesulfonate (1)

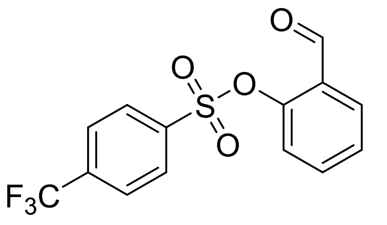

White color solid; yield 88%; m.p.: 83-84 °C; FT-IR/ATR: υ_{max} = 3101, 3064 (C-H str.)$_{aromatic}$, 2896, 2754 (C-H str.)$_{aldehyde}$, 1690 (C=O str.), 1361 (O=S=O str.)$_{antisymmetric}$, 1139 (O=S=O str.)$_{symmetric}$ cm^{-1} (Figure S1); ^1H NMR ($CDCl_3$, 400 MHz): δ = 10.10 (s, 1H, -C*H*=O), 8.06 (d, *J* = 8.0 Hz, 2H, aromatic-*H*), 7.94 (dd, *J* = 7.6, 1.6 Hz, 1H, aromatic-*H*), 7.87 (d, *J* = 8.0 Hz, 2H, aromatic-*H*), 7.68 – 7.59 (m, 1H, aromatic-*H*), 7.48 (t, *J* = 7.6 Hz, 1H, aromatic-*H*), 7.20 (d, *J* = 8.4 Hz, 1H, aromatic-*H*) ppm (Figure S2); ^{13}C NMR ($CDCl_3$, 100 MHz): δ = 187.04 (*C*HO), 150.45, 138.16, 136.30, 135.52, 129.53, 129.22, 129.16, 128.05, 126.72, 126.69, 123.39 (aromatic-*C* and *C*F_3) ppm (Figure S3); Anal. Calcd. for $C_{14}H_9F_3O_4S$: C, 50.91; H, 2.75; S, 9.71%. Found: C, 51.02; H, 2.78; S, 9.84%.

3-Formylphenyl 4-(trifluoromethyl)benzenesulfonate (2)

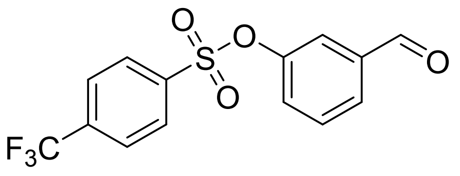

White color solid; yield 85%; m.p.: 79-80 °C; FT-IR/ATR: υ_{max} = 3109, 3075 (C-H str.)$_{aromatic}$, 2817, 2736 (C-H str.)$_{aldehyde}$, 1693 (C=O str.), 1374 (O=S=O str.)$_{antisymmetric}$, 1137 (O=S=O str.)$_{symmetric}$ cm^{-1} (Figure S4); ^1H NMR ($CDCl_3$, 400 MHz): δ = 9.98 (s, 1H, -C*H*=O), 8.03 (d, *J* = 8.2 Hz, 2H, aromatic-*H*), 7.88 – 7.83 (m, 3H, aromatic-*H*), 7.59 – 7.54 (m, 2H, aromatic-*H*), 7.34 – 7.31 (m, 1H, aromatic-*H*), ppm (Figure S5); ^{13}C NMR ($CDCl_3$, 100 MHz): δ = 190.39 (*C*HO), 149.80, 138.06, 130.77, 129.06, 128.83, 128.07, 126.63, 126.60, 126.56, 126.52, 122.66 (aromatic-*C* and *C*F_3) ppm (Figure S6); Anal. Calcd. for $C_{14}H_9F_3O_4S$: C, 50.91; H, 2.75; S, 9.71%. Found: C, 50.84; H, 2.72; S, 9.65%.

4-Formylphenyl 4-(trifluoromethyl)benzenesulfonate (3)

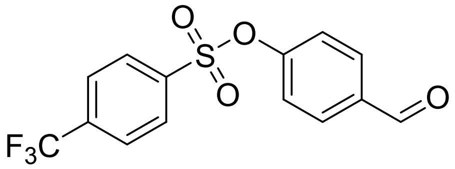

White color solid; yield 89%; m.p.: 110-111 °C; FT-IR/ATR: υ_{max} = 3111, 3064 (C-H str.)$_{aromatic}$, 2852, 2746 (C-H str.)$_{aldehyde}$, 1698 (C=O str.), 1374 (O=S=O str.)$_{antisymmetric}$, 1137 (O=S=O str.)$_{symmetric}$ cm^{-1} (Figure S7); ^{1}H NMR ($CDCl_3$, 400 MHz): δ = 10.01 (s, 1H, -C*H*=O), 8.03 (d, *J* = 8.0 Hz, 2H, aromatic-*H*), 7.90 (d, *J* = 8.4 Hz, 2H, aromatic-*H*), 7.86 (d, *J* = 8.4 Hz, 2H, aromatic-*H*), 7.23 (d, *J* = 8.4 Hz, 2H, aromatic-*H*) ppm (Figure S8); ^{13}C NMR ($CDCl_3$, 100 MHz): δ = 190.51 (*C*HO), 153.36, 138.60, 136.38, 135.17, 131.50, 129.05, 126.61, 126.57, 122.94 (aromatic-*C* and *C*F_3) ppm (Figure S9); Anal. Calcd. for $C_{14}H_9F_3O_4S$: C, 50.91; H, 2.75; S, 9.71%. Found: C, 50.95; H, 2.77; S, 9.82%.

2-Formyl-6-methoxyphenyl 4-(trifluoromethyl)benzenesulfonate (4)

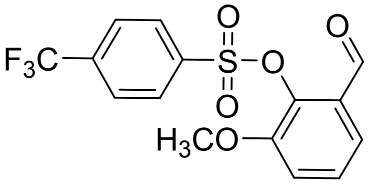

White color solid; yield 81%; m.p.: 105-106 °C; FT-IR/ATR: υ_{max} = 3089, 3018 (C-H str.)$_{aromatic}$, 2934, 2884 (C-H str.)$_{aliphatic}$, 2837, 2768 (C-H str.)$_{aldehyde}$, 1698 (C=O str.), 1375 (O=S=O str.)$_{antisymmetric}$, 1142 (O=S=O str.)$_{symmetric}$ cm^{-1} (Figure S10); ^{1}H NMR ($CDCl_3$, 400 MHz): δ = 10.28 (s, 1H, -C*H*=O), 8.10 (d, *J* = 8.4 Hz, 2H, aromatic-*H*), 7.87 (d, *J* = 8.4 Hz, 2H, aromatic-*H*), 7.57 (dd, *J* = 8.0, 1.6 Hz, 1H, aromatic-*H*), 7.39 (t, *J* = 8.0 Hz, 1H, aromatic-*H*), 7.12 (dd, *J* = 8.2, 1.4 Hz, 1H, aromatic-*H*), 3.51 (s, 3H, -OC*H*$_3$) ppm (Figure S11); ^{13}C NMR ($CDCl_3$, 100 MHz): δ = 187.74 (*C*HO), 151.93, 139.93, 139.47, 135.78, 131.23, 129.24, 128.37, 126.08, 126.05, 119.96, 117.85 (aromatic-*C* and *C*F_3), 55.68 (-O*C*H_3) ppm (Figure S12); Anal. Calcd. for $C_{15}H_{11}F_3O_5S$: C, 50.00; H, 3.08; S, 8.90%. Found: C, 50.09; H, 3.13; S, 8.84%.

2-Formyl-5-methoxyphenyl 4-(trifluoromethyl)benzenesulfonate (5)

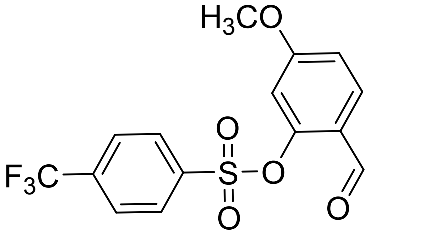

White color solid; yield 84%; m.p.: 113-115 °C; FT-IR/ATR: υ_{max} = 3107, 3046 (C-H str.)$_{aromatic}$, 2955, 2890 (C-H str.)$_{aliphatic}$, 2843, 2788 (C-H str.)$_{aldehyde}$, 1686 (C=O str.), 1387 (O=S=O str.)$_{antisymmetric}$, 1173 (O=S=O str.)$_{symmetric}$ cm^{-1} (Figure S13); ^{1}H NMR (CDCl$_3$, 400 MHz): δ = 9.88 (s, 1H, -C*H*=O), 8.07 (d, *J* = 8.2 Hz, 2H, aromatic-*H*), 7.88 – 7.85 (m, 3H, aromatic-*H*), 6.99 – 6.92 (m, 1H, aromatic-*H*), 6.74 (d, *J* = 2.4 Hz, 1H, aromatic-*H*), 3.87 (s, 3H, -OC*H*$_3$) ppm (Figure S14); ^{13}C NMR (CDCl$_3$, 100 MHz): δ = 185.85 (*C*HO), 165.17, 152.03, 138.16, 136.28, 130.99, 129.16, 126.70, 126.66, 122.36, 113.94, 108.64 (aromatic-*C* and *C*F$_3$), 56.05 (-O*C*H$_3$) ppm (Figure S15); Anal. Calcd. for $C_{15}H_{11}F_3O_5S$: C, 50.00; H, 3.08; S, 8.90%. Found: C, 49.92; H, 3.04; S, 8.97%.

5-Formyl-2-methoxyphenyl 4-(trifluoromethyl)benzenesulfonate (6)

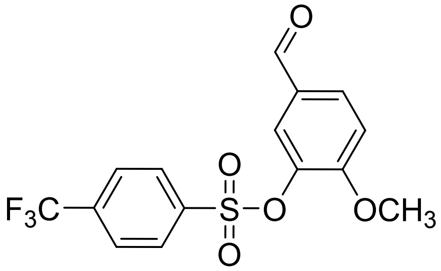

White color solid; yield 80%; m.p.: 116-117 °C; FT-IR/ATR: υ_{max} = 3107, 3049 (C-H str.)$_{aromatic}$, 3019, 2958 (C-H str.)$_{aliphatic}$, 2823, 2733 (C-H str.)$_{aldehyde}$, 1690 (C=O str.), 1370 (O=S=O str.)$_{antisymmetric}$, 1171 (O=S=O str.)$_{symmetric}$ cm^{-1} (Figure S16); ^{1}H NMR (CDCl$_3$, 400 MHz): δ = 9.89 (s, 1H, -C*H*=O), 8.06 (d, *J* = 8.4 Hz, 2H, aromatic-*H*), 7.85 – 7.82 (m, 3H, aromatic-*H*), 7.75 (d, *J* = 2.0 Hz, 1H, aromatic-*H*), 7.01 (d, *J* = 8.4 Hz, 1H, aromatic-*H*), 3.65 (s, 3H, -OC*H*$_3$) ppm (Figure S17); ^{13}C NMR (CDCl$_3$, 100 MHz): δ = 189.51 (*C*HO), 156.47, 139.55, 138.40, 130.89, 129.95, 129.13, 126.08, 126.05, 126.01, 125.02, 112.57 (aromatic-*C*

and CF_3), 56.95 (-OCH_3) ppm (Figure S18); Anal. Calcd. for $C_{15}H_{11}F_3O_5S$: C, 50.00; H, 3.08; S, 8.90%. Found: C, 50.11; H, 2.99; S, 8.86%.

4-Formyl-2-methoxyphenyl 4-(trifluoromethyl)benzenesulfonate (7)

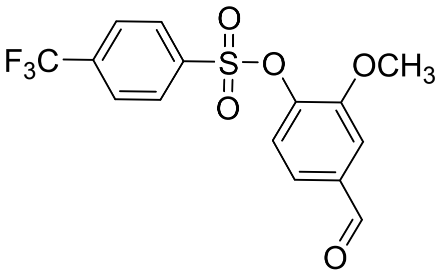

White color solid; yield 83%; m.p.: 112-113 °C; FT-IR/ATR: υ_{max} = 3107, 3069 (C-H str.)$_{aromatic}$, 2989, 2966 (C-H str.)$_{aliphatic}$, 2835, 2741 (C-H str.)$_{aldehyde}$, 1696 (C=O str.), 1373 (O=S=O str.)$_{antisymmetric}$, 1178 (O=S=O str.)$_{symmetric}$ cm^{-1} (Figure S19); ^{1}H NMR ($CDCl_3$, 400 MHz): δ = 9.97 (s, 1H, -C*H*=O), 8.05 (d, *J* = 8.4 Hz, 2H, , aromatic-*H*), 7.84 (d, *J* = 8.4 Hz, 2H, aromatic-*H*), 7.50 (dd, *J* = 8.0, 1.6 Hz, 1H, aromatic-*H*), 7.44 (d, *J* = 8.4 Hz, 1H, aromatic-*H*), 7.39 (d, *J* = 1.6 Hz, 1H, aromatic-*H*), 3.60 (s, 3H, -OCH_3) ppm (Figure S20); ^{13}C NMR ($CDCl_3$, 100 MHz): δ = 190.71 (*C*HO), 152.22, 142.43, 139.50, 136.12, 135.69, 129.14, 126.06, 126.02, 124.83, 124.43, 111.07 (aromatic-*C* and CF_3), 55.67 (-OCH_3) ppm (Figure S21); Anal. Calcd. for $C_{15}H_{11}F_3O_5S$: C, 50.00; H, 3.08; S, 8.90%. Found: C, 50.18; H, 3.13; S, 8.99%.

5-(Diethylamino)-2-formylphenyl 4-(trifluoromethyl)benzenesulfonate (8)

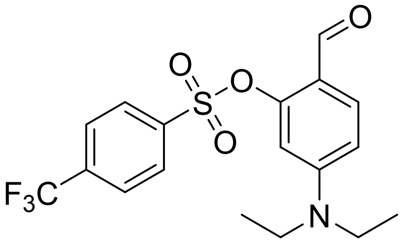

Beige color solid; yield 79%; m.p.: 114-116 °C; FT-IR/ATR: υ_{max} = 3103, 3053 (C-H str.)$_{aromatic}$, 2975, 2933 (C-H str.)$_{aliphatic}$, 2863, 2771 (C-H str.)$_{aldehyde}$, 1668 (C=O str.), 1379 (O=S=O str.)$_{antisymmetric}$, 1173 (O=S=O str.)$_{symmetric}$ cm^{-1} (Figure S22); ^{1}H NMR ($CDCl_3$, 400 MHz): δ = 9.69 (s, 1H, -C*H*=O), 8.08 (d, *J* = 8.4 Hz, 2H, aromatic-*H*), 7.85 (d, *J* = 8.4 Hz, 2H aromatic-*H*), 7.72 (d, *J* = 8.8 Hz, 1H, aromatic-*H*), 6.59 (d, *J* = 9.2 Hz, 1H, aromatic-*H*), 6.28 (s, 1H, aromatic-*H*), 3.38 (q, 4H, *J* = 7.0 Hz, –N(CH_2CH$_3$)$_2$), 1.18 (t, *J* = 7.0 Hz, 6H, –

N(CH_2CH_3)$_2$) ppm (Figure S23); ^{13}C NMR ($CDCl_3$, 100 MHz): δ = 185.08 (*C*HO), 152.97, 138.58, 135.96, 131.08, 129.18, 126.53, 126.49, 126.46, 116.55, 109.97, 104.12 (aromatic-*C* and *C*F_3), 44.94 (–N(*C*H_2CH_3)$_2$), 12.32 (–N(CH_2*C*H_3)$_2$) ppm (Figure S24); Anal. Calcd. for $C_{18}H_{18}F_3NO_4S$: C, 53.86; H, 4.52; N, 3.49; S, 7.99%. Found: C, 53.98; H, 4.43; N, 3.54; S, 8.05%.

1-Formylnaphthalen-2-yl 4-(trifluoromethyl)benzenesulfonate (9)

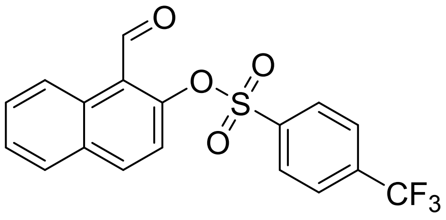

Light yellow color solid; yield 82%; m.p.: 129-131 °C; FT-IR/ATR: υ_{max} = 3107, 3062 (C-H str.)$_{aromatic}$, 2804, 2709 (C-H str.)$_{aldehyde}$, 1683 (C=O str.), 1378 (O=S=O str.)$_{antisymmetric}$, 1174 (O=S=O str.)$_{symmetric}$ cm^{-1} (Figure S25); ^{1}H NMR ($CDCl_3$, 400 MHz): δ = 10.49 (s, 1H, -C*H*=O), 9.16 (d, *J* = 8.7 Hz, 1H, naphthyl-*H*), 8.12 (d, *J* = 9.0 Hz, 1H, naphthyl-*H*), 8.07 (d, *J* = 8.2 Hz, 2H, aromatic-*H*), 7.91 (d, *J* = 8.2 Hz, 1H, aromatic-*H*), 7.88 (d, *J* = 8.3 Hz, 2H, naphthyl-*H*), 7.77 – 7.68 (m, 1H, naphthyl-*H*), 7.67 – 7.59 (m, 1H, naphthyl-*H*), 7.34 (d, *J* = 9.0 Hz, 1H, naphthyl-*H*) ppm (Figure S26); ^{13}C NMR ($CDCl_3$, 100 MHz): δ = 189.46 (*C*HO), 152.40, 138.05, 136.79, 132.27, 130.71, 130.18, 129.19, 128.45, 127.48, 126.85, 125.69, 124.21, 123.10, 121.50, 120.84 (aromatic-*C*, *C*F_3 and naphthyl-*C*) ppm (Figure S27); Anal. Calcd. for $C_{18}H_{11}F_3O_4S$: C, 56.84; H, 2.92; S, 8.43%. Found: C, 56.95; H, 2.97; S, 8.39%.

***N*'-[2-{[4-(Trifluoromethyl)benzensulfonyl]oxy}benzylidene]isonicotinohydrazide (10)**

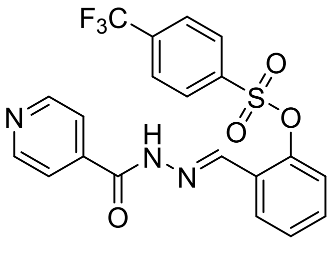

White color solid; yield 79%; m.p.: 204-206 °C; FT-IR/ATR: υ_{max} =3230 (N-H str.), 3085, 3001 (C-H str.)$_{aromatic}$, 1652 (C=O str.), 1602 (C=N str.), 1386 (O=S=O str.)$_{antisymmetric}$, 1170 (O=S=O str.)$_{symmetric}$ cm^{-1} (Figure S28); ^{1}H NMR (CDCl$_3$, 400 MHz): δ = 12.07 (s, 1H, -CONH), 8.82 (d, *J* = 6.0 Hz, 2H, pyridine-*H*), 8.39 (s, 1H, -CH=N), 8.08 – 8.03 (d, 4H, aromatic-*H*), 7.92 (dd, *J* = 8.2, 1.7 Hz, 1H, aromatic-*H*), 7.81 (d, *J* = 6.0 Hz, 2H, pyridine-*H*), 7.55 – 7.46 (m, 2H, aromatic-*H*), 7.20 (d, *J* = 8.1 Hz, 1H aromatic-*H*) ppm (Figure S29); ^{13}C NMR (CDCl$_3$, 100 MHz): δ = 161.99 (*C*=O), 147.75 (*C*=N), 150.86, 142.22, 140.57, 137.73, 135.24, 132.27, 130.06, 128.81, 127.96, 127.53, 127.49, 127.24, 123.66, 121.94 (aromatic-*C*, *C*F$_3$, and pyridine-*C*) ppm (Figure S30); Anal. Calcd. for $C_{20}H_{14}F_3N_3O_4S$: C, 53.45; H, 3.14; N, 9.35; S, 7.13%. Found: C, 53.51; H, 3.18; N, 9.42; S, 7.02%.

N'-[3-{[4-(Trifluoromethyl)benzensulfonyl]oxy}benzylidene]isonicotinohydrazide (11)

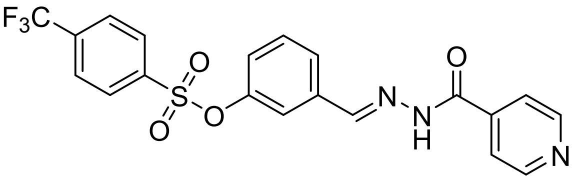

White color solid; yield 76%; m.p.: 189-190 °C; FT-IR/ATR: υ_{max} =3264 (N-H str.), 3073, 2973 (C-H str.)$_{aromatic}$, 1656 (C=O str.), 1600 (C=N str.), 1377 (O=S=O str.)$_{antisymmetric}$, 1142 (O=S=O str.)$_{symmetric}$ cm^{-1} (Figure S31); ^{1}H NMR (CDCl$_3$, 400 MHz): δ = 12.21 (s, 1H, -CONH), 8.80 (d, *J* = 4.8 Hz, 2H, pyridine-*H*), 8.42 (s, 1H, -CH=N), 8.15 (d, *J* = 7.6 Hz, 2H, aromatic-*H*), 8.08 (d, *J* = 8.2 Hz, 2H, aromatic-*H*), 7.82 (d, *J* = 4.8 Hz, 2H, pyridine-*H*), 7.73 (d, *J* = 6.4 Hz, 1H, aromatic-*H*), 7.56 (s, 1H, aromatic-*H*), 7.53 – 7.47 (m, 1H, aromatic-*H*), 7.12 (d, *J* = 5.2 Hz, 1H, aromatic-*H*) ppm (Figure S32); ^{13}C NMR (CDCl$_3$, 100 MHz): δ = 162.24 (*C*=O), 147.59 (*C*=N), 150.84, 149.59, 140.72, 138.52, 136.83, 135.05, 131.30, 129.88, 127.58, 127.54, 127.25, 124.04, 122.01, 120.58 (aromatic-*C*, *C*F$_3$, and pyridine-*C*) ppm (Figure S33); Anal. Calcd. for $C_{20}H_{14}F_3N_3O_4S$: C, 53.45; H, 3.14; N, 9.35; S, 7.13%. Found: C, 53.38; H, 3.11; N, 9.39; S, 7.17%

***N*'-[4-{[4-(Trifluoromethyl)benzensulfonyl]oxy}benzylidene]isonicotinohydrazide (12)**

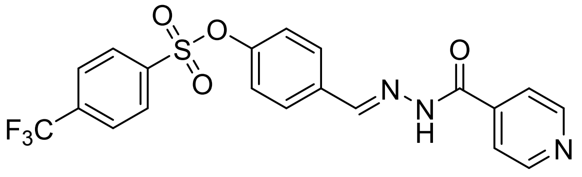

White color solid; yield 81%; m.p.: 208-209 °C; FT-IR/ATR: υ_{max} =3244 (N-H str.), 3081, 3030 (C-H str.)$_{aromatic}$, 1655 (C=O str.), 1600 (C=N str.), 1361 (O=S=O str.)$_{antisymmetric}$, 1150 (O=S=O str.)$_{symmetric}$ cm^{-1} (Figure S34); ^{1}H NMR (CDCl$_3$, 400 MHz): δ = 12.18 (s, 1H, -CONH), 8.82 – 8.78 (m, 2H, pyridine-*H*), 8.44 (s, 1H, -CH=N), 8.14 – 8.07 (m, 4H, aromatic-*H*), 7.85 – 7.76 (m, 4H, aromatic-*H* and pyridine-*H*), 7.20 (d, *J* = 8.4 Hz, 2H, aromatic-*H*) ppm (Figure S35); ^{13}C NMR (CDCl$_3$, 100 MHz): δ = 162.16 (*C*=O), 147.82 (*C*=N), 150.83, 150.25, 140.74, 138.48, 134.03, 129.89, 129.47, 127.60, 127.56, 123.15, 122.24, 122.00 (aromatic-*C*, *C*F$_3$, and pyridine-*C*) ppm (Figure S36); Anal. Calcd. for $C_{20}H_{14}F_3N_3O_4S$: C, 53.45; H, 3.14; N, 9.35; S, 7.13%. Found: C, 53.41; H, 3.13; N, 9.37; S, 7.08%.

***N*'-[2-{[4-(Trifluoromethyl)benzensulfonyl]oxy}-3-methoxybenzylidene]isonicotinohydrazide (13)**

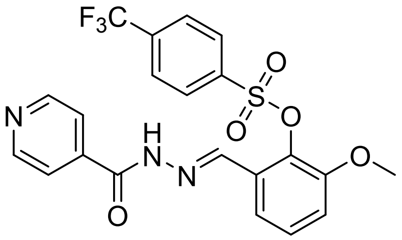

White color solid; yield 81%; m.p.: 198-199 °C; FT-IR/ATR: υ_{max} = 3448 (N-H str.), 3154, 3030 (C-H str.)$_{aromatic}$, 2900, 2843 (C-H str.)$_{aliphatic}$, 1676 (C=O str.), 1569 (C=N str.), 1378 (O=S=O str.)$_{antisymmetric}$, 1137 (O=S=O str.)$_{symmetric}$ cm^{-1} (Figure S37); ^{1}H NMR (CDCl$_3$, 400 MHz): δ = 12.29 (s, 1H, -CONH), 8.84 – 8.78 (m, 2H, pyridine-*H*), 8.60 (s, 1H, -CH=N), 8.11 – 8.07 (m, 4H, aromatic-*H*), 7.83 (d, *J* = 6.0, 2H, pyridine-*H*), 7.59 (dd, *J* = 8.0, 1.2 Hz, 1H, aromatic-*H*), 7.40 (t, *J* = 8.0 Hz, 1H, aromatic-*H*), 7.17 (d, *J* = 8.0 Hz, 1H, aromatic-*H*), 3.37 (s, 3H, -OC*H*$_3$) ppm (Figure S38); ^{13}C NMR (CDCl$_3$, 100 MHz): δ = 162.19 (*C*=O), 143.14 (*C*=N), 152.03, 150.80, 140.60, 139.51, 137.17, 134.82, 129.89, 129.86, 129.06, 127.15, 127.11, 122.02, 118.12, 115.06 (aromatic-*C*, *C*F$_3$, and pyridine-*C*), 55.90 (-O*C*H$_3$) ppm (Figure

S39); Anal. Calcd. for $C_{21}H_{16}F_3N_3O_5S$: C, 52.61; H, 3.36; N, 8.76; S, 6.69%. Found: C, 52.50; H, 3.41; N, 8.69; S, 6.81%.

N'-[2-{[4-(Trifluoromethyl)benzensulfonyl]oxy}-4-methoxybenzylidene]isonicotinohydrazide (14)

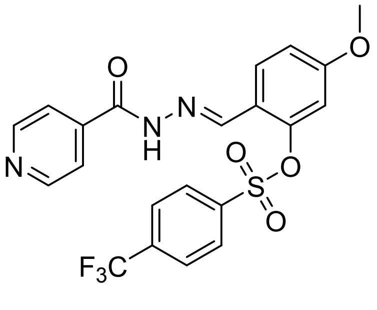

White color solid; yield 75%; m.p.: 180-181 °C; FT-IR/ATR: υ_{max} = 3206 (N-H str.), 3069, 3001 (C-H str.)$_{aromatic}$, 2904, 2844 (C-H str.)$_{aliphatic}$, 1650 (C=O str.), 1607 (C=N str.), 1383 (O=S=O str.)$_{antisymmetric}$, 1129 (O=S=O str.)$_{symmetric}$ cm^{-1} (Figure S40); ^{1}H NMR ($CDCl_3$, 400 MHz): δ = 11.94 (s, 1H, -CONH), 8.81 (d, *J* = 6.0 Hz, 2H, pyridine-*H*), 8.31 (s, 1H, -CH=N), 8.12 – 8.03 (m, 4H, aromatic-*H*), 7.86 (d, *J* = 8.8 Hz, 1H, aromatic-*H*), 7.80 (d, *J* = 6.0 Hz, 2H, pyridine-*H*), 7.09 (dd, *J* = 8.8, 2.4 Hz, 1H, aromatic-*H*), 6.66 (d, *J* = 2.4 Hz, 1H, aromatic-*H*), 3.75 (s, 3H, -OCH_3) ppm (Figure S41); ^{13}C NMR ($CDCl_3$, 100 MHz): δ = 161.91 (*C*=O), 148.70 (*C*=N), 161.78, 150.82, 142.30, 140.73, 137.66, 135.28, 130.21, 128.18, 127.50, 127.47, 121.92, 120.30, 115.28, 108.78 (aromatic-*C*, *C*F_3, and pyridine-*C*), 56.25 (-O*C*H_3) ppm (Figure S42); Anal. Calcd. for $C_{21}H_{16}F_3N_3O_5S$: C, 52.61; H, 3.36; N, 8.76; S, 6.69%. Found: C, 52.63; H, 3.28; N, 8.82; S, 6.79%.

N'-[3-{[4-(Trifluoromethyl)benzensulfonyl]oxy}-4-methoxybenzylidene]isonicotinohydrazide (15)

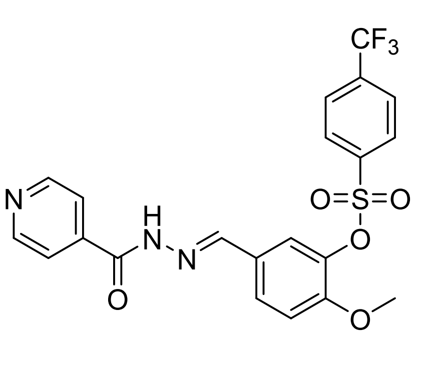

White color solid; yield 79%; m.p.: 143-145 °C; FT-IR/ATR: υ_{max} =3227 (N-H str.), 3161, 3029 (C-H str.)$_{aromatic}$, 2967, 2845 (C-H str.)$_{aliphatic}$, 1670 (C=O str.), 1613 (C=N str.), 1379 (O=S=O str.)$_{antisymmetric}$, 1138 (O=S=O str.)$_{symmetric}$ cm^{-1} (Figure S43); ^{1}H NMR ($CDCl_3$, 400 MHz): δ = 12.11 (s, 1H, -CONH), 8.83 – 8.77 (m, 2H, pyridine-*H*), 8.40 (s, 1H, -CH=N), 8.10 – 8.05 (m, 4H, aromatic-*H*), 7.85 – 7.79 (m, 2H, aromatic-*H*), 7.72 – 7.66 (m, 2H, pyridine-*H*), 7.16 (d, *J* = 9.2 Hz, 1H, aromatic-*H*), 3.46 (s, 3H, -OCH_3) ppm (Figure S44); ^{13}C NMR ($CDCl_3$, 100 MHz): δ = 162.05 (*C*=O), 147.83 (*C*=N), 152.93, 150.82, 140.90, 139.19, 137.96, 129.88, 128.99, 127.64, 127.09, 127.05, 125.03, 122.27, 122.00, 114.10 (aromatic-*C*, *C*F$_3$, and pyridine-*C*), 56.13 (-O*C*H$_3$) ppm (Figure S45); Anal. Calcd. for $C_{21}H_{16}F_3N_3O_5S$: C, 52.61; H, 3.36; N, 8.76; S, 6.69%. Found: C, 52.68; H, 3.39; N, 8.67; S, 6.74%.

N'-[4-{[4-(Trifluoromethyl)benzensulfonyl]oxy}-3-methoxybenzylidene]isonicotinohydrazide (16)

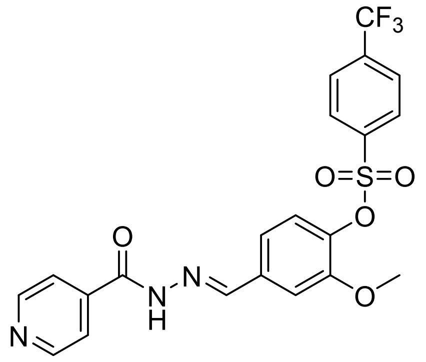

White color solid; yield 80%; m.p.: 192-193 °C; FT-IR/ATR: υ_{max} =3299 (N-H str.), 3186, 3075 (C-H str.)$_{aromatic}$, 2999, 2853 (C-H str.)$_{aliphatic}$, 1653 (C=O str.), 1578 (C=N str.), 1378 (O=S=O str.)$_{antisymmetric}$, 1131 (O=S=O str.)$_{symmetric}$ cm^{-1} (Figure S46); ^{1}H NMR ($CDCl_3$, 400 MHz): δ = 12.20 (s, 1H, -CONH), 8.80 (d, *J* = 6.0 Hz, 2H, pyridine-*H*), 8.43 (s, 1H, -CH=N), 8.08 (br.s, 4H, aromatic-*H*), 7.82 (d, *J* = 6.0 Hz, 2H, pyridine-*H*), 7.37 (d, *J* = 9.6 Hz, 2H, aromatic-*H*), 7.32 (d, *J* = 8.2 Hz, 1H, aromatic-*H*), 3.50 (s, 3H, -OCH_3) ppm (Figure S47); ^{13}C NMR ($CDCl_3$, 100 MHz): δ = 162.22 (*C*=O), 148.13 (*C*=N), 151.76, 150.84, 140.77, 139.31, 138.95, 135.14, 129.81, 127.14, 127.11, 125.04, 124.79, 122.01, 121.04, 111.10 (aromatic-*C*, *C*F$_3$, and pyridine-*C*), 55.96 (-O*C*H$_3$) ppm (Figure S48); Anal. Calcd. for $C_{21}H_{16}F_3N_3O_5S$: C, 52.61; H, 3.36; N, 8.76; S, 6.69%. Found: C, 52.60; H, 3.43; N, 8.83; S, 6.62%.

***N*'-[2-{[4-(Trifluoromethyl)benzensulfonyl]oxy}-4-(diethylamino)benzylidene]isonicotinohydrazide** (**17**)

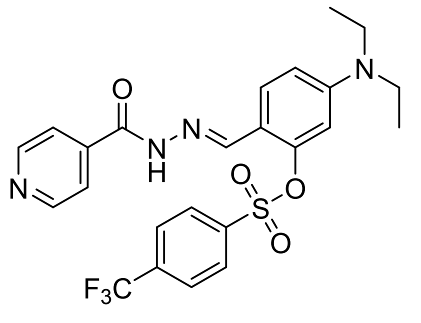

White color solid; yield 74%; m.p.: 141-142 °C; FT-IR/ATR: υ_{max} = 3218 (N-H str.), 3094, 3051 (C-H str.)$_{aromatic}$, 2973, 2930, 2904, 2853 (C-H str.)$_{aliphatic}$, 1647 (C=O str.), 1608 (C=N str.), 1376 (O=S=O str.)$_{antisymmetric}$, 1171 (O=S=O str.)$_{symmetric}$ cm^{-1} (Figure S49); ^1H NMR ($CDCl_3$, 400 MHz): δ = 11.85 (s, 1H, -CONH), 8.79 (d, *J* = 6.0 Hz, 2H, pyridine-*H*), 8.36 (s, 1H, -CH=N), 8.15 (d, *J* = 8.4 Hz, 2H, aromatic-*H*), 8.07 (d, *J* = 8.4 Hz, 2H, aromatic-*H*), 7.81 (d, *J* = 6.0 Hz, 2H, pyridine-*H*), 7.74 (d, *J* = 8.8 Hz, 1H, aromatic-*H*), 6.75 (dd, *J* = 9.2, 2.0 Hz, 1H, aromatic-*H*), 5.94 (d, *J* = 2.4 Hz, 1H, aromatic-*H*), 3.23 (q, *J* = 6.8 Hz, 4H, $–N(CH_2CH_3)_2$), 0.95 (t, *J* = 6.8 Hz, 6H, $–N(CH_2CH_3)_2$) ppm (Figure S50); ^{13}C NMR ($CDCl_3$, 100 MHz): δ = 161.49 (*C*=O), 149.80, (*C*=N), 150.74, 150.11, 143.38, 141.03, 138.44, 135.09, 130.18, 128.08, 127.52, 127.48, 121.93, 113.50, 111.65, 104.10 (aromatic-*C*, CF_3, and pyridine-*C*), 44.33 ($–N(CH_2CH_3)_2$), 12.55 ($–N(CH_2CH_3)_2$) ppm (Figure S51); Anal. Calcd. for $C_{24}H_{23}F_3N_4O_4S$: C, 55.38; H, 4.45; N, 10.76; S, 6.16%. Found: C, 55.45; H, 4.49; N, 10.84; S, 6.11%.

***N*'-[2-{[4-(Trifluoromethyl)benzensulfonyl]oxy}naphthalen-1-ylmethylene]isonicotinohydrazide** (**18**)

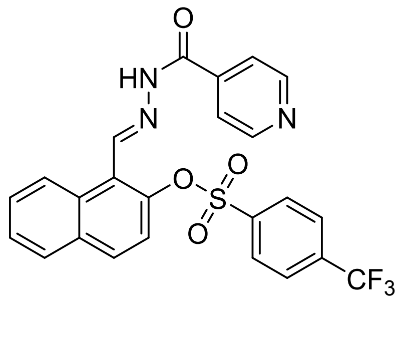

White color solid; yield 76%; m.p.: 180-181 °C; FT-IR/ATR: υ_{max} = 3288 (N-H str.), 3166, 3020 (C-H str.)$_{aromatic}$, 1648 (C=O str.), 1603 (C=N str.), 1379 (O=S=O str.)$_{antisymmetric}$, 1172 (O=S=O str.)$_{symmetric}$ cm^{-1} (Figure S52); ^{1}H NMR ($CDCl_3$, 400 MHz): δ = 12.06 (s, 1H, -CONH), 9.16 (s, 1H, -CH=N), 8.86 (br.s, 2H, pyridine-*H*), 8.68 (br.s, 1H, naphthyl-*H*), 8.15 – 7.99 (m, 7H, naphthyl-*H* and aromatic-*H*), 7.87 (br.s, 2H, pyridine-*H*), 7.68 (br.s, 1H, naphthyl-*H*), 7.45 (br.s, 1H, naphthyl-*H*) ppm (Figure S53); ^{13}C NMR ($CDCl_3$, 100 MHz): δ = 161.86 (*C*=O), 147.39 (*C*=N), 150.91, 150.04, 143.82, 140.47, 137.65, 135.19, 134.87, 133.13, 132.73, 130.43, 130.11, 129.20, 127.55, 127.44, 127.06, 122.33, 121.98, 121.68 (aromatic-*C*, *C*F_3, pyridine-*C* and naphthyl-*C*) ppm (Figure S54); Anal. Calcd. for $C_{24}H_{16}F_3N_3O_4S$: C, 57.71; H, 3.23; N, 8.41; S, 6.42%. Found: C, 57.66; H, 3.25; N, 8.42; S, 6.46%.

2. FT-IR, ^{1}H- and ^{13}C- NMR spectra of the synthesized compounds

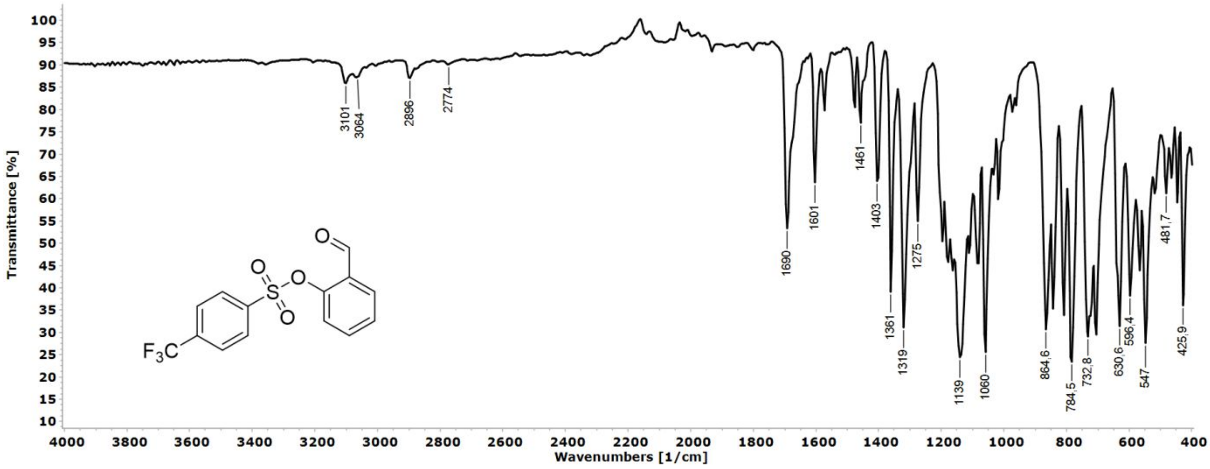

Figure S1. FT-IR spectrum of compound **1**

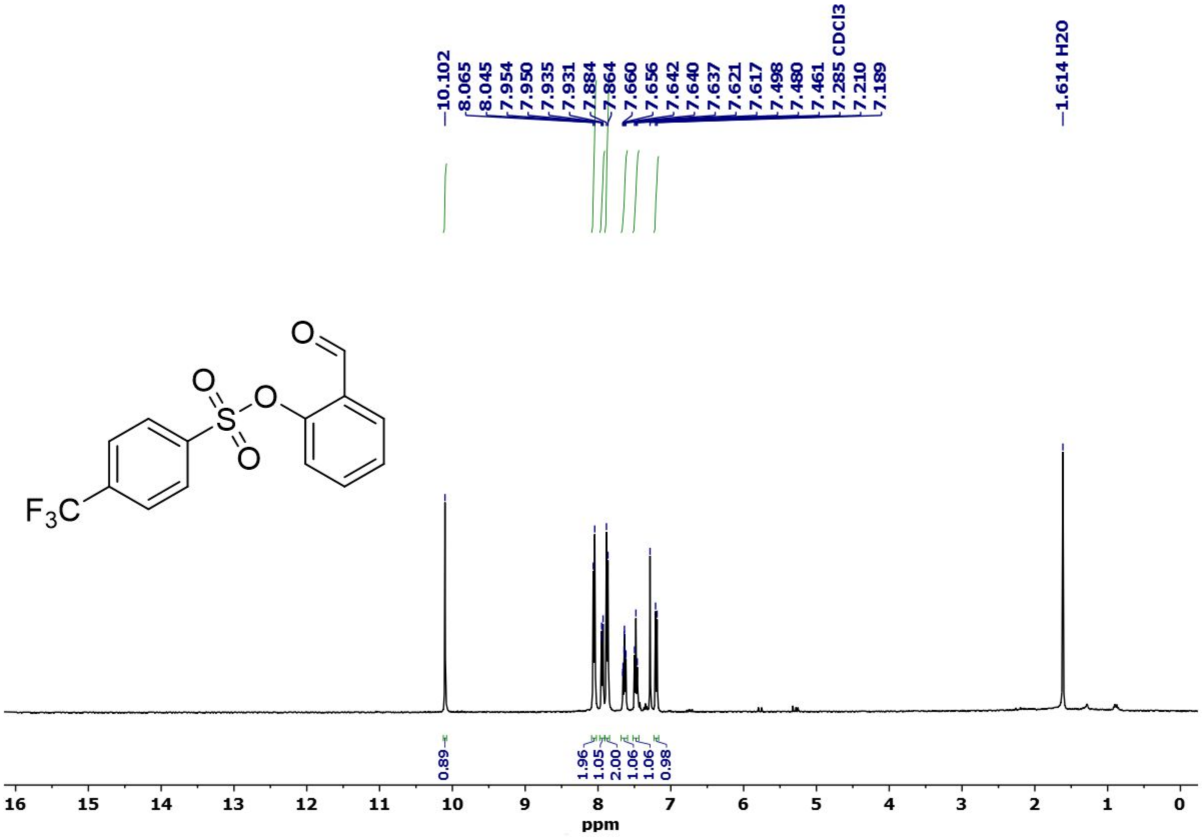

Figure S2. ^{1}H NMR spectrum of compound **1**

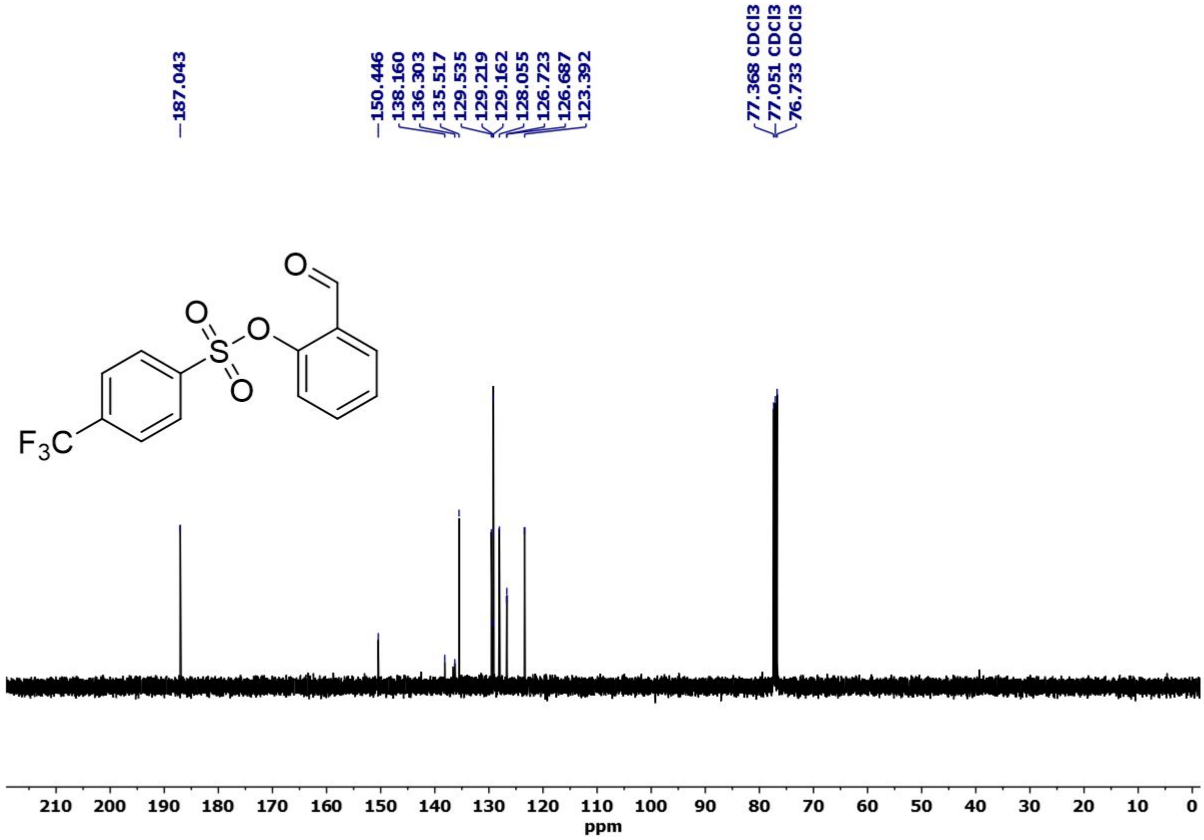

Figure S3. ^{13}C NMR spectrum of compound **1**

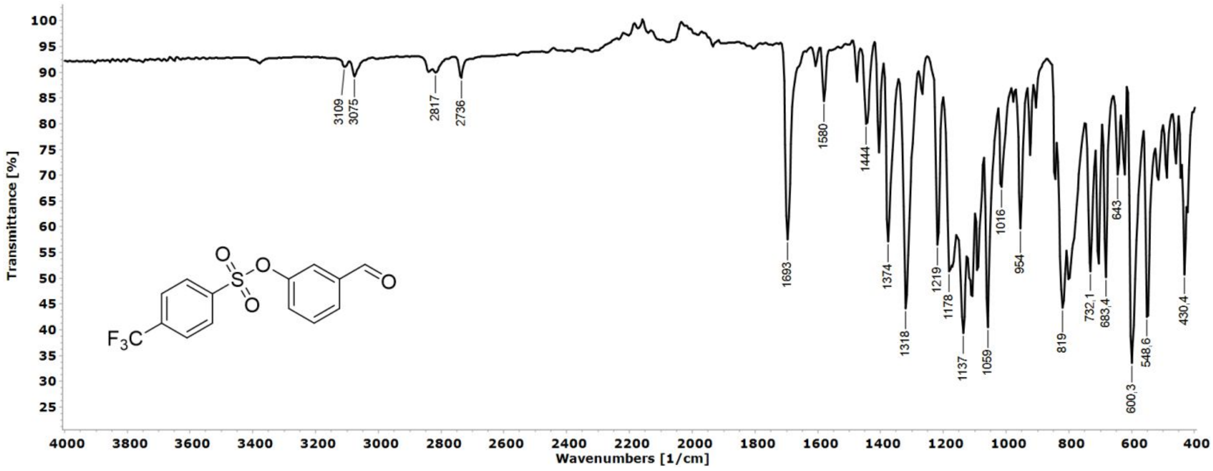

Figure S4. FT-IR spectrum of compound **2**

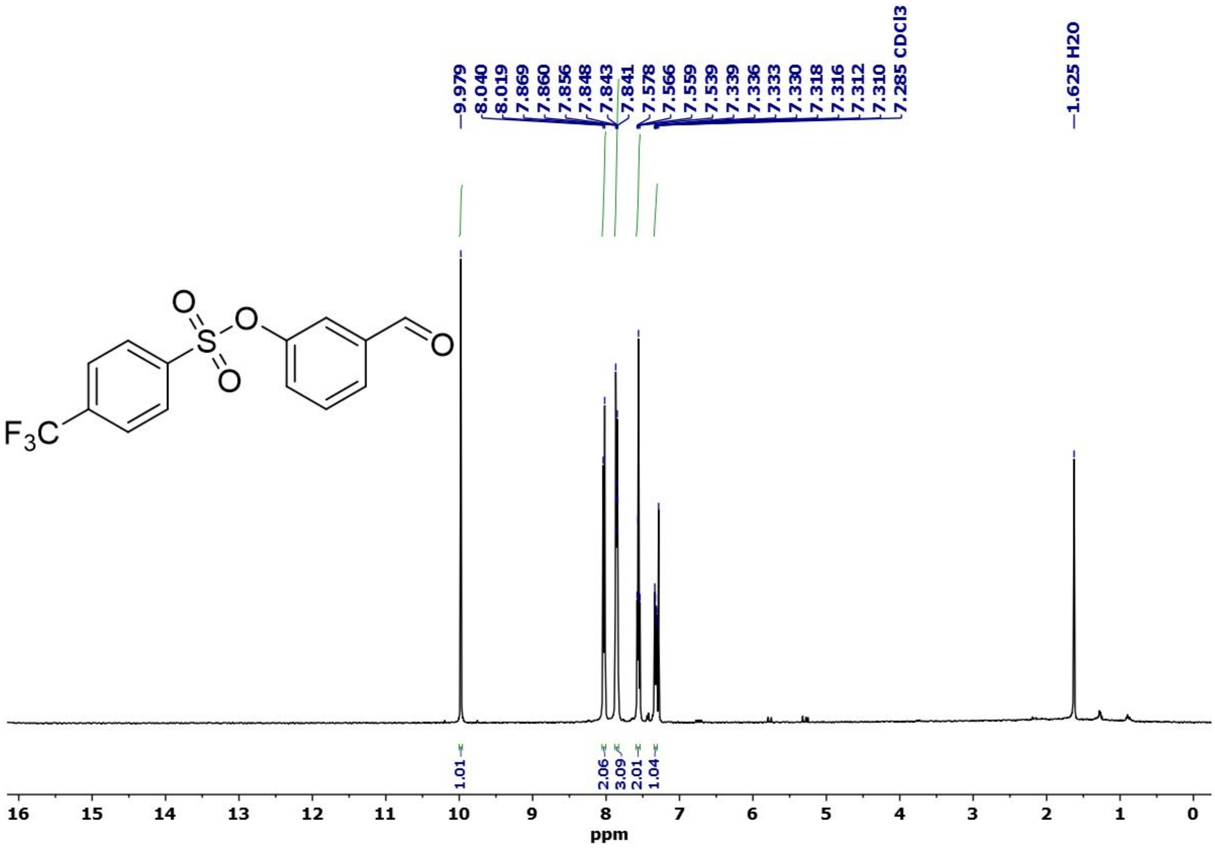

Figure S5. ^{1}H NMR spectrum of compound **2**

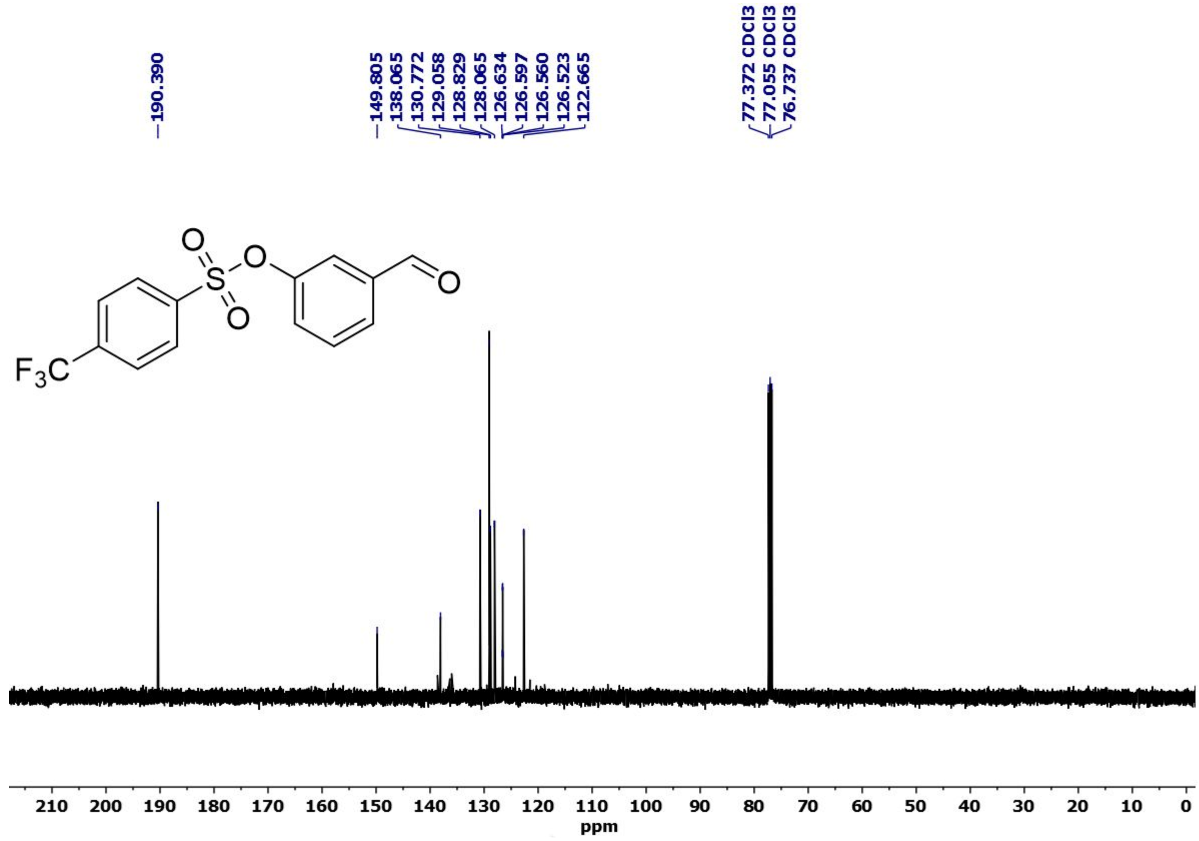

Figure S6. ^{13}C NMR spectrum of compound **2**

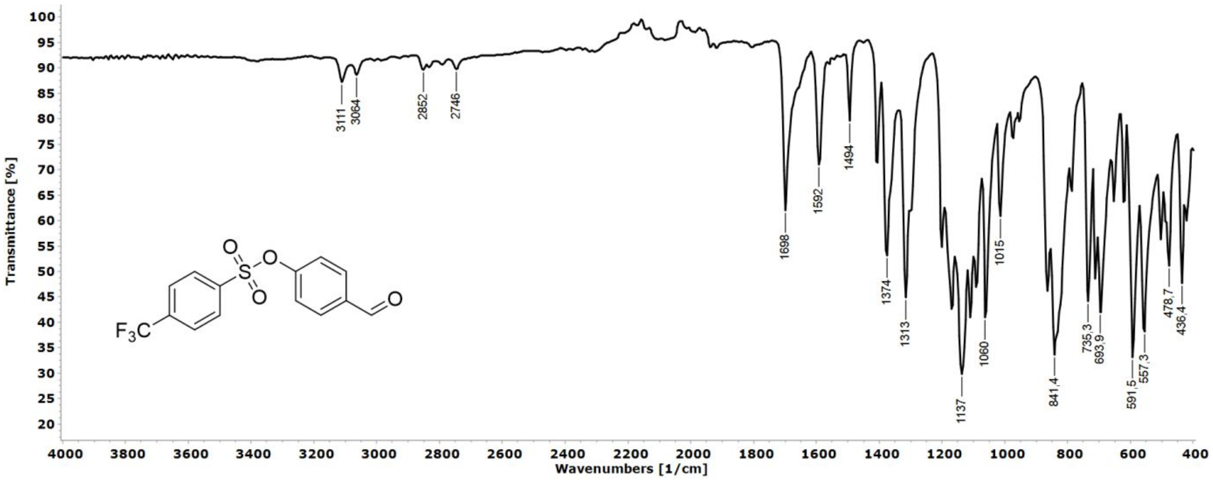

Figure S7. FT-IR spectrum of compound **3**

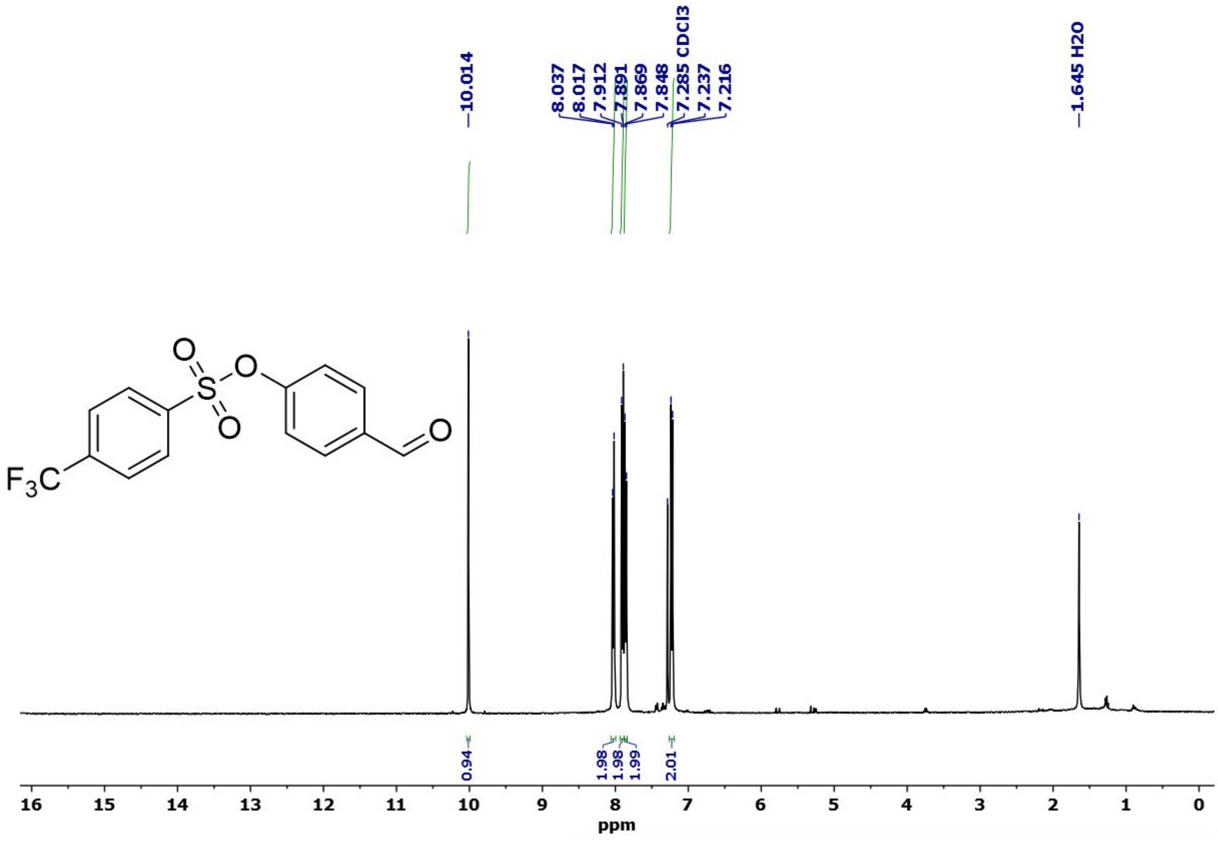

Figure S8. ^{1}H NMR spectrum of compound **3**

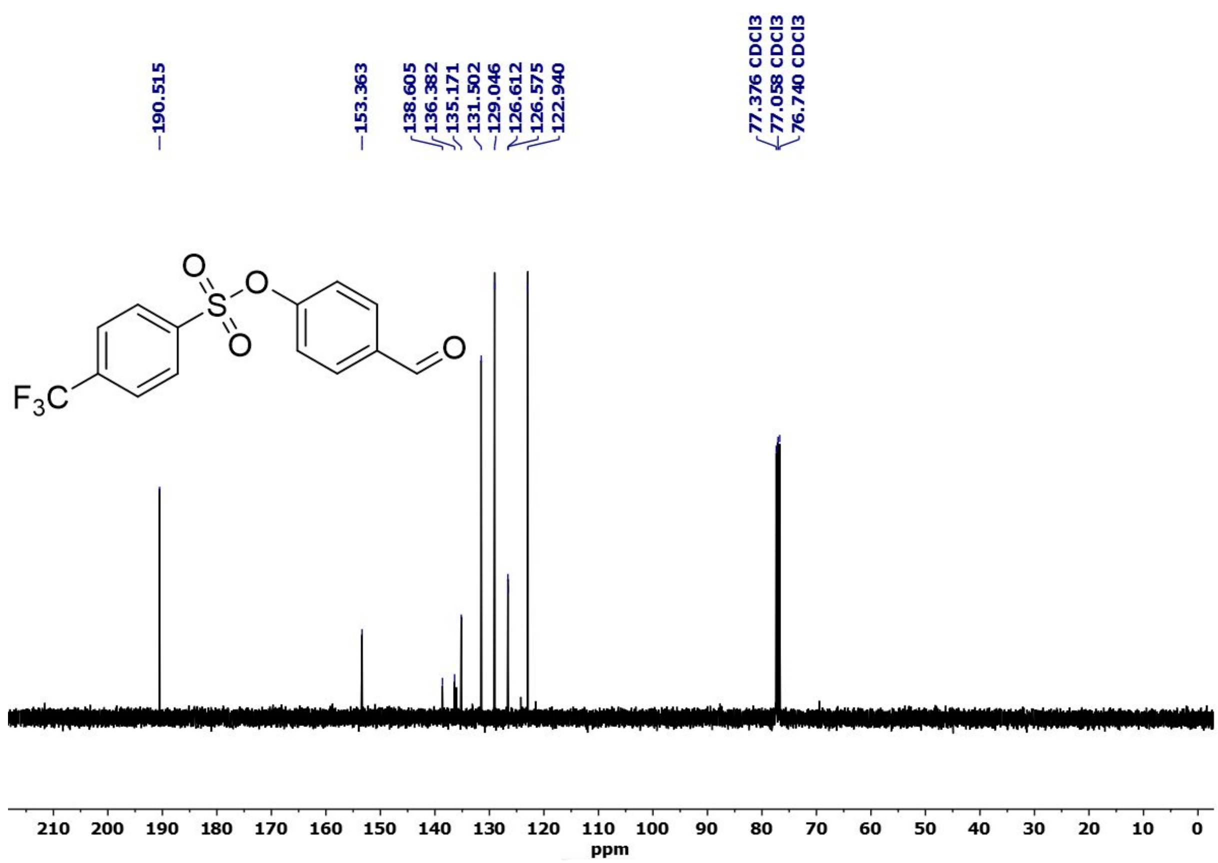

Figure S9. ^{13}C NMR spectrum of compound **3**

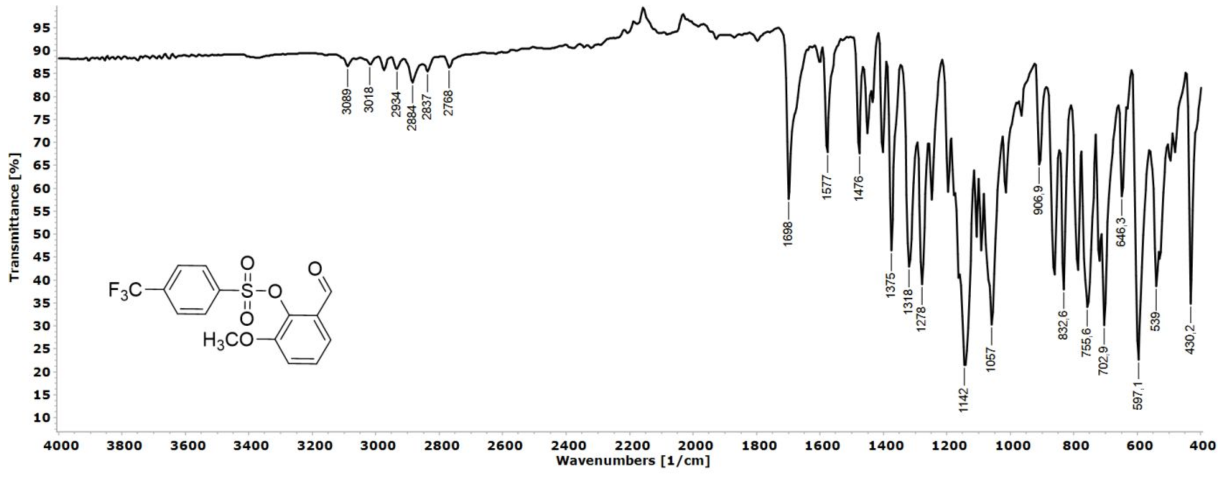

Figure S10. FT-IR spectrum of compound **4**

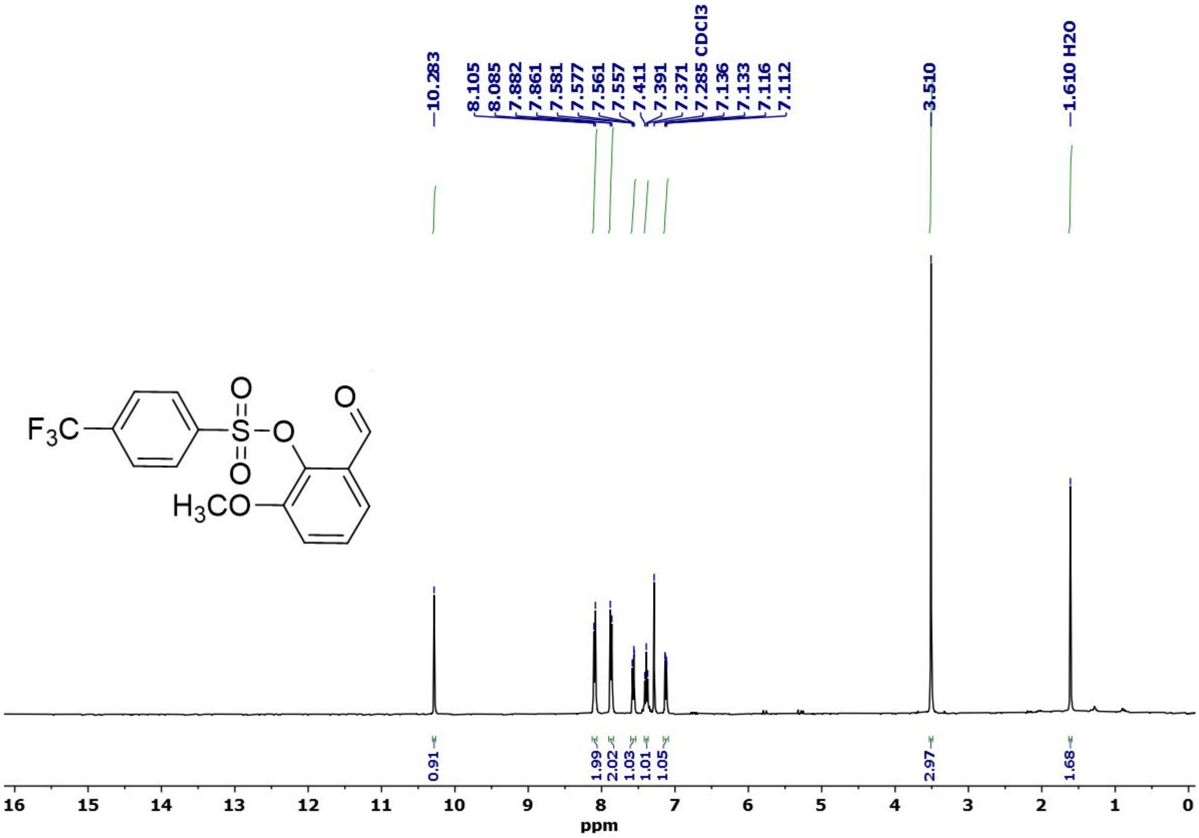

Figure S11. ^{1}H NMR spectrum of compound **4**

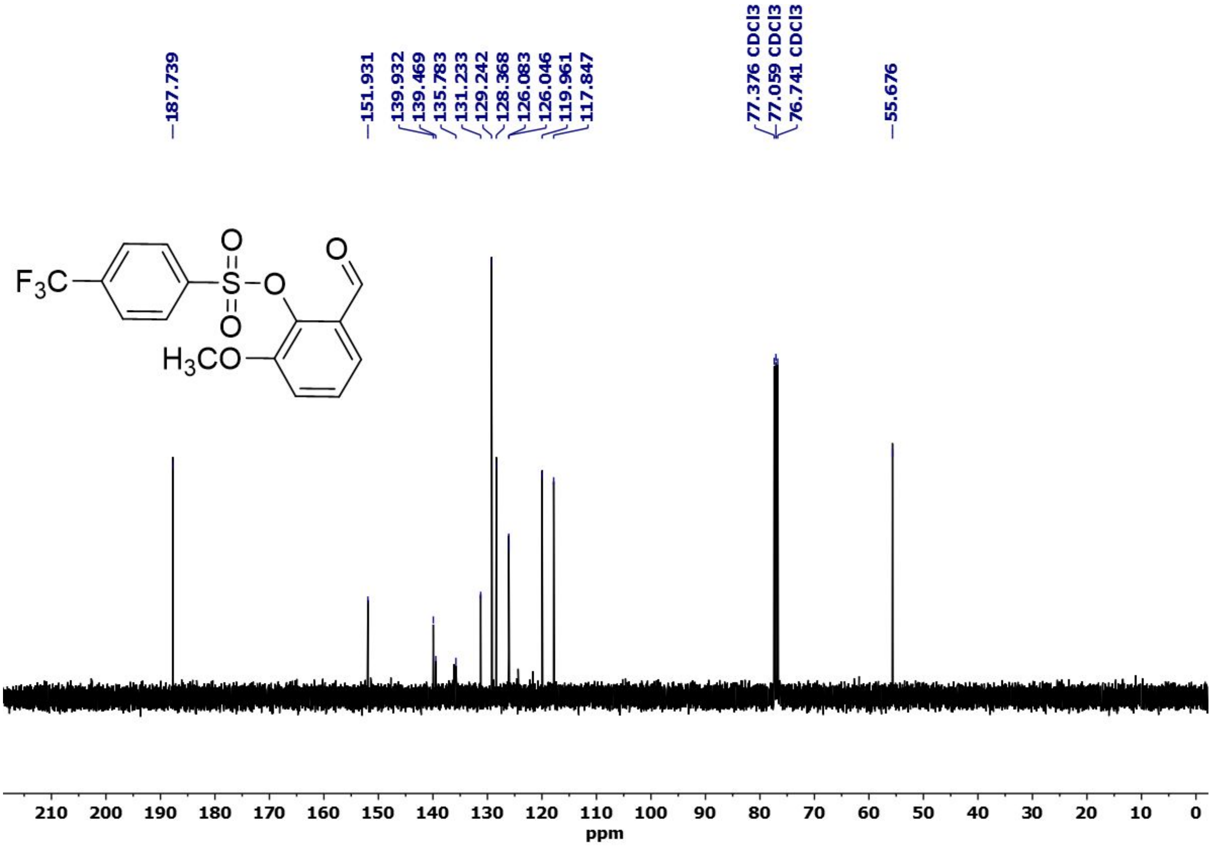

Figure S12. ^{13}C NMR spectrum of compound **4**

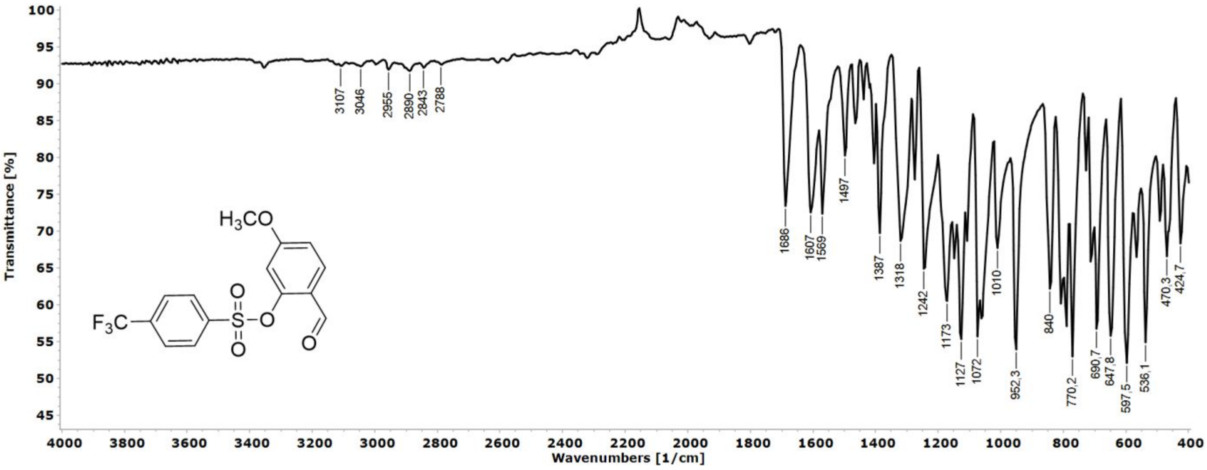

Figure S13. FT-IR spectrum of compound **5**

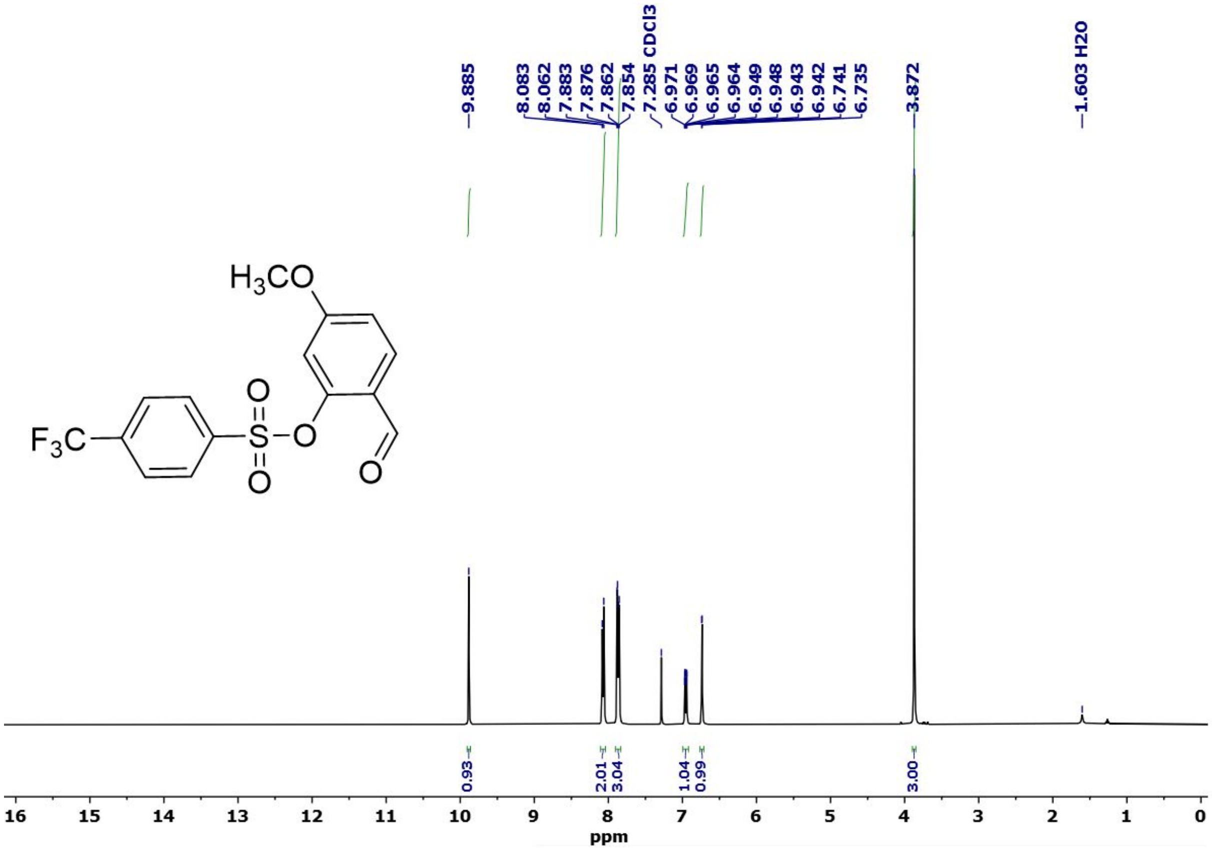

Figure S14. ^{1}H NMR spectrum of compound **5**

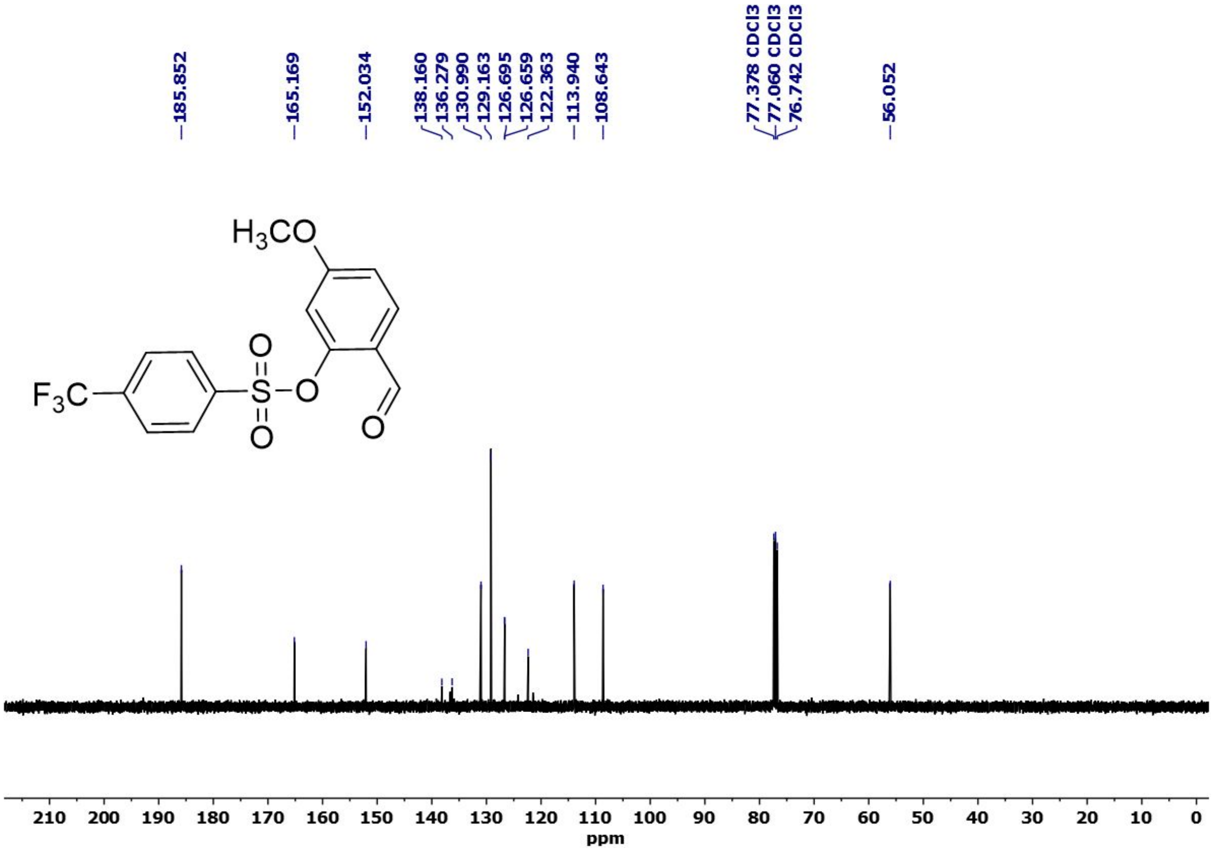

Figure S15. ^{13}C NMR spectrum of compound **5**

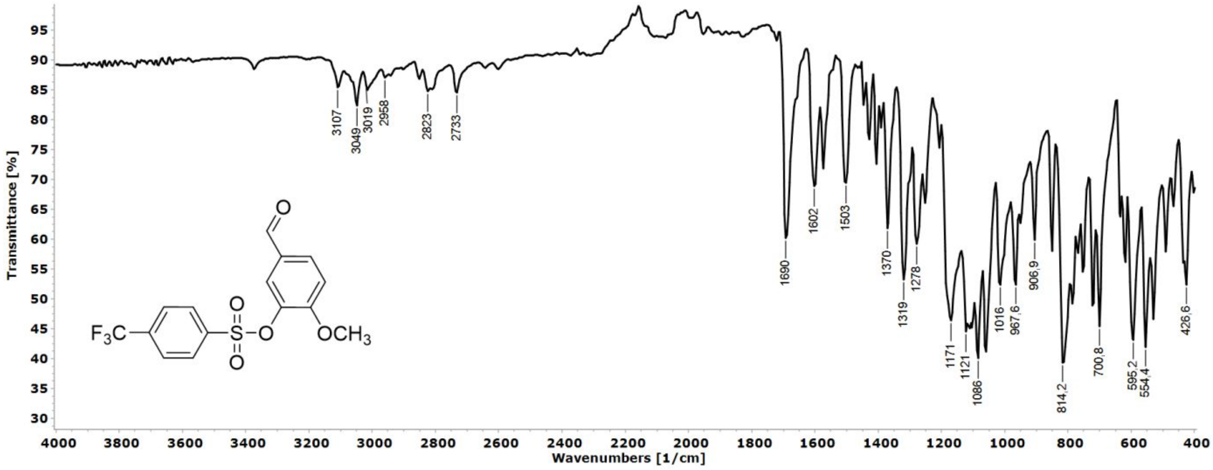

Figure S16. FT-IR spectrum of compound **6**

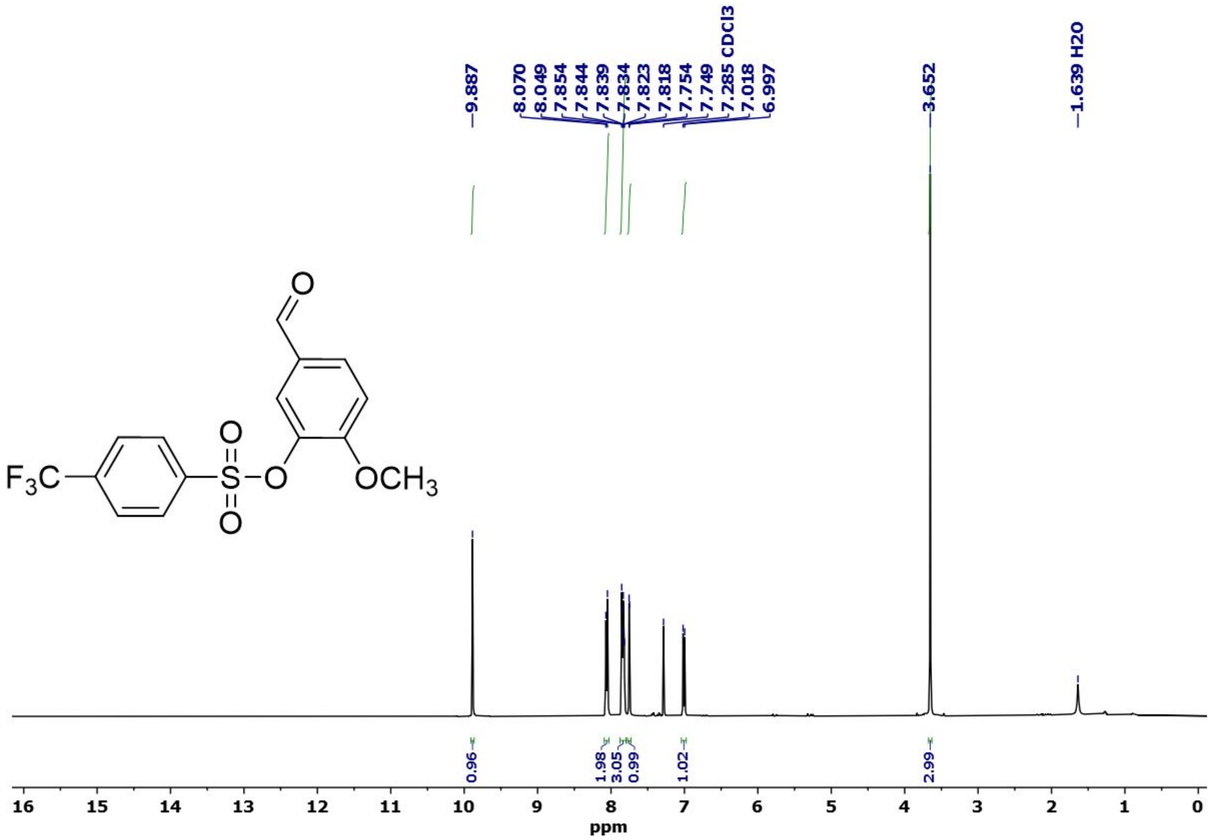

Figure S17. ^{1}H NMR spectrum of compound **6**

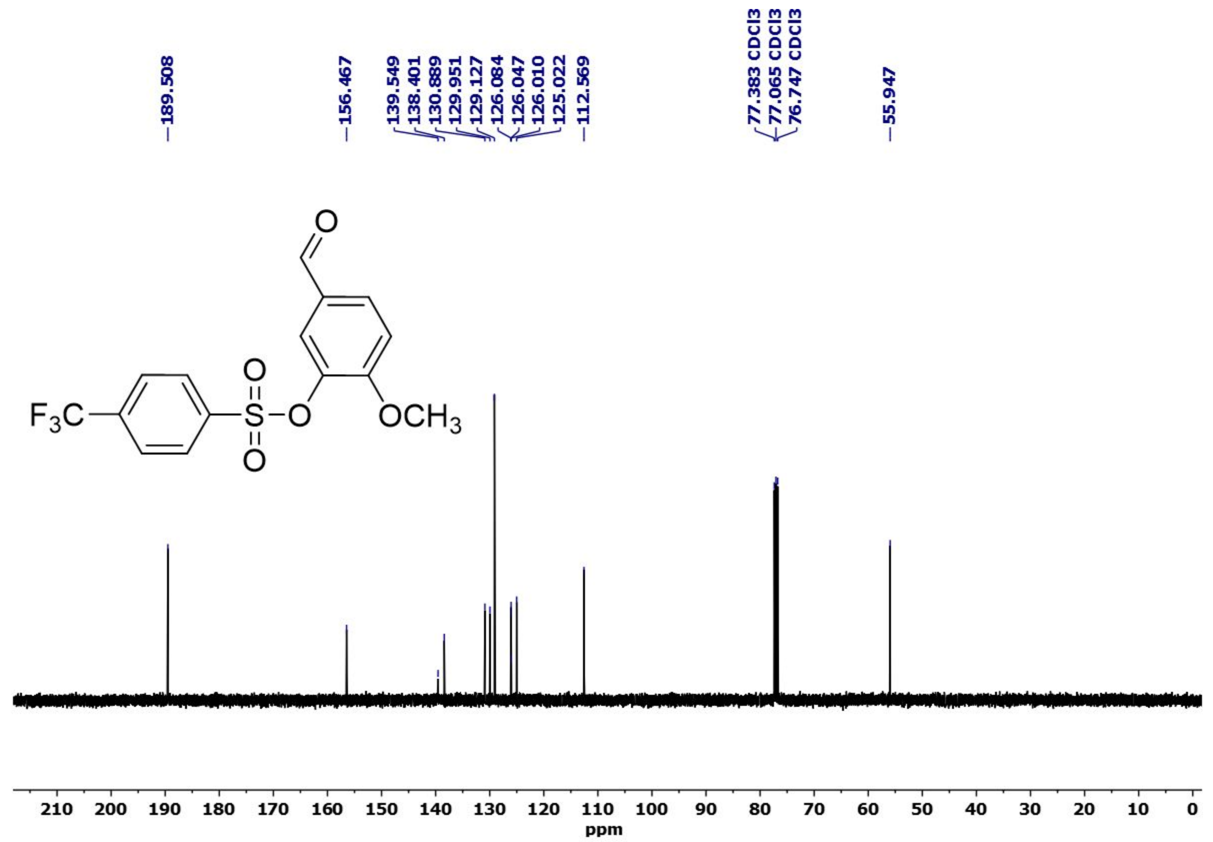

Figure S18. ^{13}C NMR spectrum of compound **6**

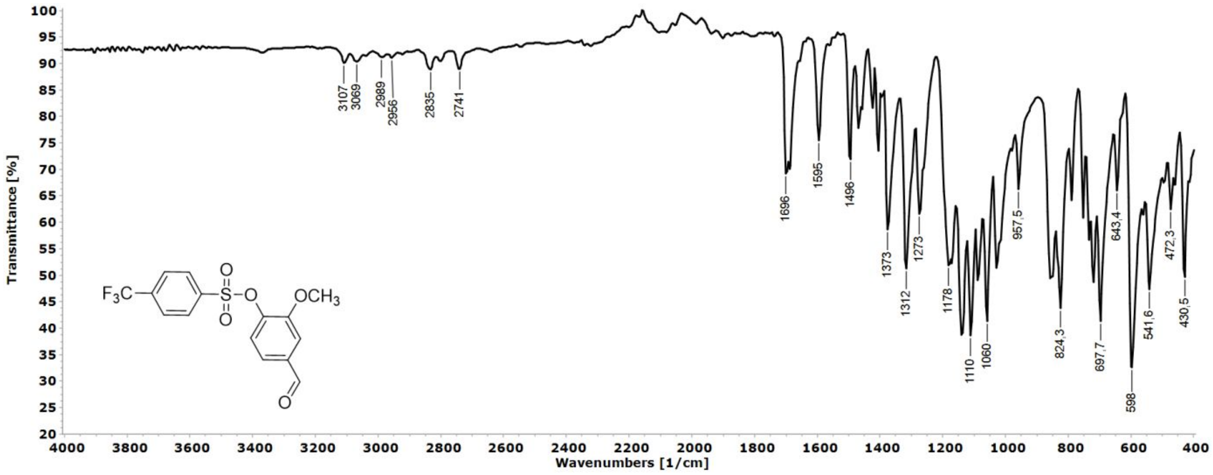

Figure S19. FT-IR spectrum of compound **7**

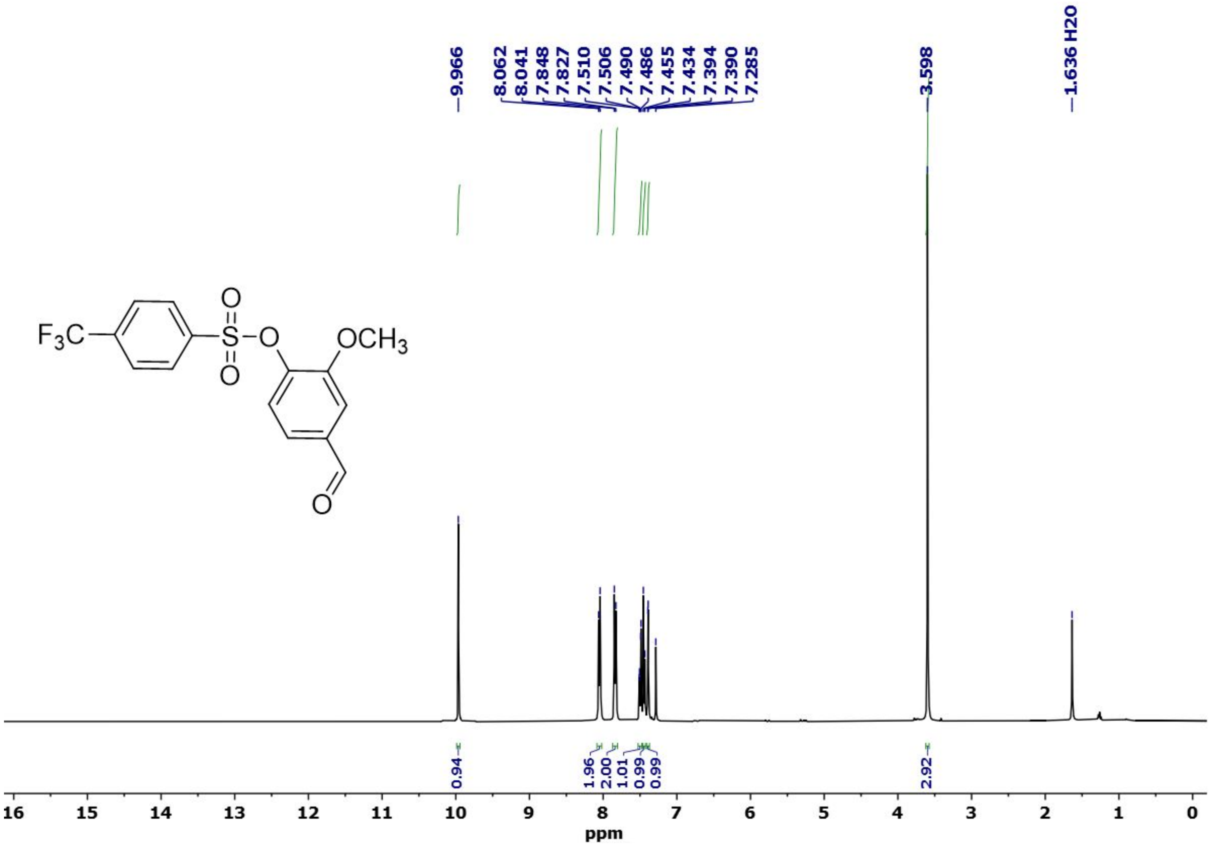

Figure S20. ^{1}H NMR spectrum of compound **7**

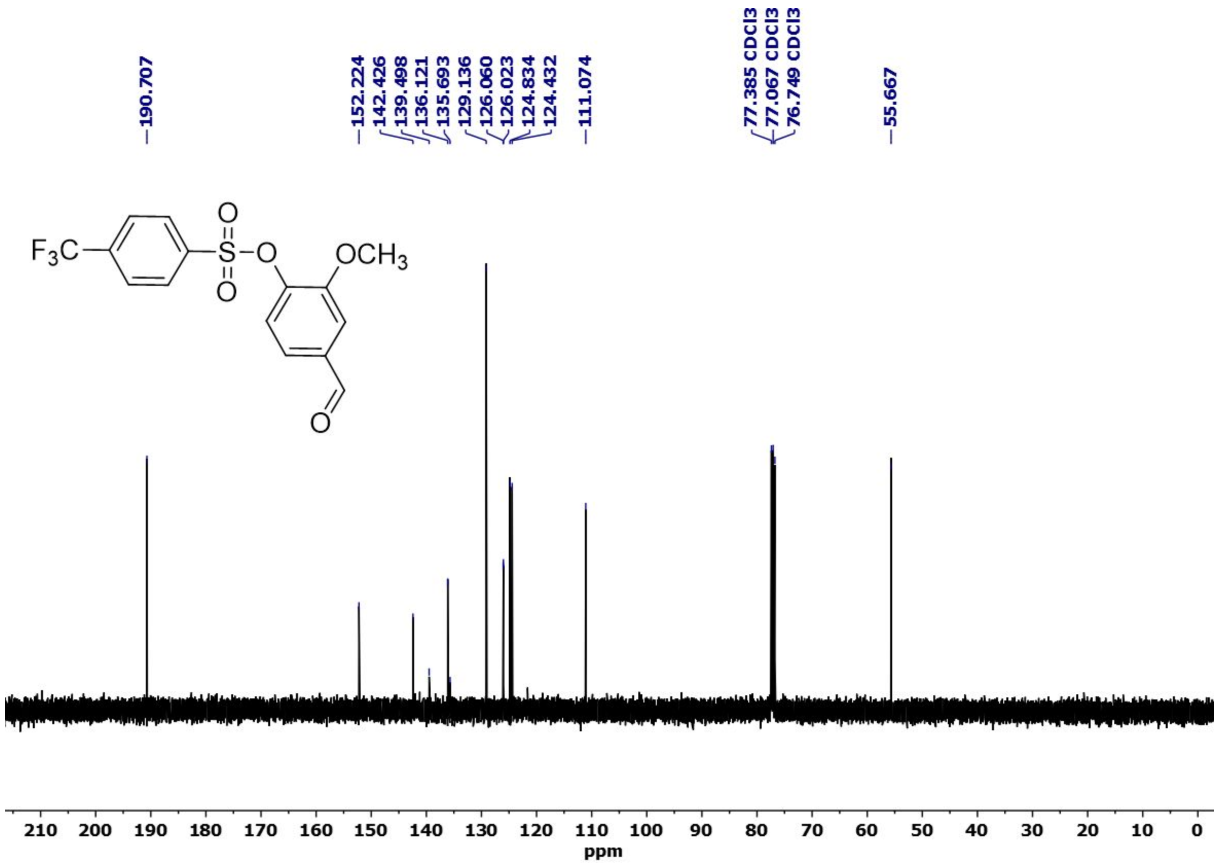

Figure S21. ^{13}C NMR spectrum of compound **7**

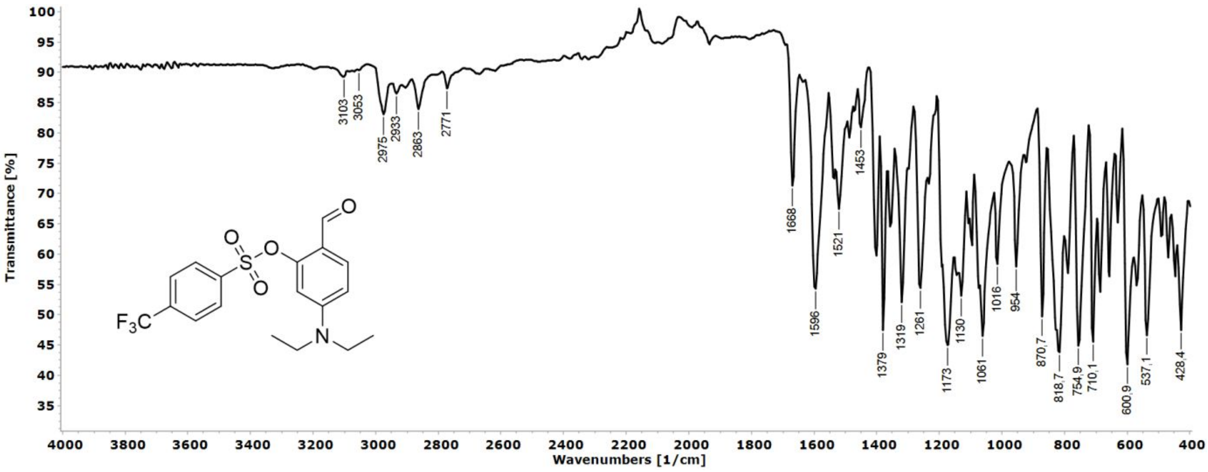

Figure S22. FT-IR spectrum of compound **8**

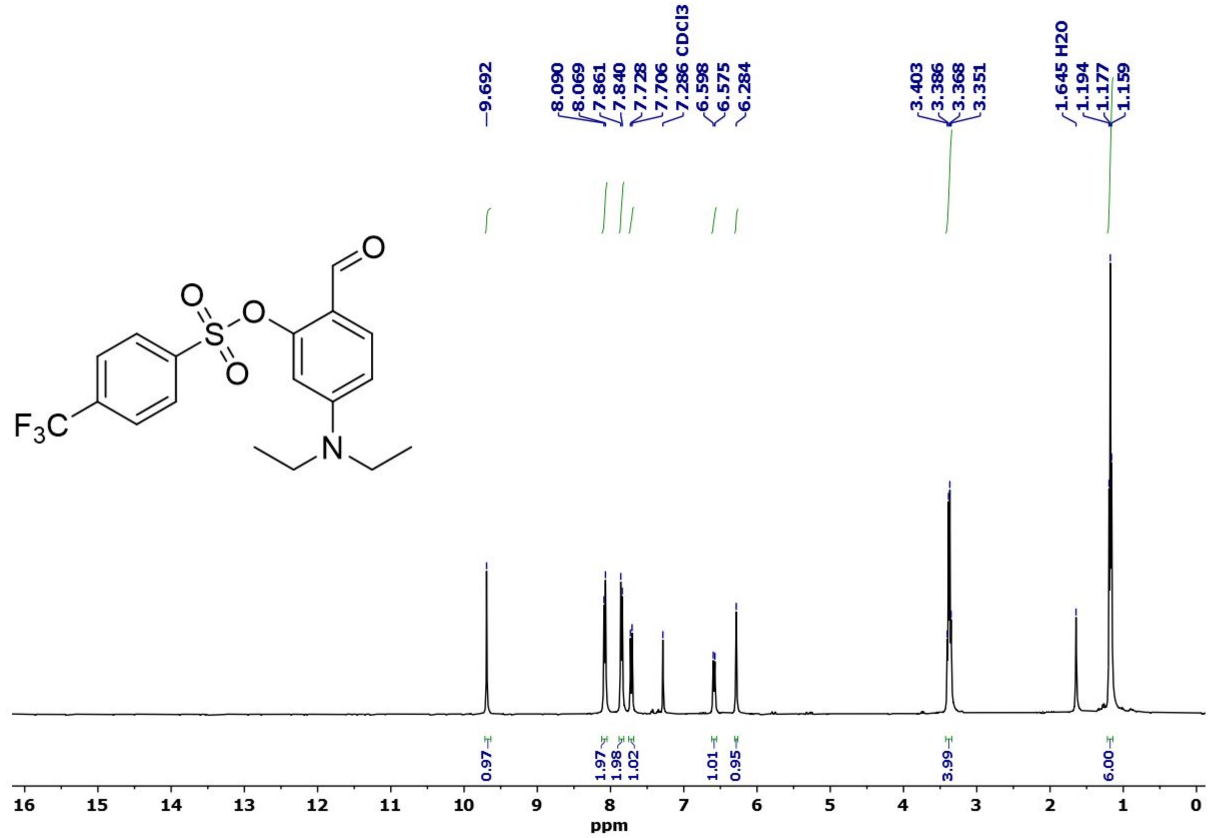

Figure S23. ^{1}H NMR spectrum of compound **8**

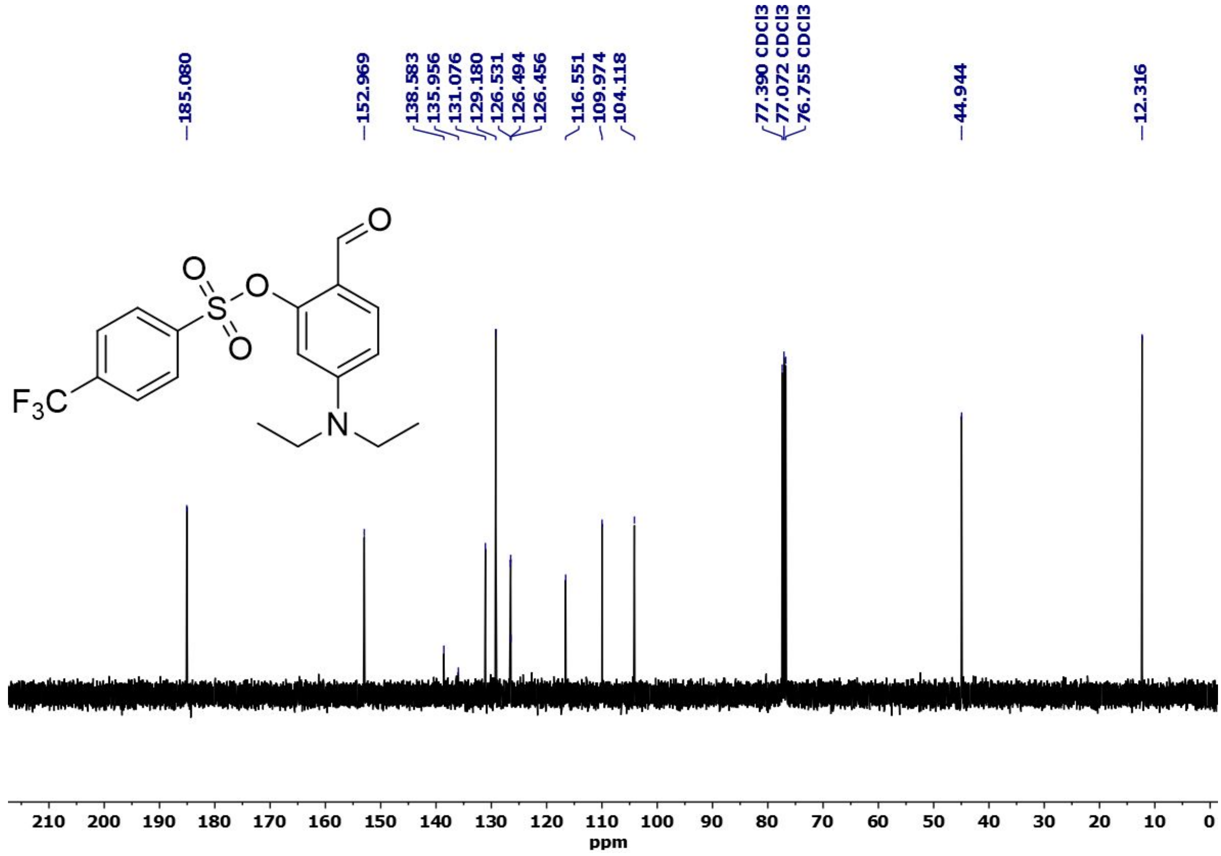

Figure S24. ^{13}C NMR spectrum of compound **8**

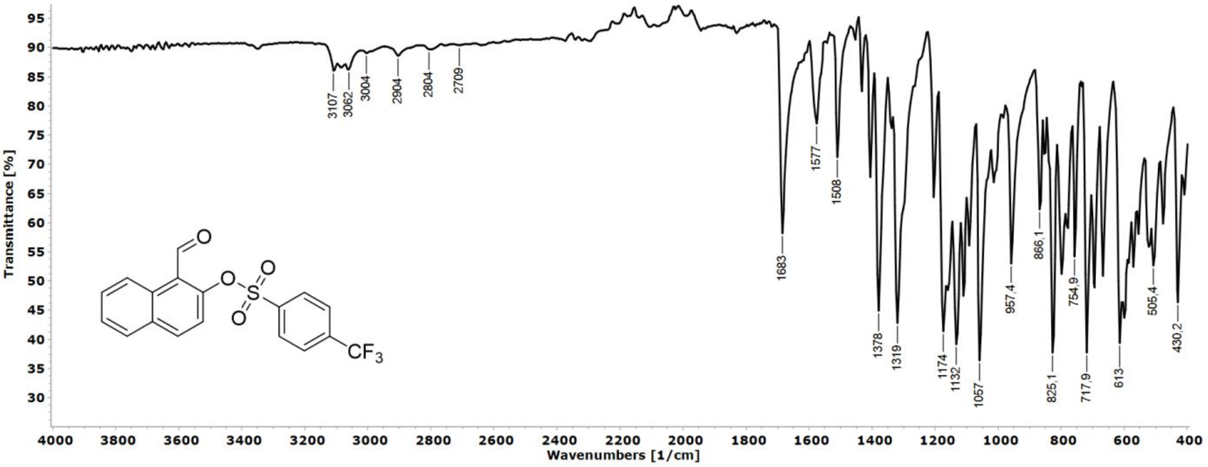

Figure S25. FT-IR spectrum of compound **9**

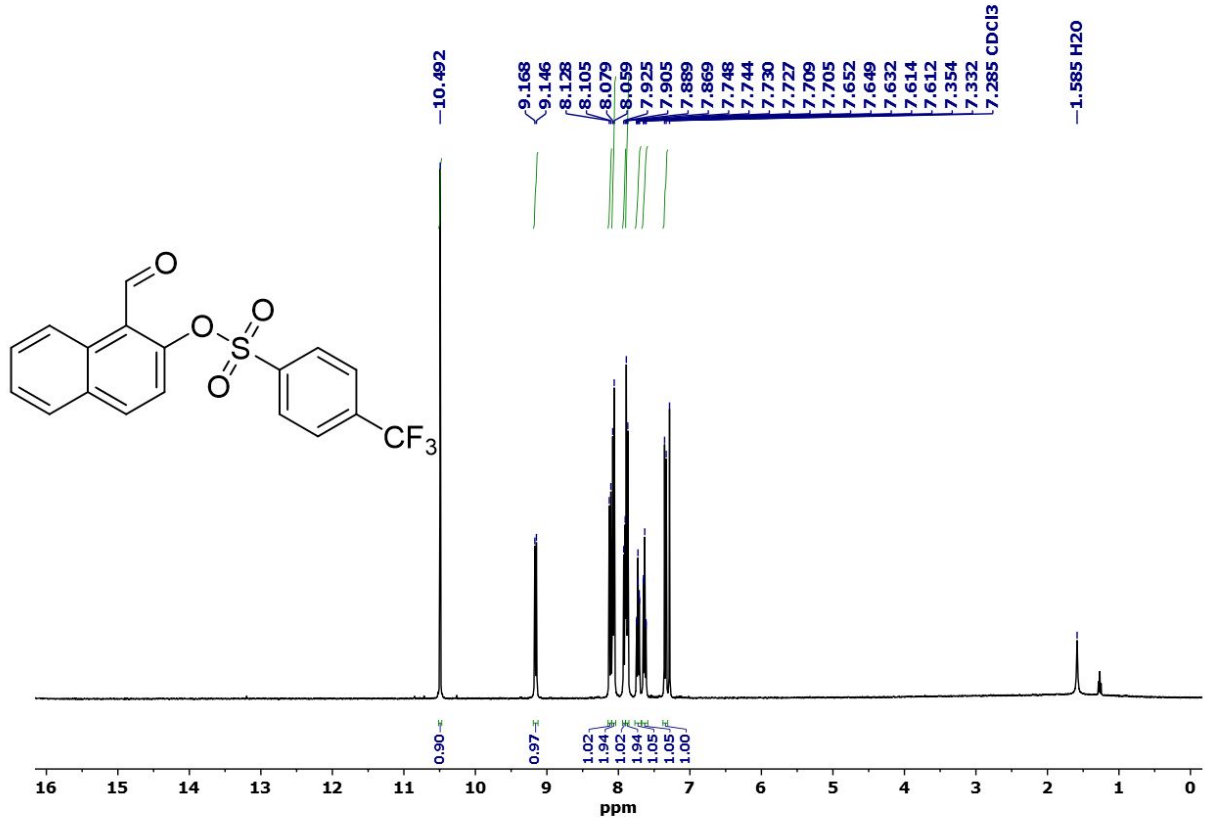

Figure S26. ^{1}H NMR spectrum of compound **9**

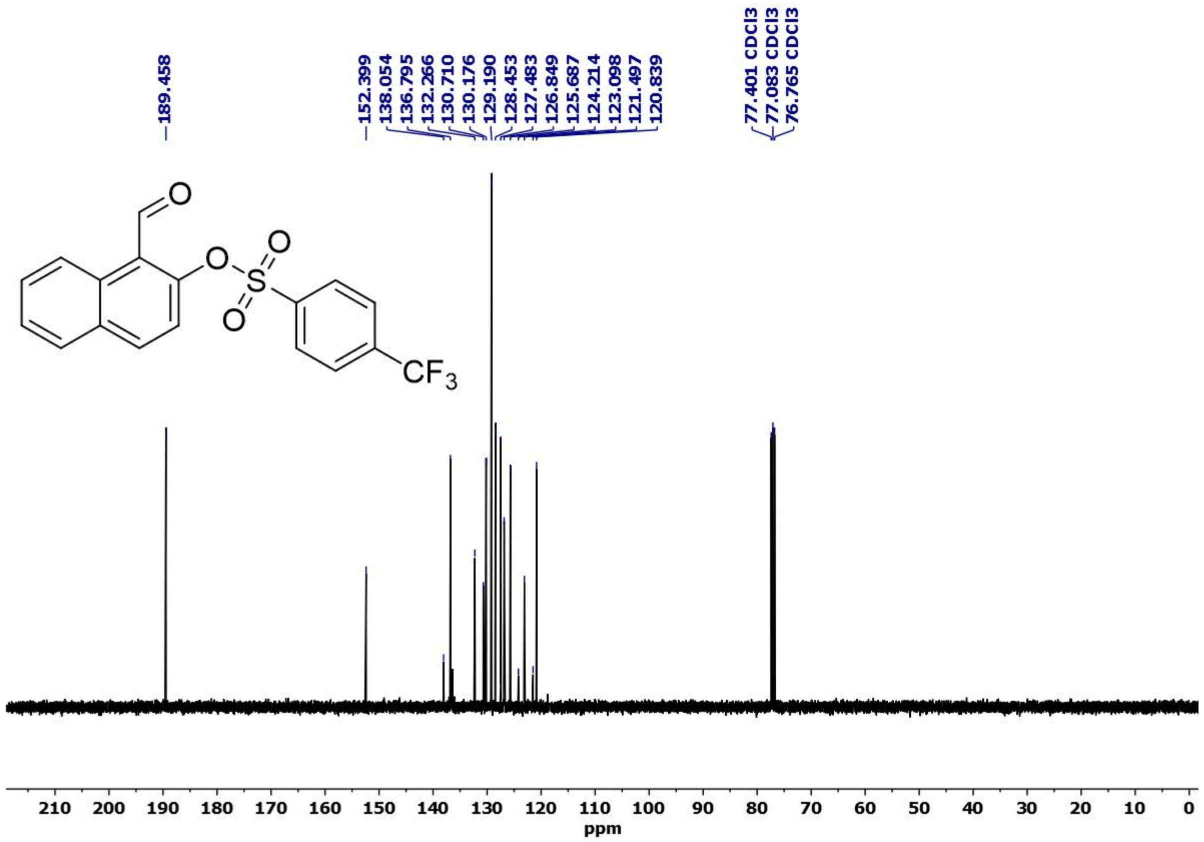

Figure S27. ^{13}C NMR spectrum of compound **9**

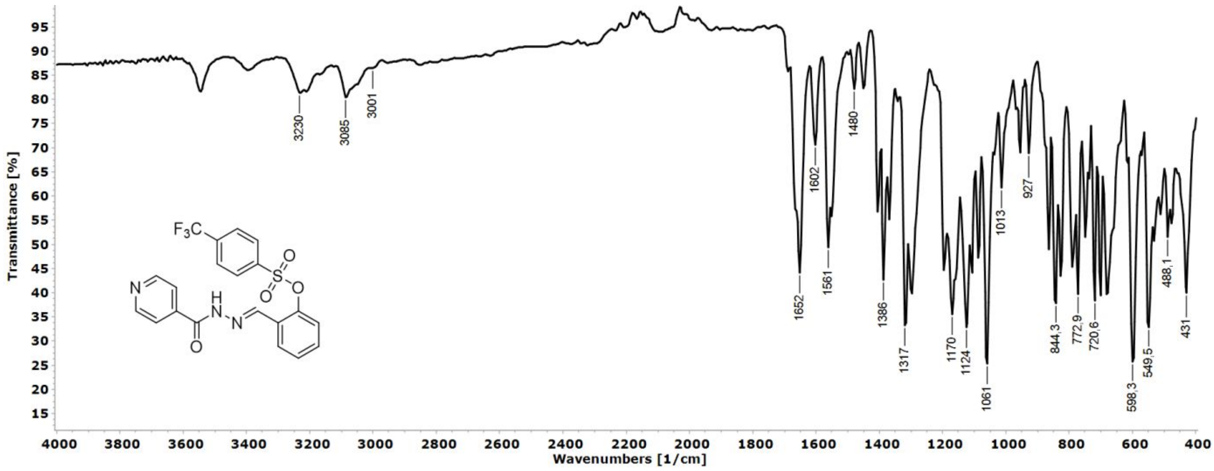

Figure S28. FT-IR spectrum of compound **10**

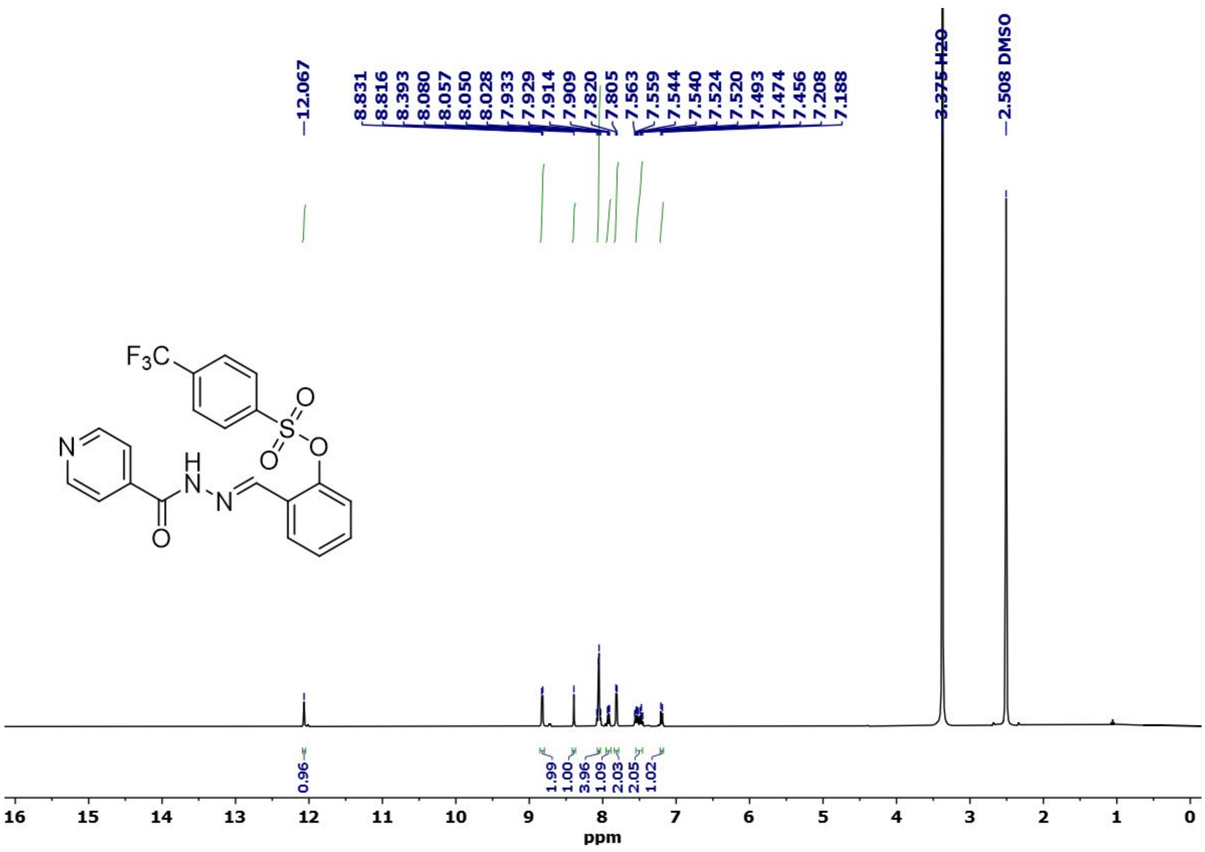

Figure S29. ^{1}H NMR spectrum of compound **10**

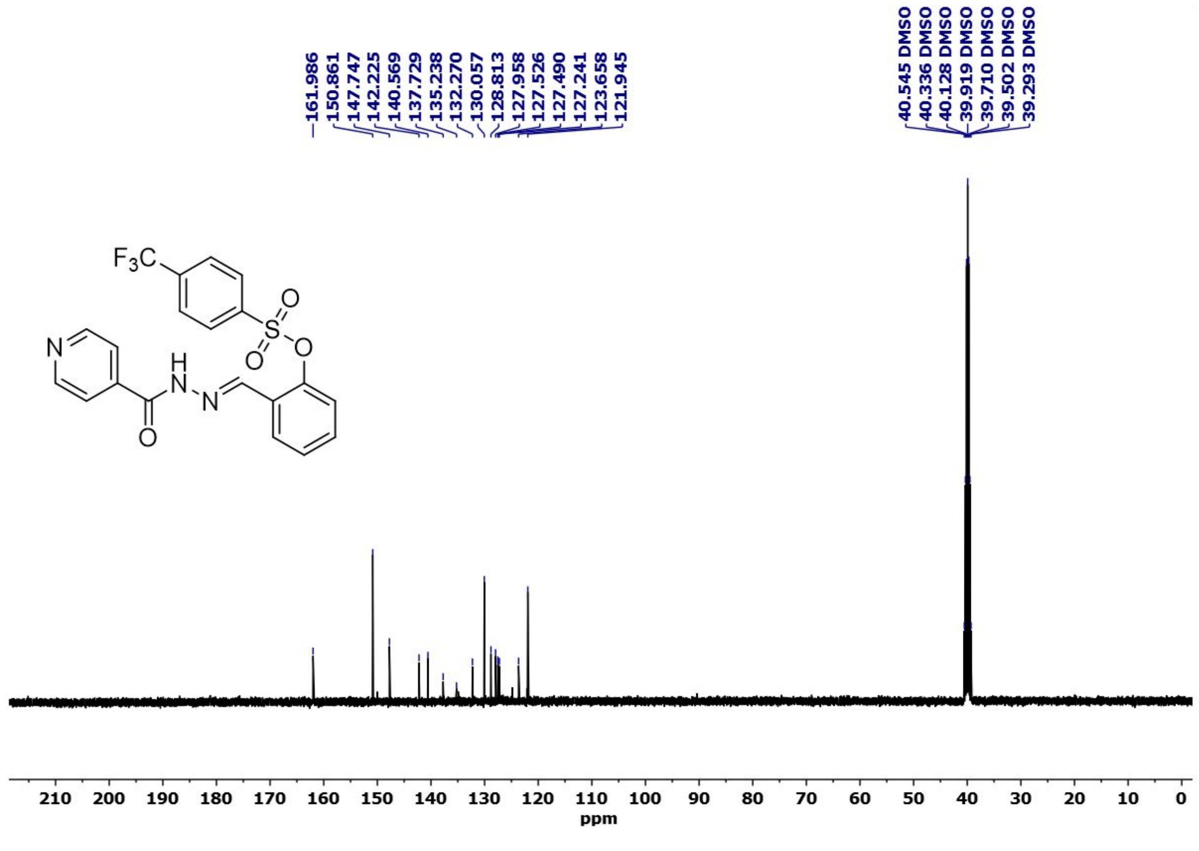

Figure S30. ^{13}C NMR spectrum of compound **10**

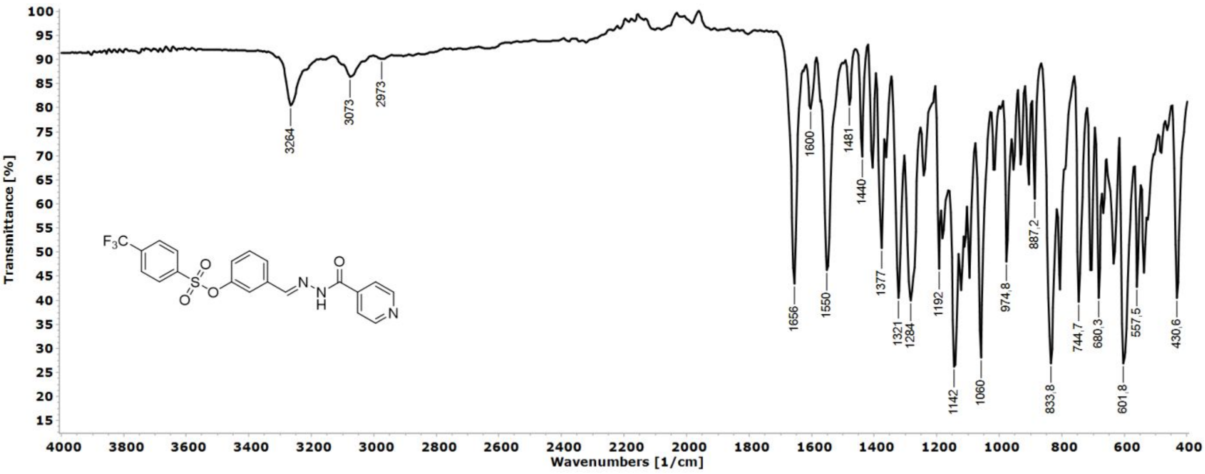

Figure S31. FT-IR spectrum of compound **11**

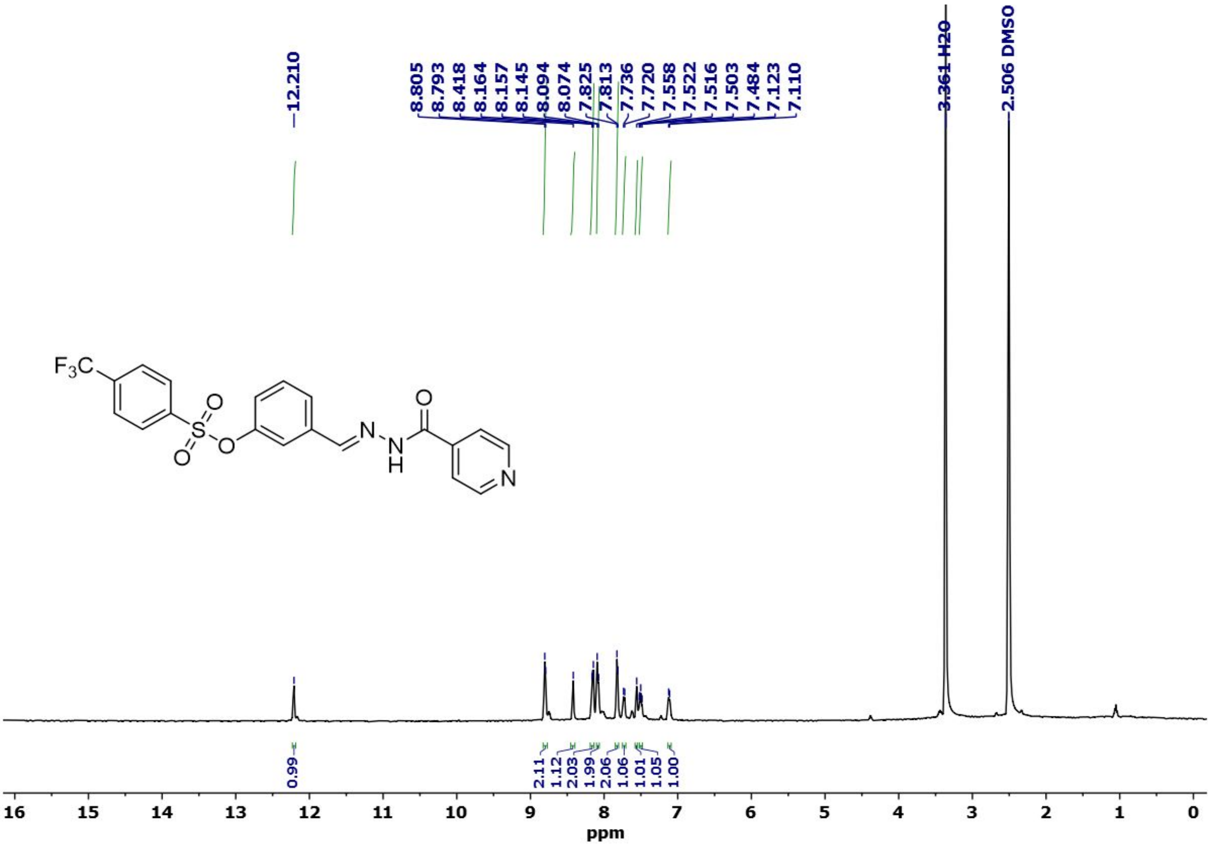

Figure S32. ^{1}H NMR spectrum of compound **11**

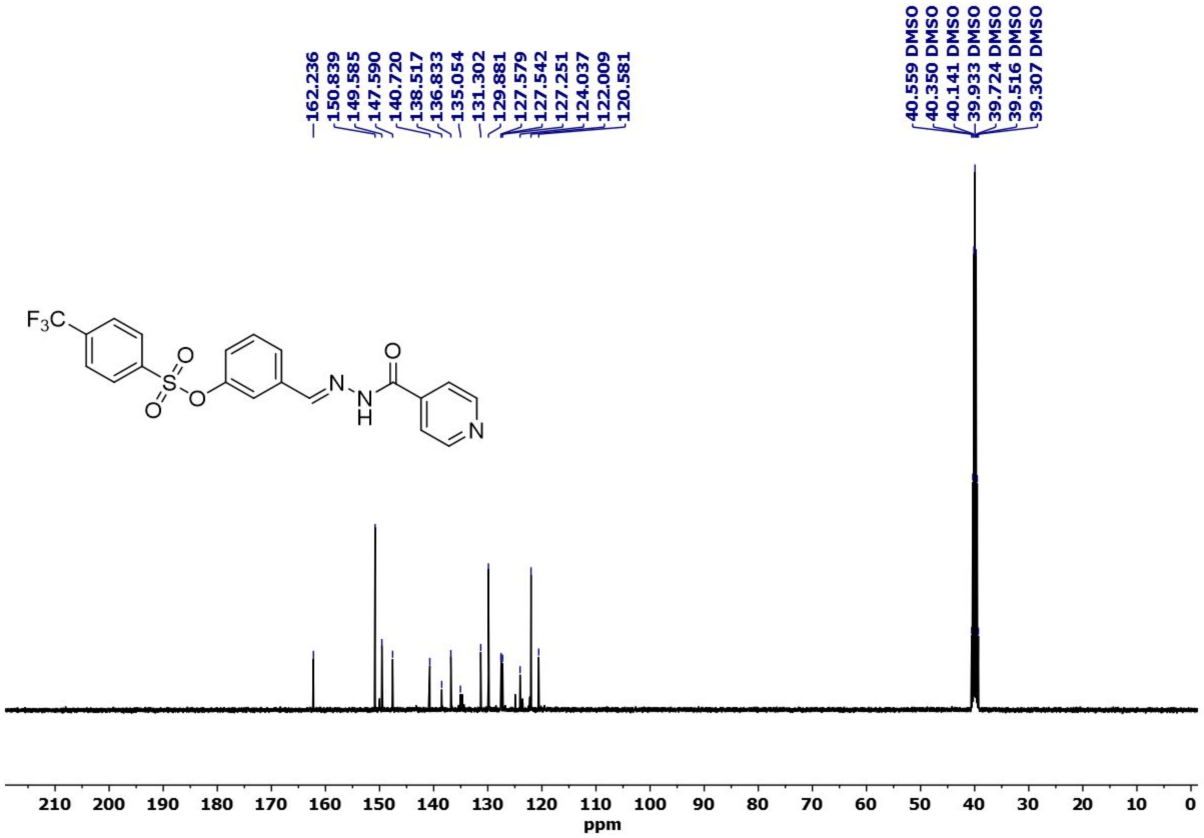

Figure S33. ^{13}C NMR spectrum of compound **11**

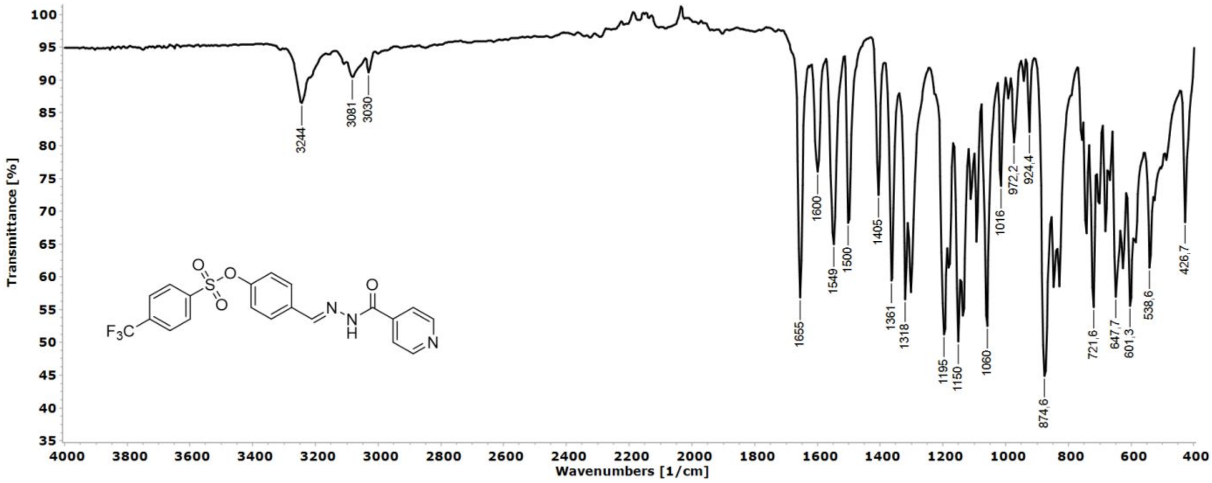

Figure S34. FT-IR spectrum of compound **12**

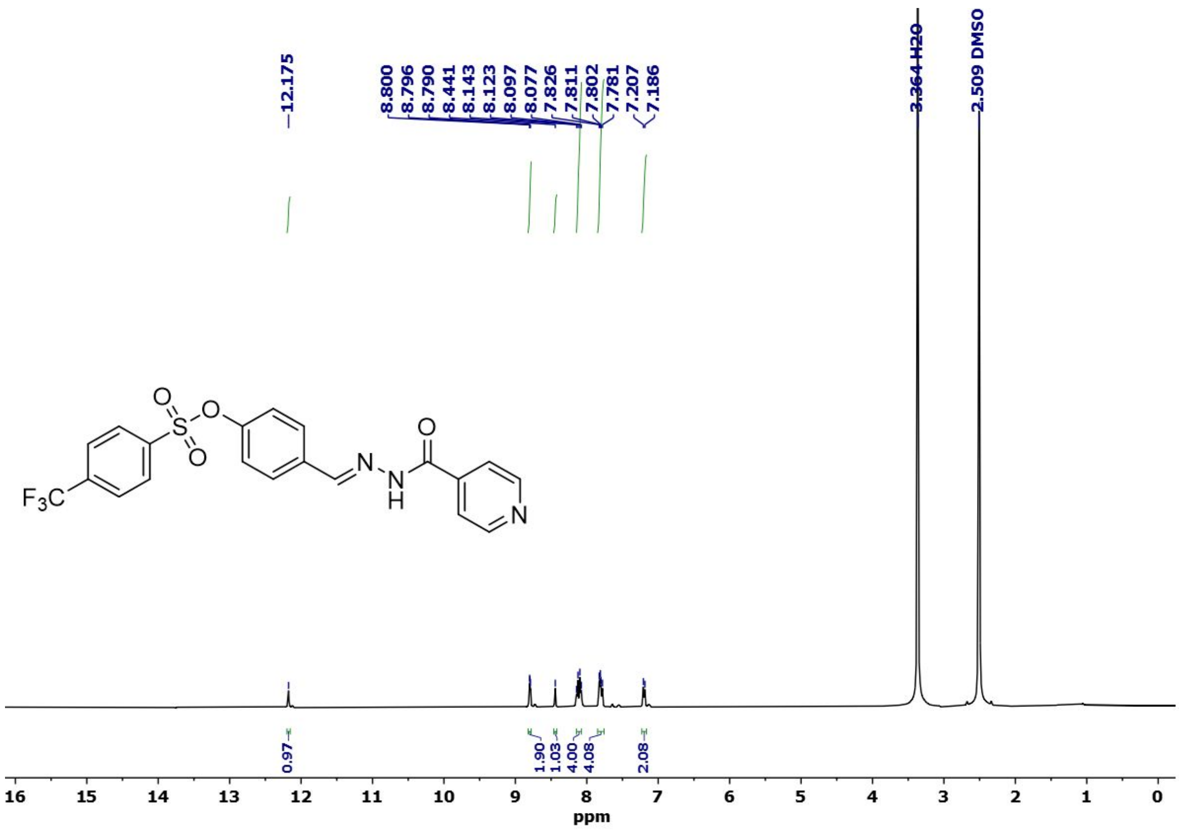

Figure S35. ^{1}H NMR spectrum of compound **12**

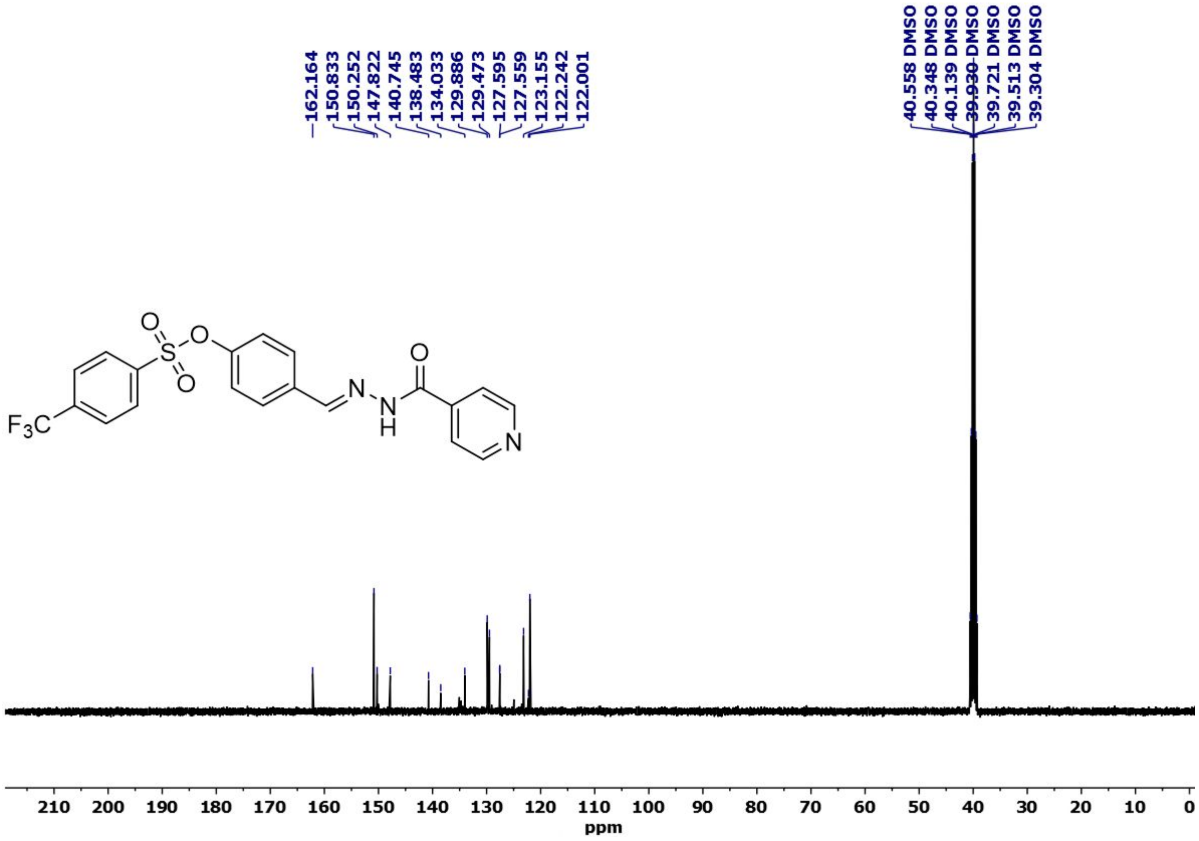

Figure S36. ^{13}C NMR spectrum of compound **12**

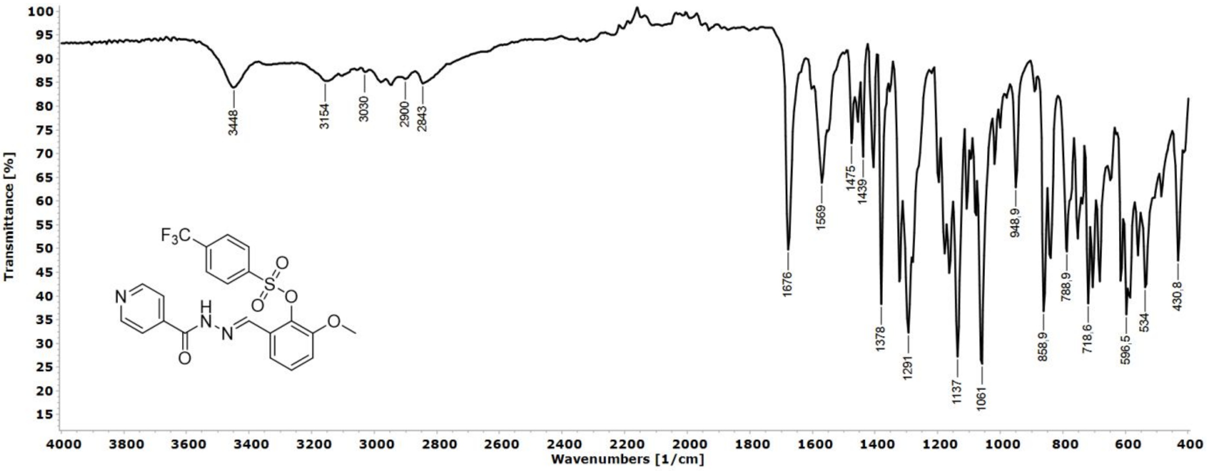

Figure S37. FT-IR spectrum of compound **13**

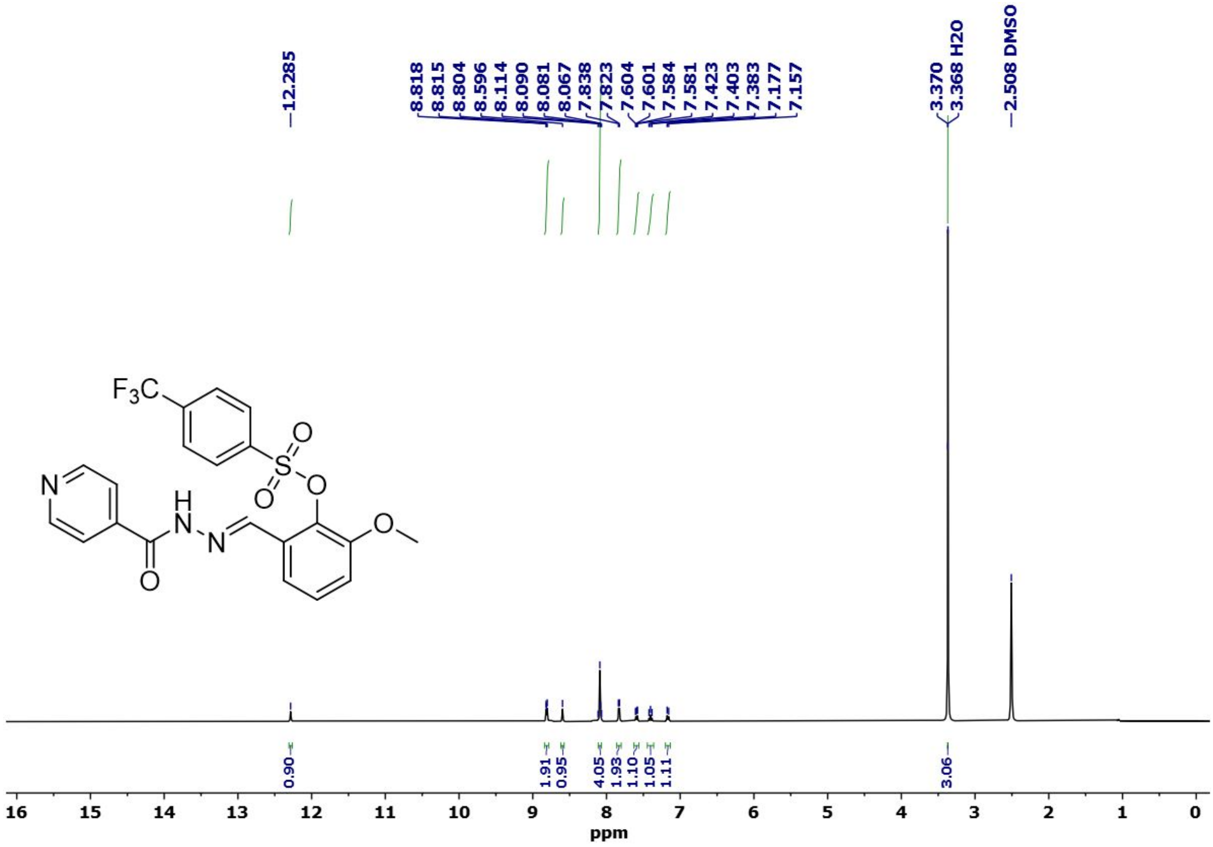

Figure S38. ^{1}H NMR spectrum of compound **13**

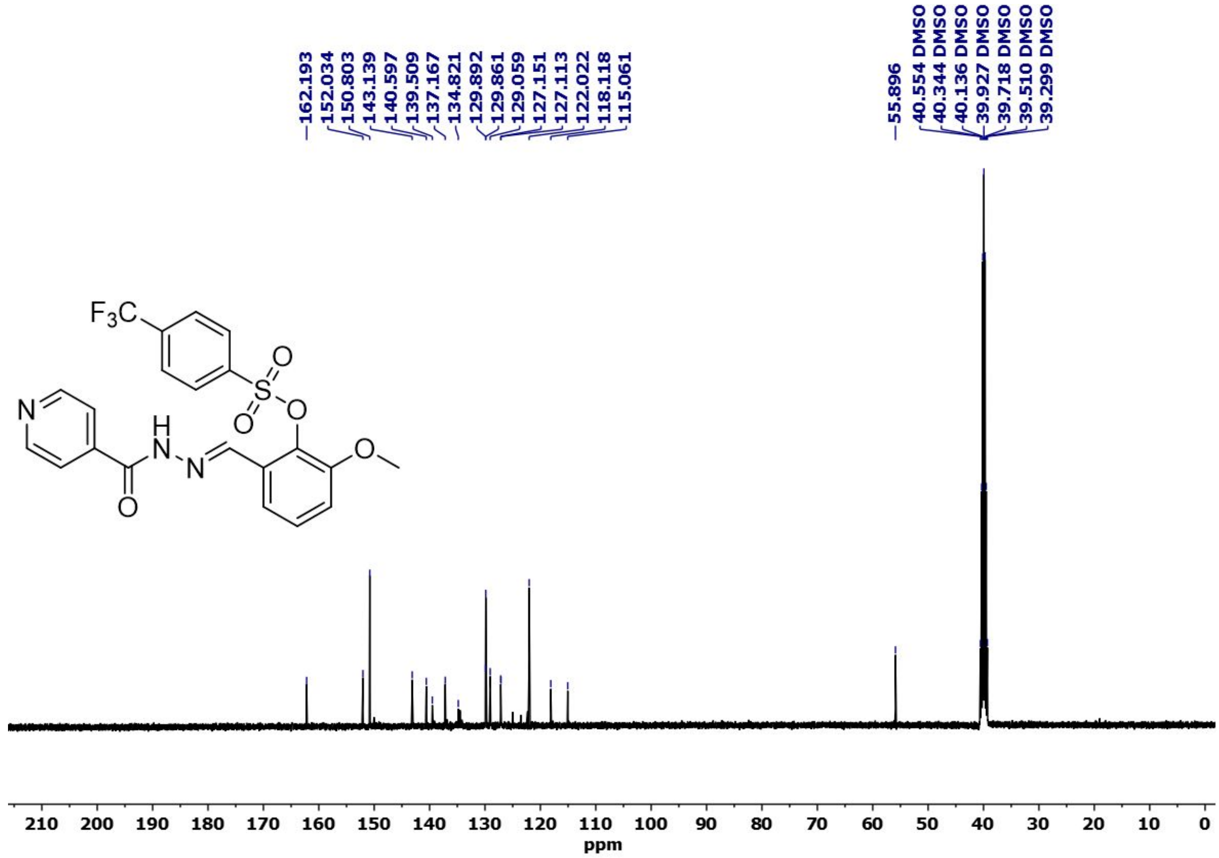

Figure S39. ^{13}C NMR spectrum of compound **13**

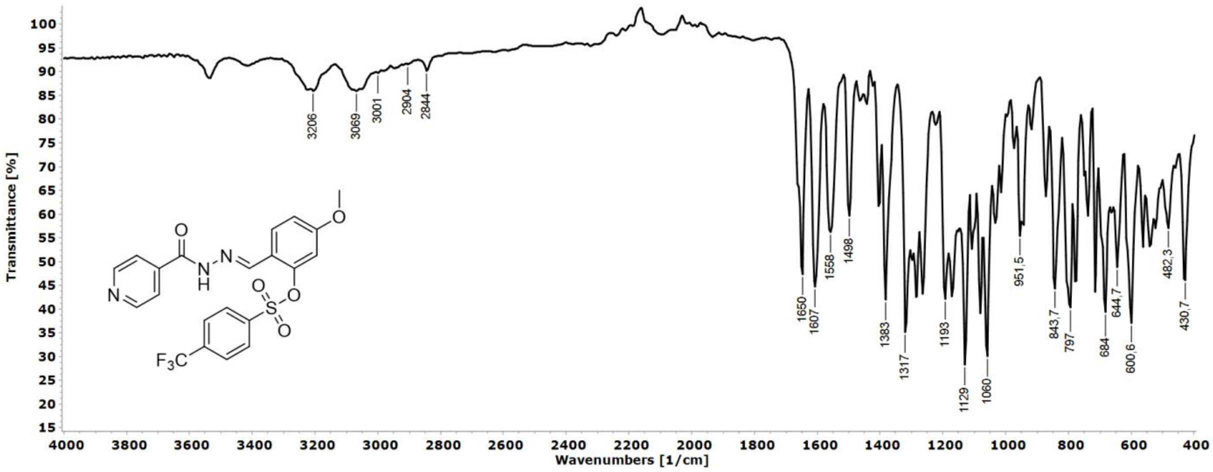

Figure S40. FT-IR spectrum of compound **14**

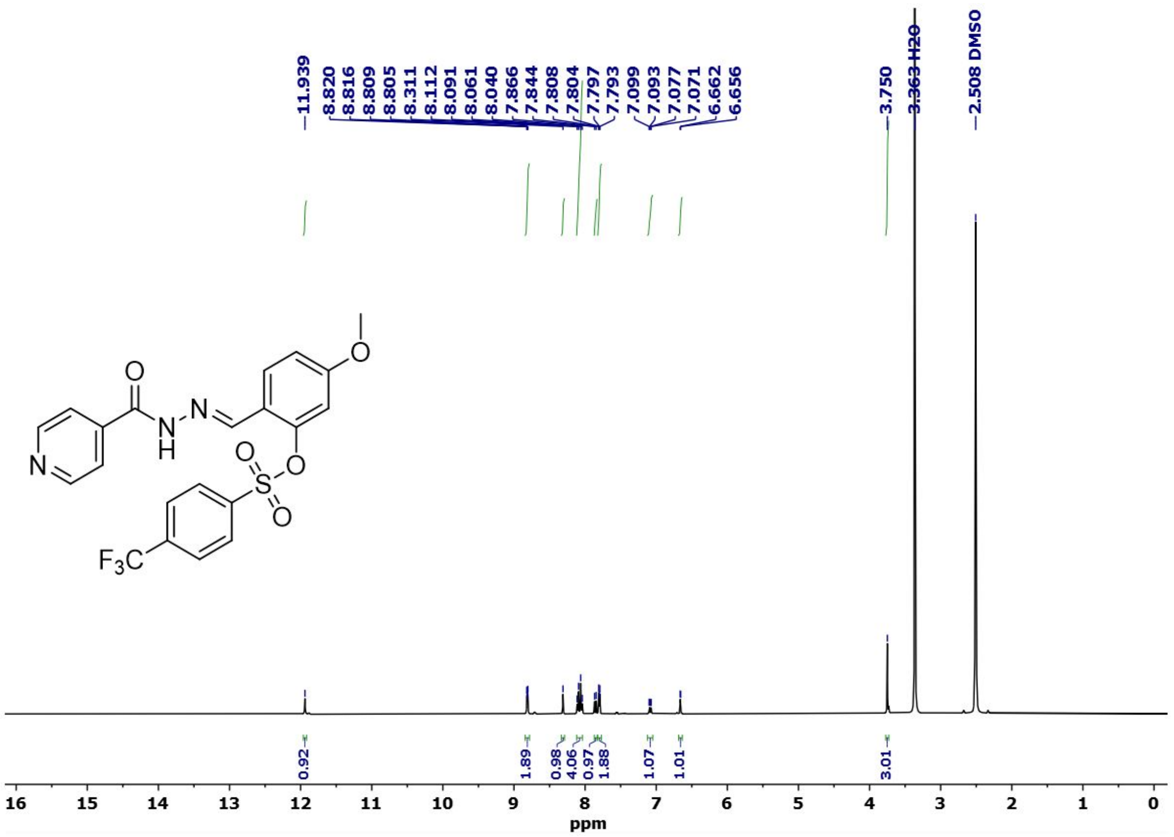

Figure S41. ^{1}H NMR spectrum of compound **14**

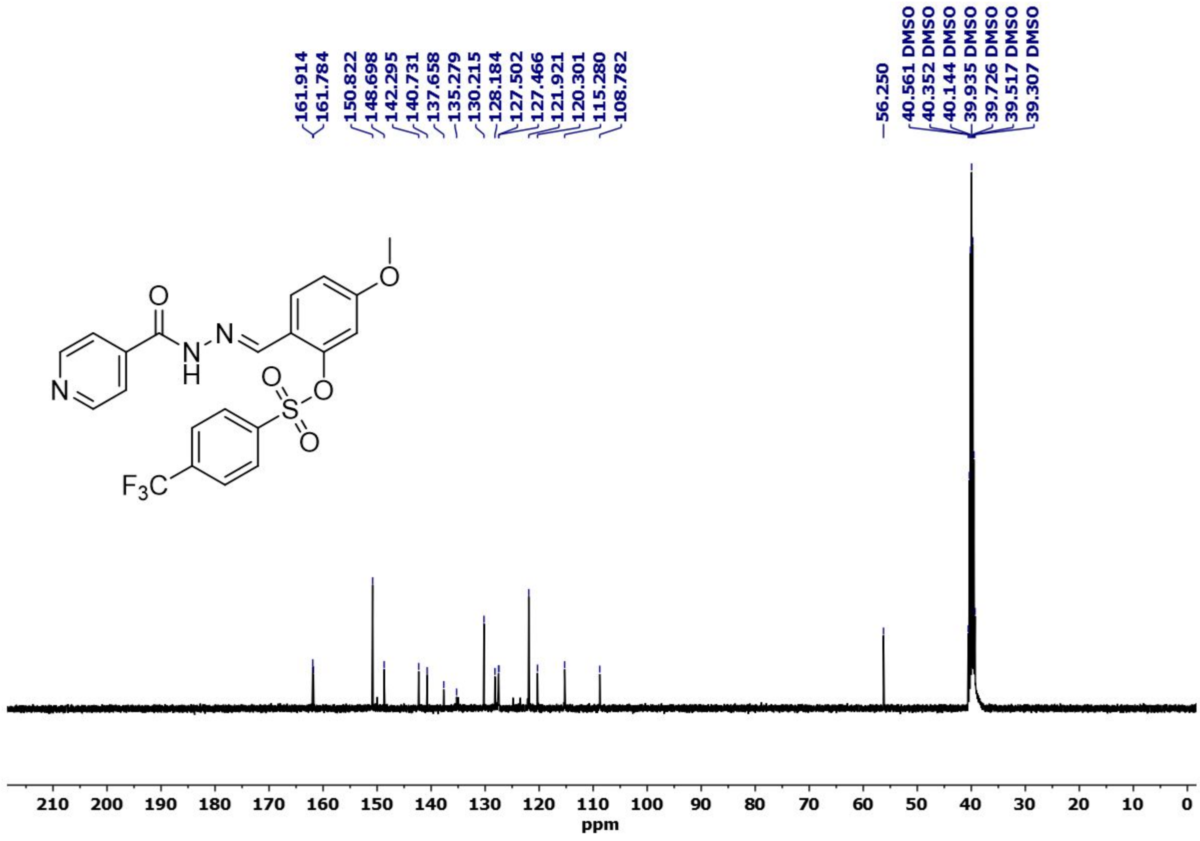

Figure S42. ^{13}C NMR spectrum of compound **14**

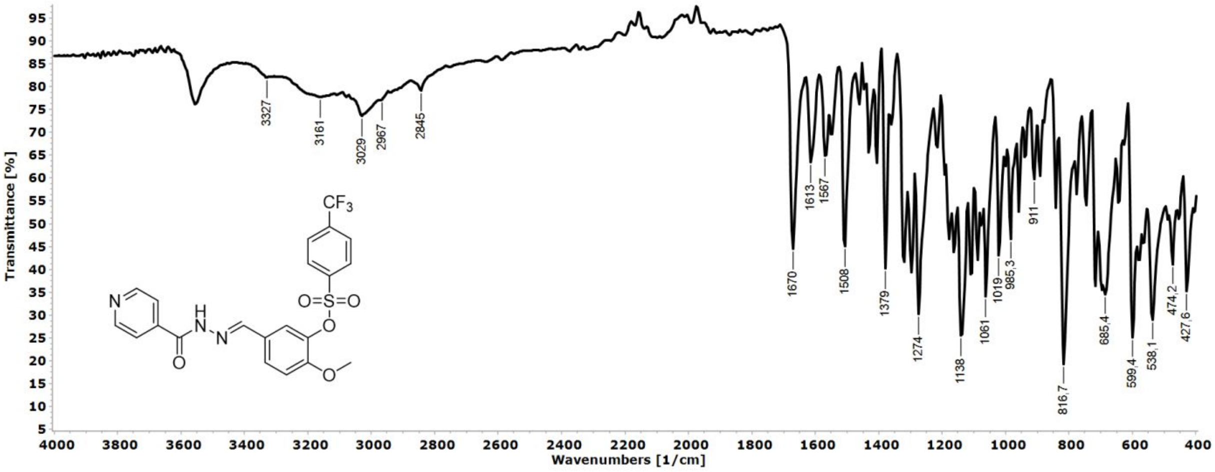

Figure S43. FT-IR spectrum of compound **15**

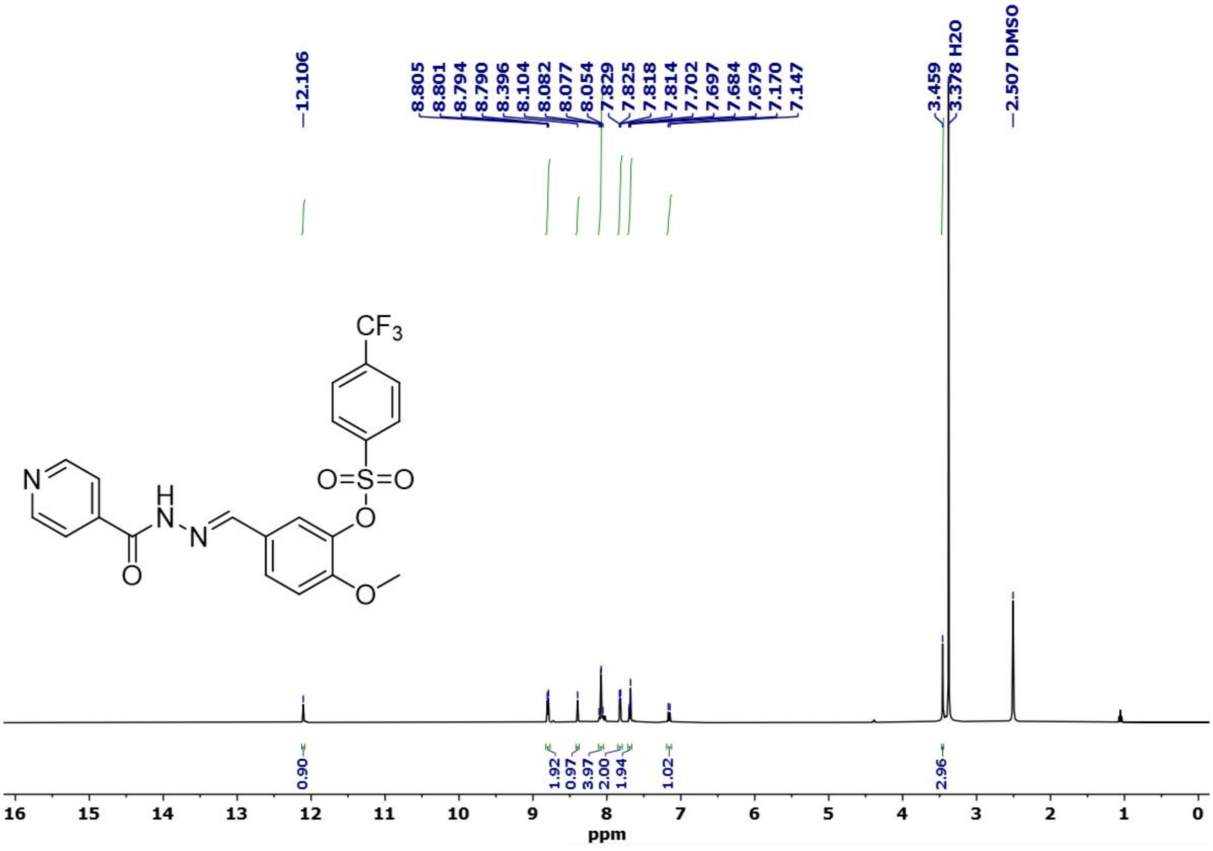

Figure S44. ^{1}H NMR spectrum of compound **15**

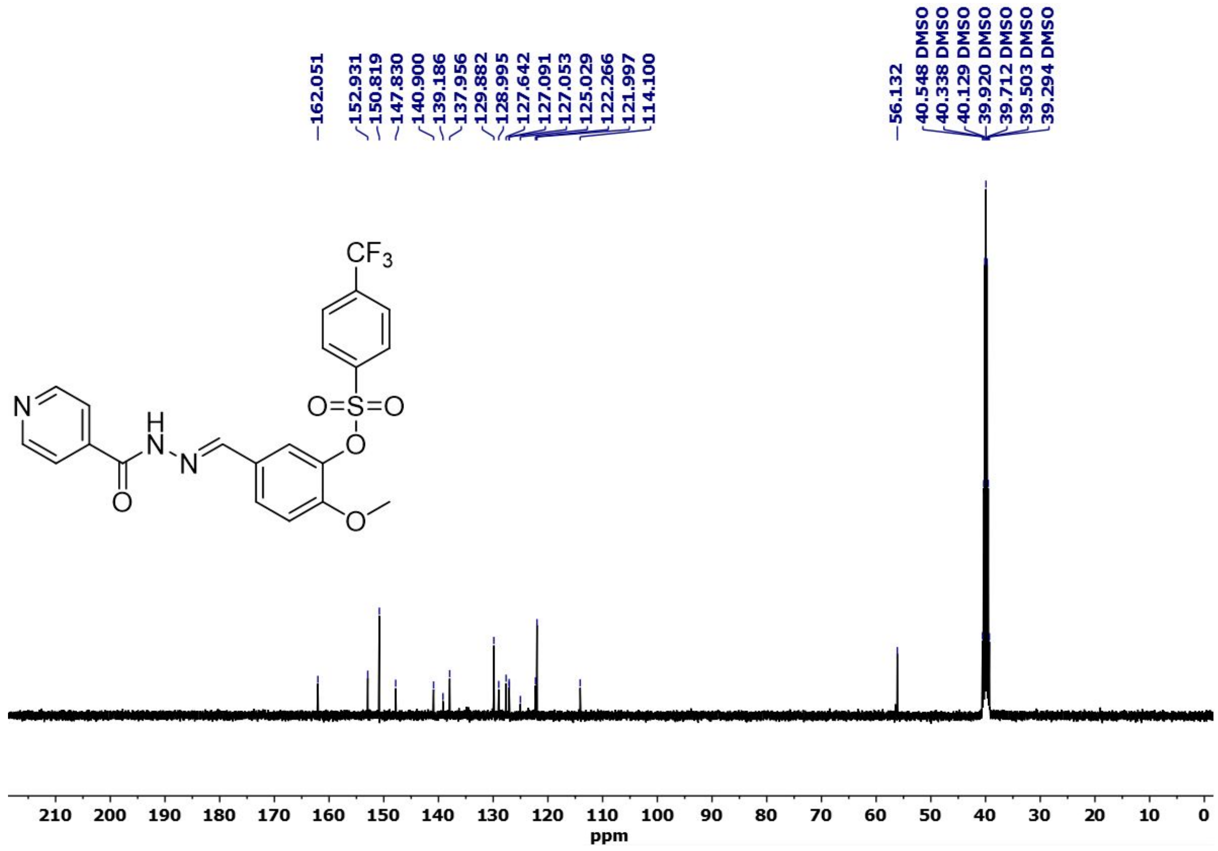

Figure S45. ^{13}C NMR spectrum of compound **15**

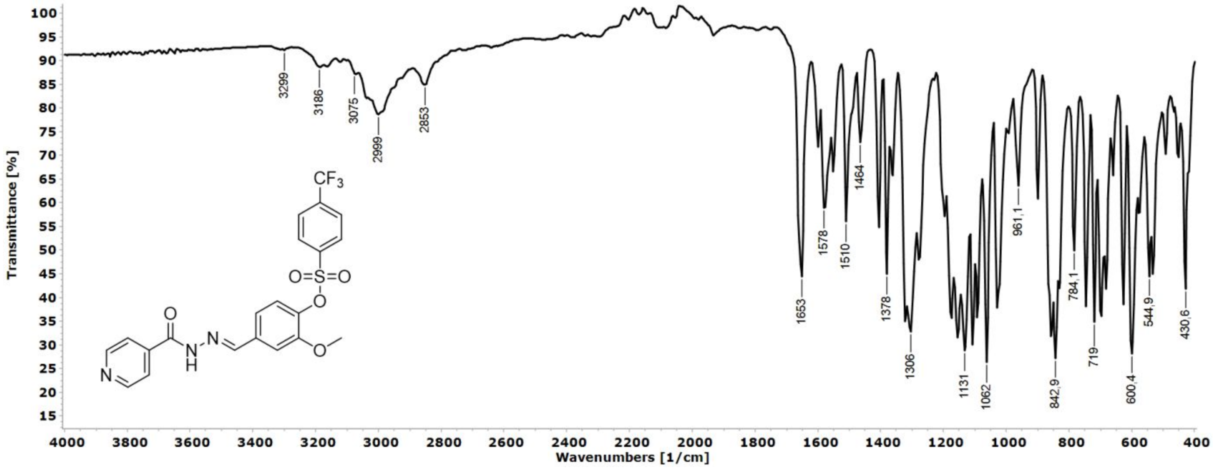

Figure S46. FT-IR spectrum of compound **16**

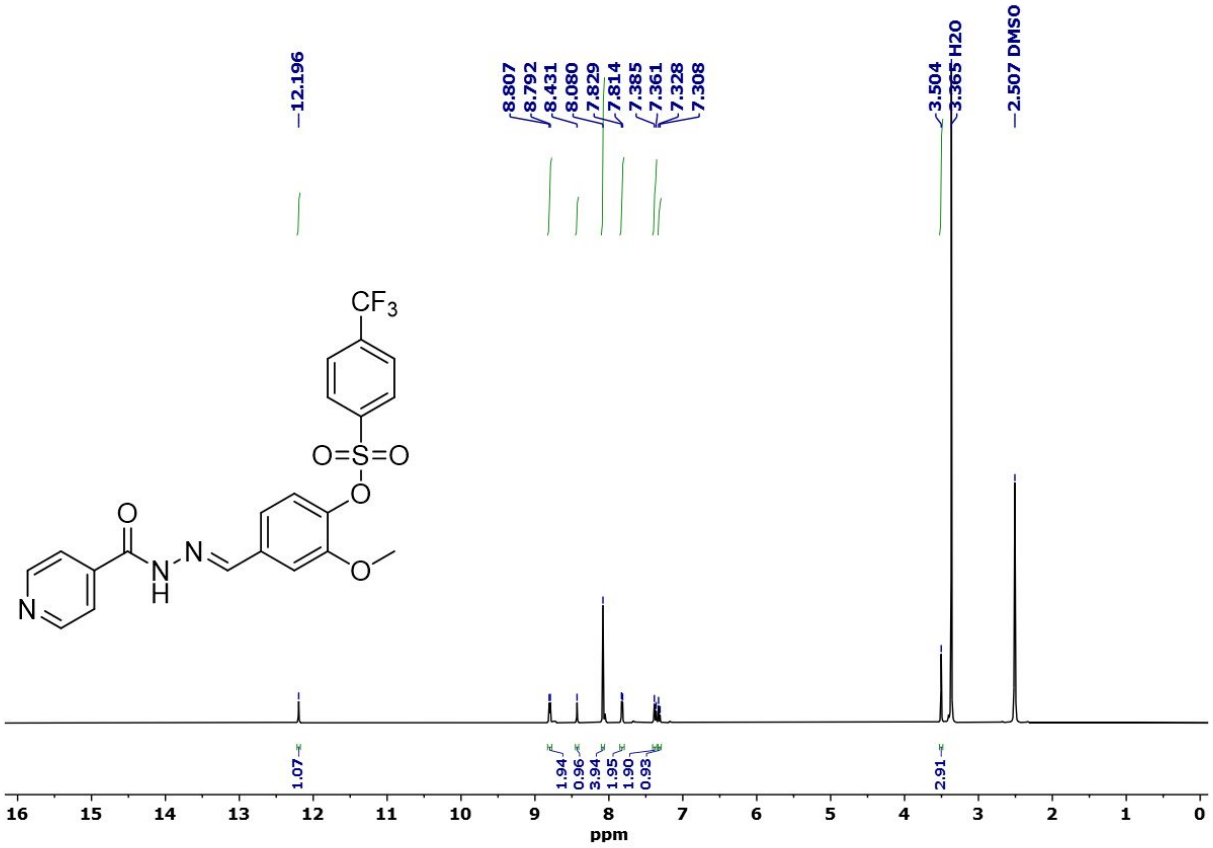

Figure S47. ^{1}H NMR spectrum of compound **16**

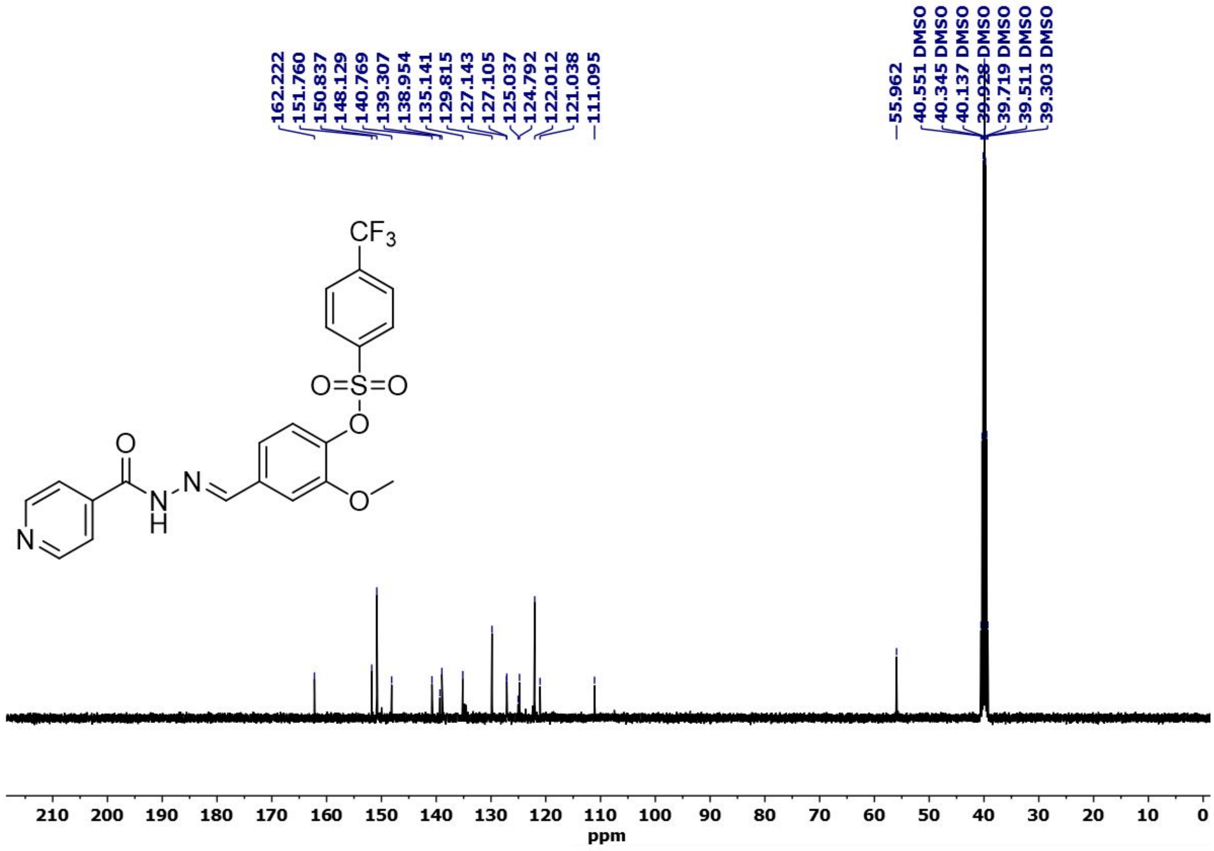

Figure S48. ^{13}C NMR spectrum of compound **16**

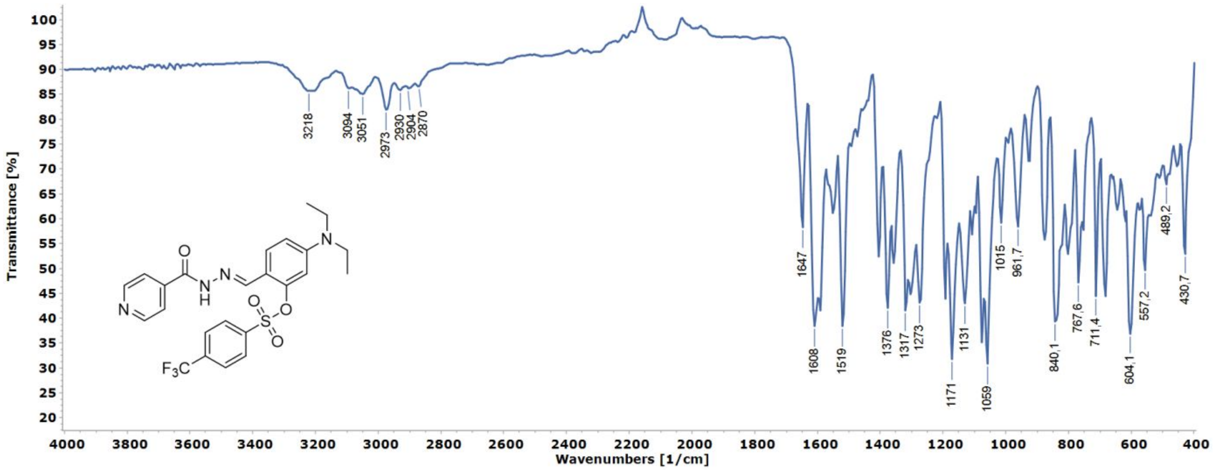

Figure S49. FT-IR spectrum of compound **17**

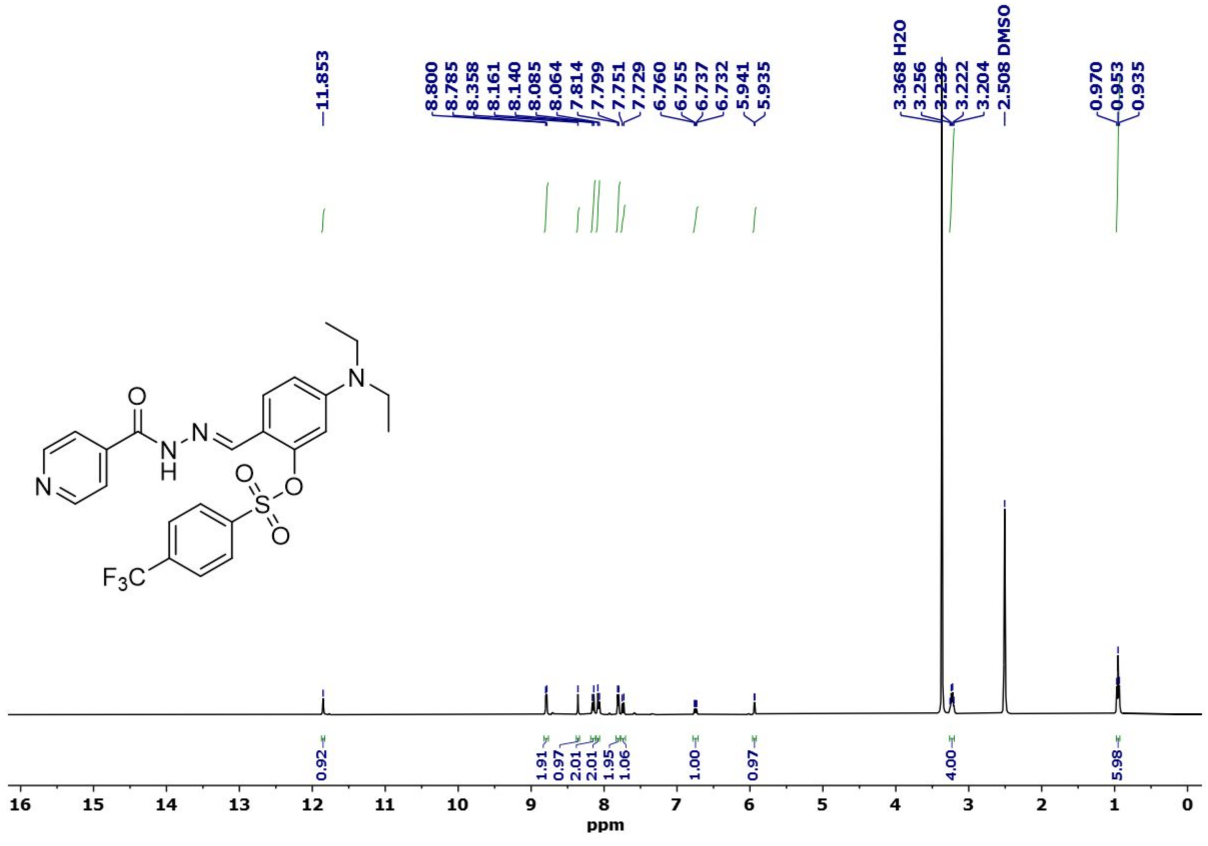

Figure S50. ^{1}H NMR spectrum of compound **17**

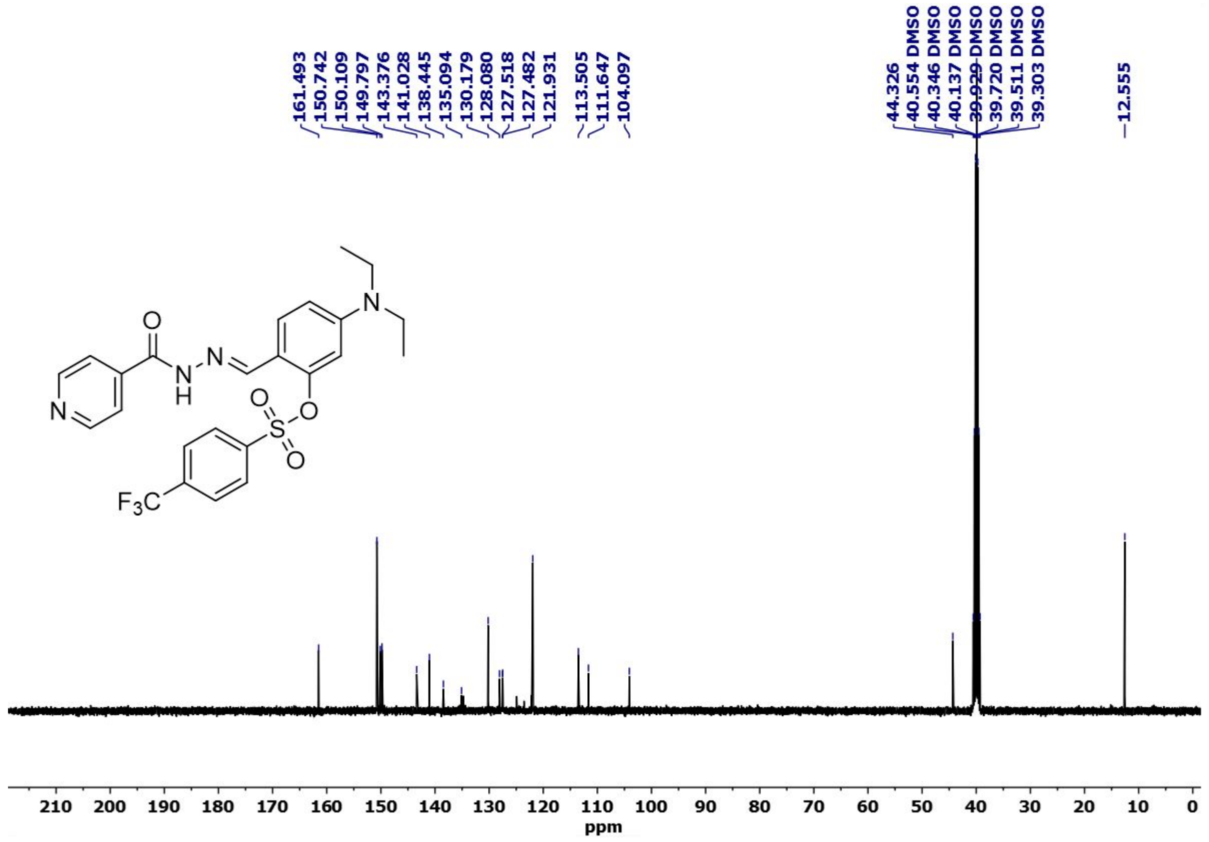

Figure S51. ^{13}C NMR spectrum of compound **17**

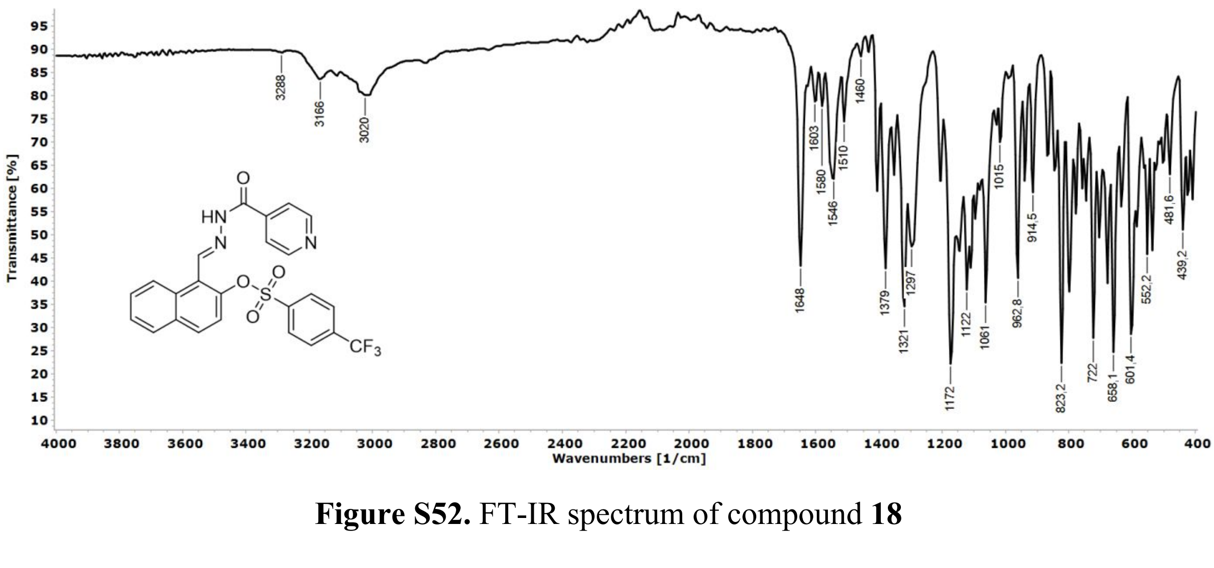

Figure S52. FT-IR spectrum of compound **18**

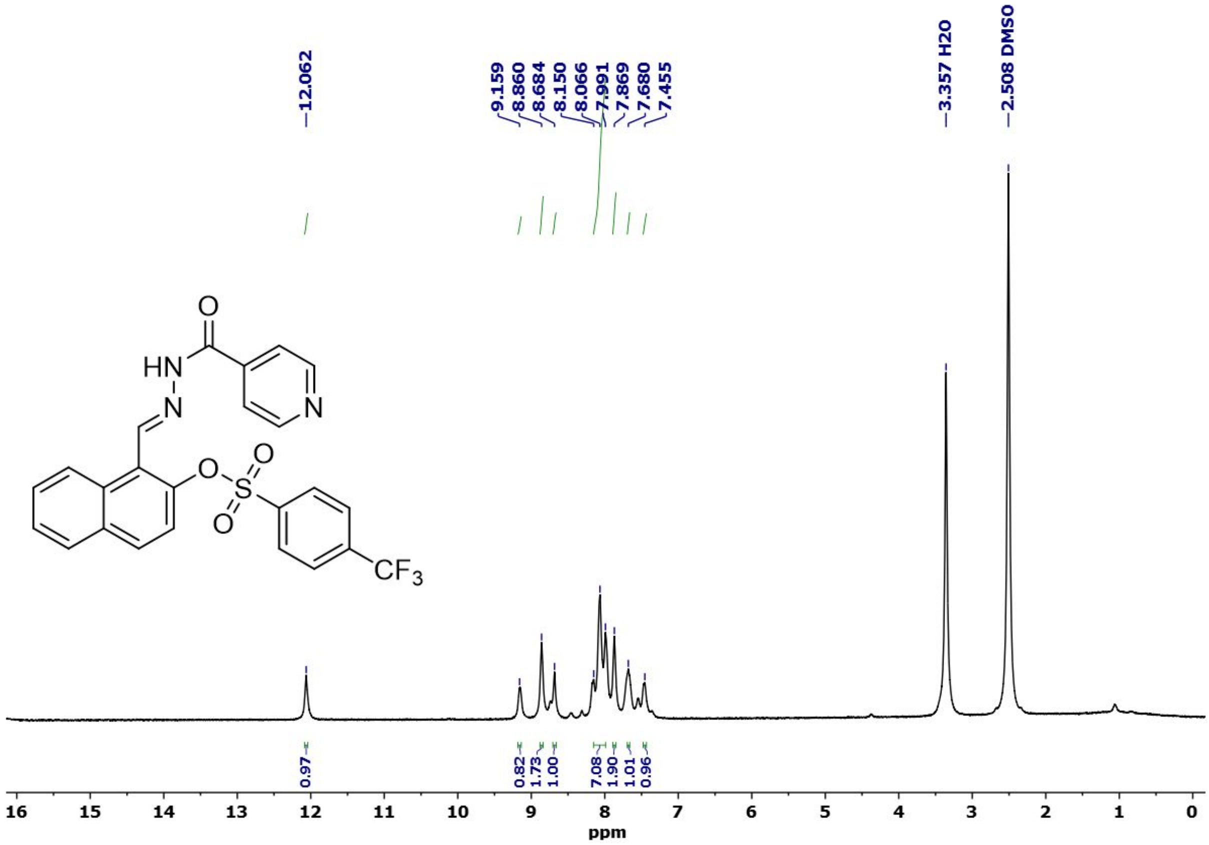

Figure S53. ^{1}H NMR spectrum of compound **18**

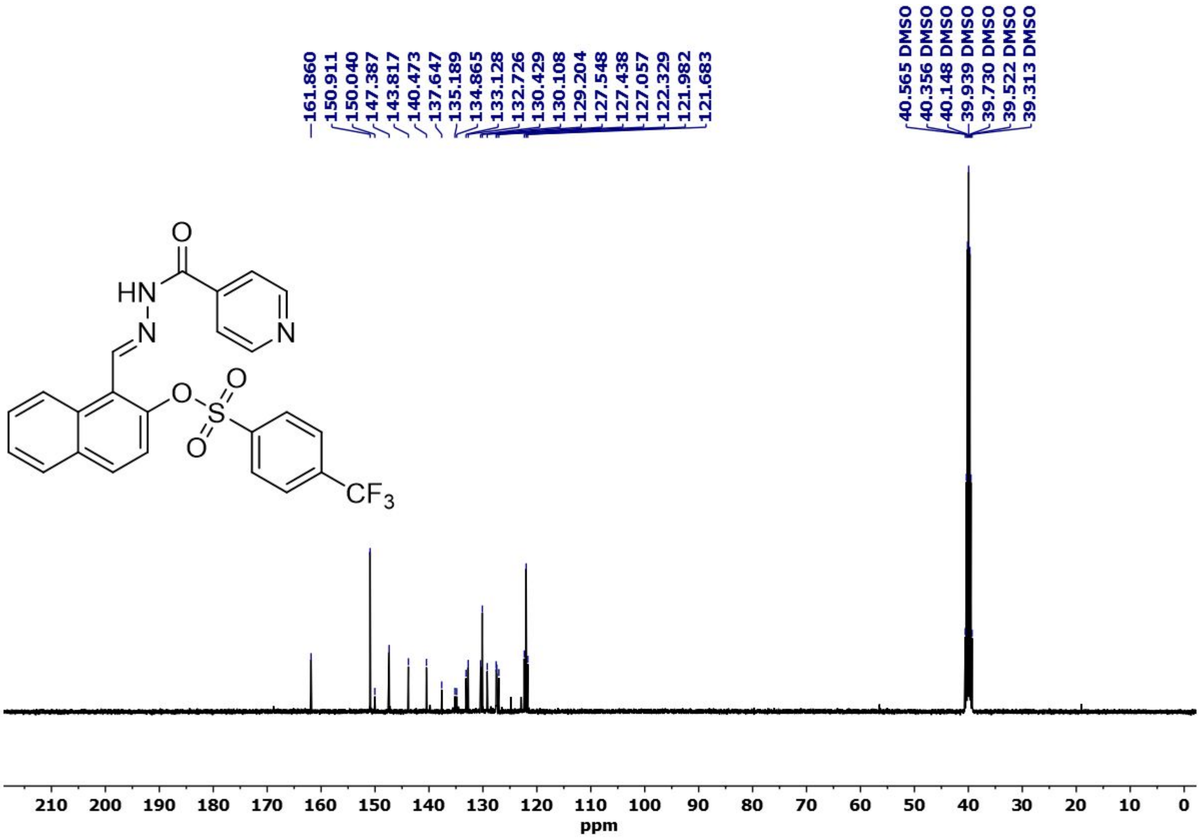

Figure S54. ^{13}C NMR spectrum of compound **18**

3. Molecular Docking Studies

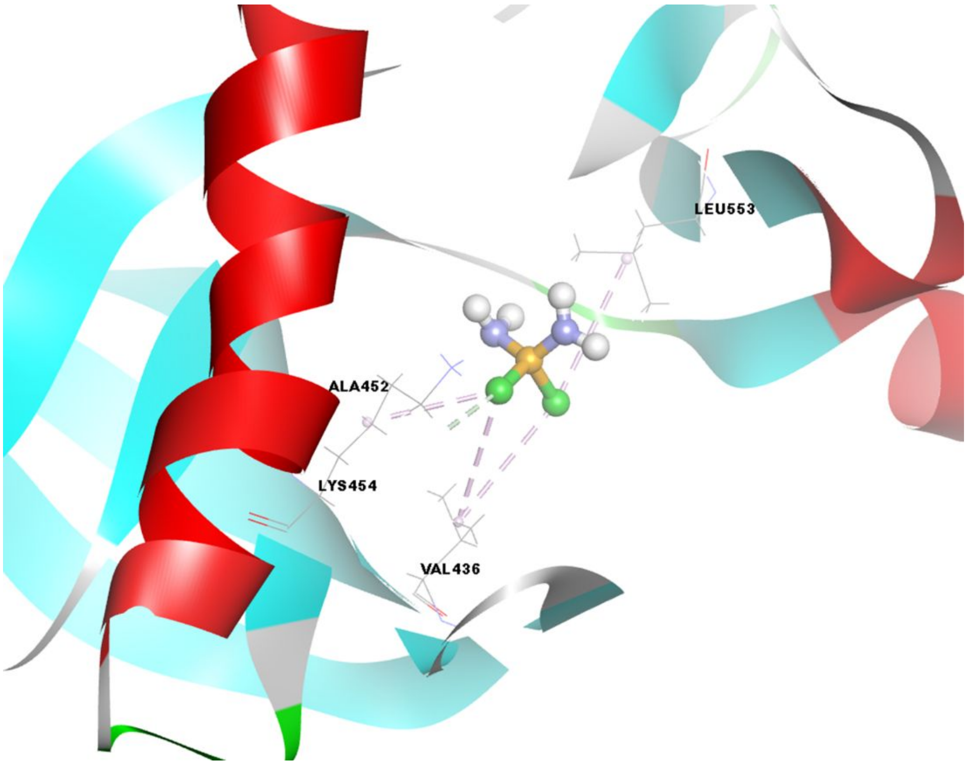

Figure S55. 3D image of cisplatin, default and orange color, ball and stick form against PC3-FAK.

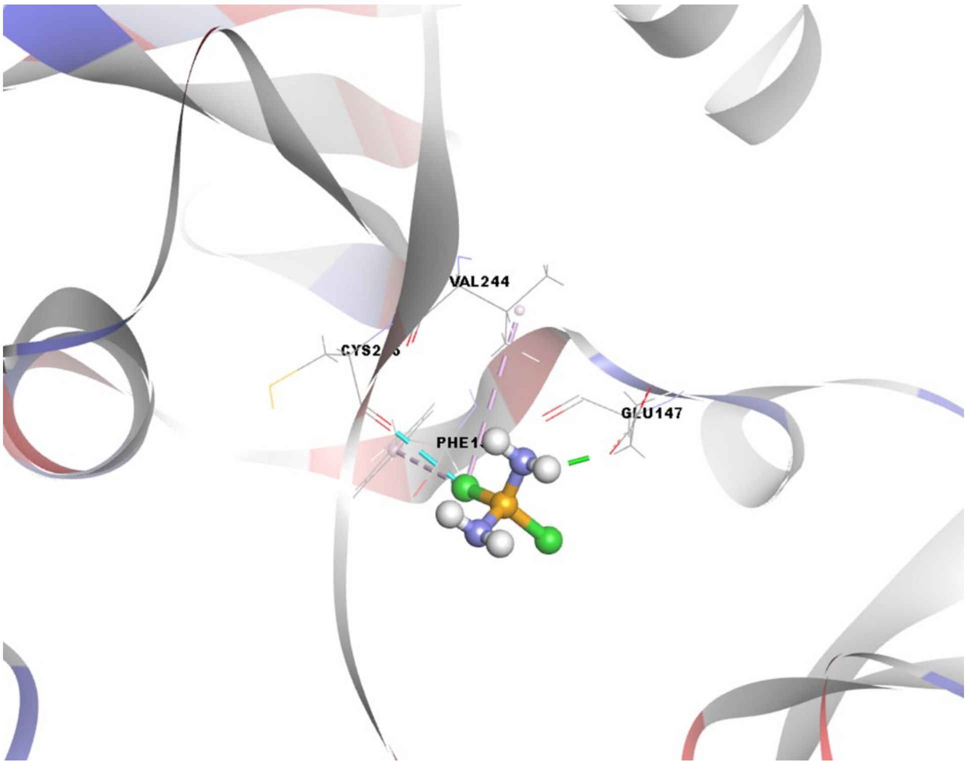

Figure S56. 3D image of cisplatin, default and orange color, ball and stick form against PC3-Src.

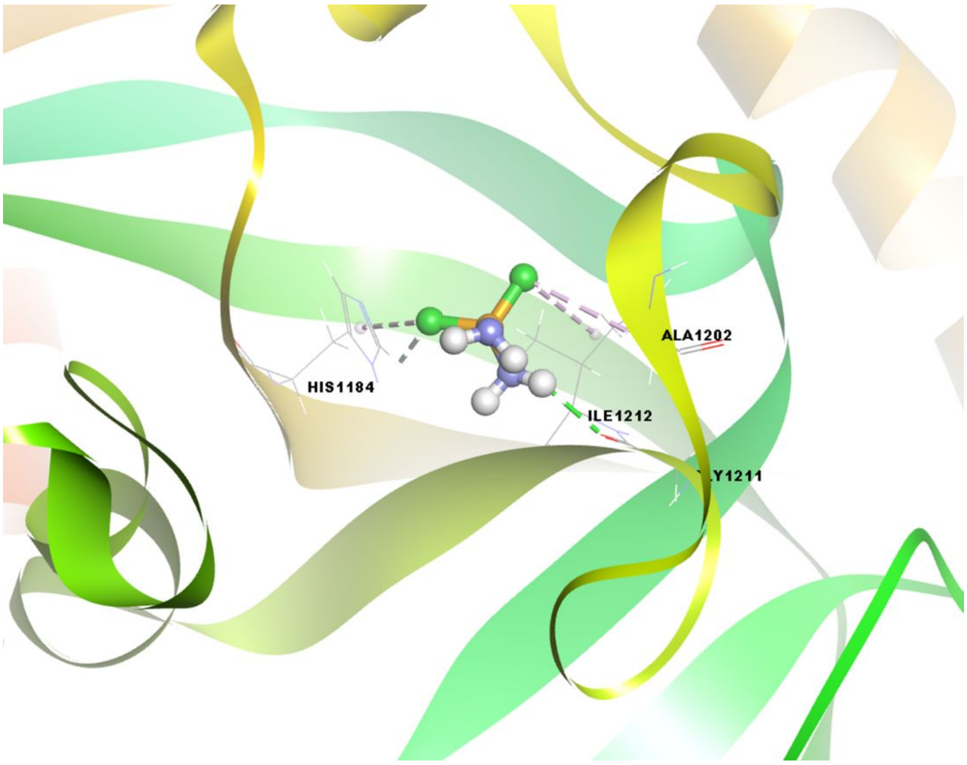

Figure S57. 3D image of cisplatin, default and orange color, ball and stick form against DLD-1-TKNS.

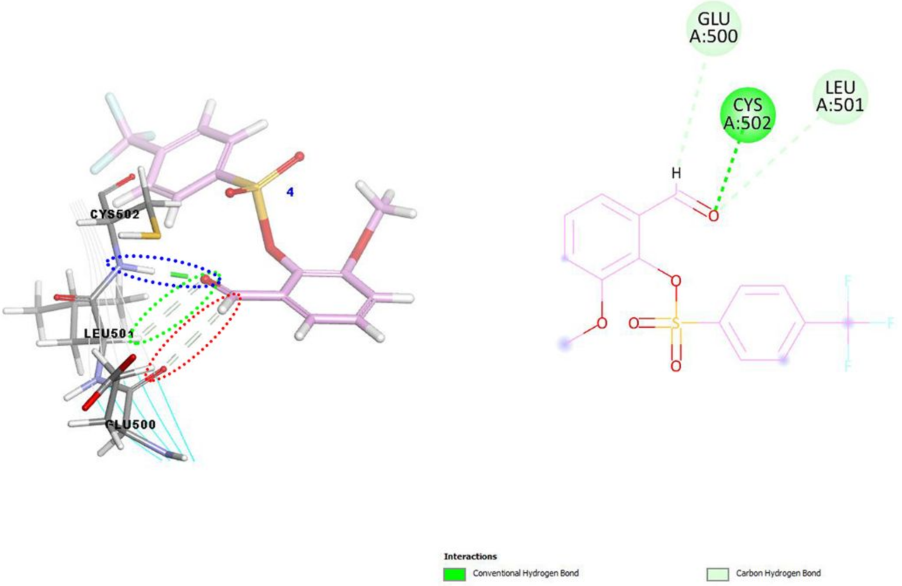

Figure S58. Interaction details of the formyl group on compound 4 with residues of PC3-FAK.

Table S1. The stationary orientations of compound **1** using the DFT/B3LYP/LANL2DZ level by using Gaussian 09.

Standard orientation:

Center Number	Atomic Number	Atomic Type	Coordinates (Angstroms) X	Y	Z
1	6	0	-2.052493	-0.244059	-1.189768
2	6	0	-2.973280	-0.018479	-0.149487
3	6	0	-2.666658	0.835639	0.927749
4	6	0	-1.415886	1.471804	0.975820
5	6	0	-0.520441	1.225851	-0.071749
6	6	0	-0.797601	0.386723	-1.156665
7	16	0	1.136593	2.080123	-0.019505
8	8	0	2.169292	0.868895	1.082904
9	8	0	1.777166	2.064154	-1.506409
10	8	0	1.024419	3.413644	0.877695
11	6	0	2.927022	-0.095644	0.421372
12	6	0	4.105495	0.280504	-0.256502
13	6	0	4.897880	-0.707715	-0.861913
14	6	0	4.519244	-2.066905	-0.795875
15	6	0	3.343501	-2.437370	-0.123566
16	6	0	2.525047	-1.460295	0.483707
17	6	0	1.293857	-1.865209	1.208369
18	8	0	0.864016	-3.040079	1.232451
19	6	0	-4.334138	-0.658548	-0.216822
20	9	0	-4.869850	-0.912906	1.051430
21	9	0	-5.273069	0.160129	-0.878547
22	9	0	-4.322616	-1.873271	-0.911245
23	1	0	-2.304013	-0.908824	-2.009636
24	1	0	-3.388108	0.996155	1.722391
25	1	0	-1.153726	2.145340	1.785825
26	1	0	-0.072803	0.246189	-1.952430
27	1	0	4.382087	1.328974	-0.299981
28	1	0	5.807330	-0.421966	-1.383442

29	1	0	5.140785	-2.825558	-1.263535
30	1	0	3.036748	-3.477369	-0.050421
31	1	0	0.767585	-1.061176	1.747397

Table S2. The stationary orientations of compound **2** using the DFT/B3LYP/LANL2DZ level by using Gaussian 09.

Standard orientation:

Center Number	Atomic Number	Atomic Type	Coordinates (Angstroms) X	Y	Z
1	6	0	-2.137632	0.366311	1.139620
2	6	0	-3.081561	0.230841	0.103867
3	6	0	-2.900027	-0.702797	-0.935079
4	6	0	-1.752976	-1.512698	-0.948459
5	6	0	-0.828076	-1.348886	0.089092
6	6	0	-0.984909	-0.436809	1.138694
7	16	0	0.692885	-2.429678	0.082915
8	8	0	1.859606	-1.473503	-1.106981
9	8	0	1.348187	-2.393993	1.564028
10	8	0	0.375731	-3.796458	-0.709406
11	6	0	2.670803	-0.491766	-0.533654
12	6	0	3.807088	-0.853852	0.223111
13	6	0	4.653740	0.153776	0.722057
14	6	0	4.372032	1.506114	0.470163
15	6	0	3.229243	1.863715	-0.286590
16	6	0	2.368786	0.863192	-0.779153
17	6	0	2.922840	3.289353	-0.568979
18	8	0	3.609462	4.250233	-0.165578
19	6	0	-4.288686	1.129355	0.087353
20	9	0	-5.384666	0.545803	-0.559005
21	9	0	-4.711371	1.484302	1.374323
22	9	0	-4.038249	2.342336	-0.587810
23	1	0	-2.302929	1.077139	1.942872

24	1	0	-3.647114	-0.805519	-1.715403
25	1	0	-1.592413	-2.254045	-1.725082
26	1	0	-0.248367	-0.373063	1.933571
27	1	0	4.012803	-1.903839	0.406210
28	1	0	5.529372	-0.121023	1.303101
29	1	0	5.016487	2.297533	0.843043
30	1	0	2.015449	3.463892	-1.180705
31	1	0	1.491239	1.116470	-1.370574

Table S3. The stationary orientations of compound **3** using the DFT/B3LYP/LANL2DZ level by using Gaussian 09.

Standard orientation:

Center Number	Atomic Number	Atomic Type	Coordinates (Angstroms) X	Y	Z
1	6	0	3.279024	-0.956112	0.804115
2	6	0	3.942804	-0.185978	-0.169771
3	6	0	3.312979	0.908975	-0.791903
4	6	0	1.992539	1.238713	-0.445711
5	6	0	1.358459	0.452994	0.521937
6	6	0	1.958199	-0.637379	1.159671
7	16	0	-0.384971	0.892092	0.997783
8	8	0	-1.214937	-0.210758	-0.322224
9	8	0	-0.721621	0.299669	2.465484
10	8	0	-0.682486	2.440183	0.630078
11	6	0	-2.613207	-0.202471	-0.367964
12	6	0	-3.273523	0.757416	-1.164616
13	6	0	-4.670783	0.706431	-1.265834
14	6	0	-5.401411	-0.286499	-0.572938
15	6	0	-4.724235	-1.234930	0.232189
16	6	0	-3.330556	-1.191554	0.344145
17	6	0	5.372152	-0.506090	-0.515012
18	9	0	5.691207	-0.173869	-1.838085

19	9	0	6.287443	0.197438	0.293873
20	9	0	5.673349	-1.863695	-0.349185
21	1	0	3.779537	-1.798098	1.270954
22	1	0	3.839174	1.489996	-1.542338
23	1	0	1.482856	2.083194	-0.898560
24	1	0	1.422720	-1.204901	1.914163
25	1	0	-5.197677	1.435196	-1.878638
26	1	0	-5.309153	-1.984876	0.757218
27	1	0	-2.785181	-1.901614	0.958028
28	6	0	-6.879622	-0.321867	-0.697166
29	8	0	-7.614726	-1.161597	-0.136791
30	1	0	-7.316790	0.467618	-1.339862
31	1	0	-2.690702	1.511281	-1.684152

Table S4. The stationary orientations of compound **4** using the DFT/B3LYP/LANL2DZ level by using Gaussian 09.

Standard orientation:

Center Number	Atomic Number	Atomic Type	Coordinates (Angstroms) X	Y	Z
1	6	0	3.415337	-1.408306	0.153179
2	6	0	3.747903	-0.081303	-0.175650
3	6	0	2.779817	0.942931	-0.157172
4	6	0	1.456536	0.643650	0.203647
5	6	0	1.161586	-0.682687	0.537829
6	6	0	2.093975	-1.724305	0.512435
7	16	0	-0.582889	-1.099044	1.046682
8	8	0	-1.429825	-0.459336	-0.608258
9	8	0	-0.712950	-2.710700	1.149798
10	8	0	-1.108576	-0.167955	2.264054
11	6	0	-2.656205	0.151849	-0.469557
12	6	0	-2.719959	1.537768	-0.136675
13	6	0	-3.975092	2.171061	-0.054468

14	6	0	-5.152138	1.438747	-0.302423
15	6	0	-5.096862	0.075550	-0.632184
16	6	0	-3.852352	-0.579437	-0.704327
17	6	0	-3.795684	-2.031004	-1.041804
18	8	0	-4.819217	-2.706355	-1.285039
19	6	0	5.170827	0.264749	-0.519083
20	9	0	5.258485	1.275511	-1.487487
21	9	0	5.890579	0.739836	0.597441
22	9	0	5.891769	-0.831466	-1.006851
23	1	0	4.170408	-2.186731	0.119054
24	1	0	3.050541	1.955879	-0.437738
25	1	0	0.675051	1.396843	0.198090
26	1	0	1.799619	-2.739327	0.760345
27	1	0	-4.046748	3.223048	0.200455
28	1	0	-6.112586	1.942431	-0.234304
29	1	0	-5.995434	-0.500941	-0.830063
30	1	0	-2.794951	-2.487722	-1.069593
31	8	0	-1.507865	2.179165	0.066969
32	6	0	-1.521004	3.548171	0.583836
33	1	0	-2.055066	3.596091	1.540618
34	1	0	-0.472233	3.810344	0.734280
35	1	0	-1.972527	4.236703	-0.141667

Table S5. The stationary orientations of compound **5** using the DFT/B3LYP/LANL2DZ level by using Gaussian 09.

Standard orientation:

Center Number	Atomic Number	Atomic Type	Coordinates (Angstroms) X	Y	Z
1	6	0	-3.390216	1.410196	0.015736
2	6	0	-4.128072	0.232877	-0.211348
3	6	0	-3.576829	-1.037772	0.042461
4	6	0	-2.261087	-1.140340	0.522694

5	6	0	-1.552331	0.046682	0.734424
6	6	0	-2.073202	1.322176	0.495430
7	16	0	0.185936	-0.079710	1.382932
8	8	0	1.013488	-0.147466	-0.338820
9	8	0	0.607865	1.303885	2.107871
10	8	0	0.395863	-1.510058	2.111067
11	6	0	2.414477	-0.154241	-0.378530
12	6	0	3.112536	-1.388859	-0.503562
13	6	0	4.516384	-1.338378	-0.629160
14	6	0	5.213087	-0.122308	-0.615499
15	6	0	4.493963	1.090257	-0.478257
16	6	0	3.089417	1.073164	-0.356407
17	6	0	-5.552993	0.334112	-0.684847
18	9	0	-5.929697	-0.751663	-1.485814
19	9	0	-6.470689	0.363953	0.384984
20	9	0	-5.789890	1.495550	-1.430522
21	1	0	-3.830854	2.380441	-0.188601
22	1	0	-4.159393	-1.934783	-0.141142
23	1	0	-1.812003	-2.104348	0.739966
24	1	0	-1.481746	2.211092	0.690572
25	1	0	5.047267	-2.280687	-0.735607
26	1	0	2.545610	2.005009	-0.249681
27	6	0	2.409392	-2.692112	-0.522082
28	8	0	2.999005	-3.788487	-0.658216
29	1	0	1.314838	-2.651946	-0.412446
30	1	0	6.293882	-0.121788	-0.708260
31	8	0	5.074582	2.347301	-0.455473
32	6	0	6.527381	2.465495	-0.554435
33	1	0	6.728492	3.536526	-0.502477
34	1	0	7.021934	1.953493	0.281413
35	1	0	6.891621	2.064684	-1.509634

Table S6. The stationary orientations of compound **6** using the DFT/B3LYP/LANL2DZ level by using Gaussian 09.

Standard orientation:

Center Number	Atomic Number	Atomic Type	Coordinates (Angstroms) X	Y	Z
1	6	0	3.659256	-1.413658	-0.154204
2	6	0	3.960913	-0.040426	-0.216894
3	6	0	2.975518	0.937716	0.023184
4	6	0	1.665331	0.542524	0.336443
5	6	0	1.400316	-0.829371	0.402643
6	6	0	2.351698	-1.823741	0.156528
7	16	0	-0.326472	-1.371865	0.845964
8	8	0	-1.192405	-0.563702	-0.703470
9	8	0	-0.391580	-2.987372	0.759829
10	8	0	-0.877918	-0.607277	2.164366
11	6	0	-2.445884	-0.027380	-0.482558
12	6	0	-2.581449	1.310788	-0.001756
13	6	0	-3.874244	1.862581	0.153308
14	6	0	-5.004273	1.098978	-0.156447
15	6	0	-4.876345	-0.231148	-0.626001
16	6	0	-3.592612	-0.786785	-0.776061
17	6	0	-6.073320	-1.036982	-0.955664
18	8	0	-7.248783	-0.623038	-0.860481
19	6	0	5.371685	0.393261	-0.506053
20	9	0	5.430021	1.631250	-1.161204
21	9	0	6.138352	0.542247	0.668805
22	9	0	6.065629	-0.524664	-1.304630
23	1	0	4.427378	-2.153325	-0.355348
24	1	0	3.223311	1.991682	-0.050227
25	1	0	0.872004	1.265150	0.497817
26	1	0	2.080580	-2.873660	0.206601
27	1	0	-3.999277	2.877221	0.515361
28	1	0	-6.002981	1.511644	-0.041030

29	1	0	-5.865997	-2.067335	-1.305112
30	1	0	-3.457442	-1.806875	-1.129186
31	8	0	-1.411216	1.992126	0.263196
32	6	0	-1.491046	3.329704	0.855548
33	1	0	-2.011043	3.295112	1.820315
34	1	0	-0.455469	3.639571	1.004685
35	1	0	-1.991565	4.028890	0.174348

Table S7. The stationary orientations of compound **7** using the DFT/B3LYP/LANL2DZ level by using Gaussian 09.

Standard orientation:

Center Number	Atomic Number	Atomic Type	Coordinates (Angstroms) X	Y	Z
1	6	0	3.219856	0.658630	0.592133
2	6	0	3.251516	-0.448341	-0.278063
3	6	0	2.216839	-0.682488	-1.203022
4	6	0	1.121161	0.193979	-1.256744
5	6	0	1.113300	1.276985	-0.372081
6	6	0	2.129581	1.541790	0.551960
7	16	0	-0.339360	2.446204	-0.426125
8	8	0	-1.531748	1.727103	0.927572
9	8	0	0.095060	3.870815	0.189750
10	8	0	-1.077713	2.283384	-1.857429
11	6	0	-2.338870	0.668258	0.538776
12	6	0	-2.006970	-0.650619	0.965389
13	6	0	-2.846289	-1.714605	0.606803
14	6	0	-3.999667	-1.482074	-0.173916
15	6	0	-4.327182	-0.169623	-0.592381
16	6	0	-3.498931	0.899106	-0.237678
17	6	0	4.440568	-1.369904	-0.255033
18	9	0	4.109903	-2.682389	-0.615425
19	9	0	5.450598	-0.964199	-1.151208
20	9	0	5.046565	-1.431849	1.006878

21	1	0	4.028976	0.825282	1.295820
22	1	0	2.257451	-1.541061	-1.865457
23	1	0	0.313981	0.048397	-1.967430
24	1	0	2.079551	2.410882	1.200532
25	1	0	-2.583889	-2.716539	0.938194
26	1	0	-5.220875	-0.018460	-1.190174
27	1	0	-3.717521	1.914424	-0.553110
28	6	0	-4.863208	-2.632039	-0.543398
29	8	0	-5.906844	-2.539231	-1.222053
30	1	0	-4.520473	-3.617489	-0.171070
31	8	0	-0.839144	-0.925240	1.678014
32	6	0	-0.710463	-0.331910	3.024358
33	1	0	-0.779138	0.758917	2.974362
34	1	0	-1.490350	-0.734101	3.683895
35	1	0	0.274115	-0.641110	3.380778

Table S8. The stationary orientations of compound **8** using the DFT/B3LYP/LANL2DZ level by using Gaussian 09.

Standard orientation:

Center Number	Atomic Number	Atomic Type	Coordinates (Angstroms) X	Y	Z
1	6	0	-3.896914	1.467367	-0.359287
2	6	0	-4.751740	0.350483	-0.293054
3	6	0	-4.325314	-0.865386	0.274734
4	6	0	-3.018653	-0.975897	0.777738
5	6	0	-2.191673	0.148966	0.694021
6	6	0	-2.587883	1.370016	0.139643
7	16	0	-0.463545	0.010774	1.367798
8	8	0	0.328301	-0.530291	-0.282206
9	8	0	0.097256	1.485642	1.740615
10	8	0	-0.391756	-1.203333	2.435868
11	6	0	1.710459	-0.785062	-0.269883
12	6	0	2.169277	-2.129296	-0.271871

13	6	0	3.570614	-2.317607	-0.339446
14	6	0	4.460066	-1.246012	-0.372904
15	6	0	3.992460	0.110976	-0.342238
16	6	0	2.581533	0.309815	-0.299208
17	6	0	-6.166109	0.475540	-0.790778
18	9	0	-6.673642	-0.735520	-1.277890
19	9	0	-7.055450	0.889589	0.222286
20	9	0	-6.290862	1.416392	-1.821382
21	1	0	-4.242651	2.395283	-0.802974
22	1	0	-4.996561	-1.717100	0.314818
23	1	0	-2.663196	-1.895273	1.232482
24	1	0	-1.907703	2.215283	0.112678
25	1	0	3.939061	-3.339993	-0.363842
26	1	0	2.145669	1.299925	-0.268387
27	6	0	1.253209	-3.279613	-0.225018
28	8	0	1.634090	-4.476067	-0.242436
29	1	0	0.179637	-3.035297	-0.176119
30	1	0	5.521871	-1.454758	-0.431295
31	7	0	4.869254	1.183913	-0.357659
32	6	0	6.333909	0.992939	-0.316280
33	1	0	6.771486	1.887920	0.142620
34	1	0	6.579292	0.158520	0.351437
35	6	0	4.383536	2.579946	-0.401241
36	1	0	5.176975	3.190665	-0.849324
37	1	0	3.525299	2.649551	-1.081157
38	6	0	4.015310	3.153158	0.985709
39	1	0	3.202926	2.586444	1.453547
40	1	0	4.880471	3.126128	1.660092
41	1	0	3.690809	4.197829	0.889217
42	6	0	6.965439	0.770140	-1.708722
43	1	0	8.053872	0.654654	-1.620896
44	1	0	6.561708	-0.128065	-2.189696
45	1	0	6.764288	1.623922	-2.367701

Table S9. The stationary orientations of compound **9** using the DFT/B3LYP/LANL2DZ level by using Gaussian 09.

Standard orientation:

Center Number	Atomic Number	Atomic Type	Coordinates (Angstroms) X	Y	Z
1	6	0	3.184882	-0.812542	1.035236
2	6	0	4.167870	-0.416115	0.108616
3	6	0	3.960983	0.679059	-0.752338
4	6	0	2.750644	1.388966	-0.694696
5	6	0	1.787884	0.967585	0.228876
6	6	0	1.969484	-0.111432	1.100578
7	16	0	0.179228	1.917283	0.321120
8	8	0	-0.728740	0.943488	-1.094752
9	8	0	-0.504513	1.549815	1.742225
10	8	0	0.486084	3.406629	-0.203868
11	6	0	5.446507	-1.200400	0.008114
12	9	0	6.534360	-0.411471	-0.387871
13	9	0	5.802471	-1.810284	1.218443
14	9	0	5.361999	-2.243818	-0.939329
15	1	0	3.368841	-1.646387	1.704968
16	1	0	4.736073	0.981407	-1.449235
17	1	0	2.567862	2.249020	-1.330943
18	1	0	1.198948	-0.377029	1.817551
19	6	0	-3.044171	0.556205	-0.366946
20	6	0	-4.090463	-0.409001	-0.113385
21	6	0	-1.762496	0.093556	-0.737172
22	6	0	-5.409611	-0.038121	0.310300
23	6	0	-3.805713	-1.816030	-0.267530
24	6	0	-1.489153	-1.302380	-0.868187
25	6	0	-6.385731	-0.999672	0.541176
26	1	0	-5.660109	1.004021	0.476035
27	6	0	-4.829455	-2.781140	-0.023410
28	6	0	-2.491695	-2.228907	-0.654216

29	1	0	-0.489394	-1.598539	-1.171330
30	6	0	-6.100224	-2.385479	0.369490
31	1	0	-7.377773	-0.693702	0.863110
32	1	0	-4.594054	-3.835876	-0.146423
33	1	0	-2.288941	-3.290829	-0.772384
34	1	0	-6.875715	-3.123645	0.554957
35	6	0	-3.323428	2.010146	-0.296061
36	8	0	-2.450054	2.896711	-0.181201
37	1	0	-4.381760	2.306259	-0.375303

Table S10. The stationary orientations of compound **10** using the DFT/B3LYP/LANL2DZ level by using Gaussian 09.

Standard orientation:

Center Number	Atomic Number	Atomic Type	Coordinates (Angstroms) X	Y	Z
1	7	0	3.264186	0.459217	0.861937
2	7	0	2.164139	0.201494	1.667383
3	6	0	1.415809	-0.836188	1.413754
4	6	0	1.618912	-1.893294	0.380593
5	6	0	0.553390	-2.276258	-0.477446
6	6	0	0.689659	-3.330690	-1.402501
7	6	0	1.911916	-4.014876	-1.496258
8	6	0	2.987044	-3.646738	-0.661903
9	6	0	2.841602	-2.595375	0.262698
10	8	0	-0.634828	-1.535985	-0.466973
11	16	0	-2.070999	-2.178796	0.620442
12	6	0	-3.215323	-0.758136	0.261662
13	8	0	-2.657930	-3.538505	-0.030663
14	6	0	-3.104617	0.390666	1.051089
15	6	0	-3.979947	1.455433	0.779488
16	6	0	-4.918357	1.335799	-0.262621
17	6	0	-5.006306	0.163854	-1.039152

18	6	0	-4.137378	-0.907819	-0.779507
19	8	0	-1.591287	-2.133873	2.167355
20	6	0	4.052723	1.586670	1.110769
21	8	0	3.839641	2.390266	2.041090
22	6	0	5.202764	1.778950	0.150238
23	6	0	5.781415	3.062833	0.076079
24	6	0	6.859261	3.278004	-0.799529
25	7	0	7.387872	2.300899	-1.582814
26	6	0	6.838659	1.063800	-1.496682
27	6	0	5.754614	0.756806	-0.652830
28	6	0	-5.884612	2.458989	-0.527617
29	9	0	-7.123631	2.258933	0.114212
30	9	0	-6.177318	2.599047	-1.891830
31	9	0	-5.409332	3.696428	-0.081264
32	1	0	3.416226	-0.084723	0.014568
33	1	0	0.548848	-0.936575	2.061791
34	1	0	-0.156062	-3.590873	-2.031773
35	1	0	2.024534	-4.829517	-2.205997
36	1	0	3.928597	-4.186412	-0.719720
37	1	0	3.661753	-2.352226	0.934136
38	1	0	-2.376738	0.451704	1.853899
39	1	0	-3.922841	2.367406	1.364391
40	1	0	-5.733661	0.091265	-1.841384
41	1	0	-4.184670	-1.828068	-1.353070
42	1	0	5.392046	3.860797	0.699450
43	1	0	7.322206	4.257251	-0.883558
44	1	0	7.289219	0.297642	-2.122225
45	1	0	5.393543	-0.267696	-0.621603

Table S11. The stationary orientations of compound **11** using the DFT/B3LYP/LANL2DZ level by using Gaussian 09.

Standard orientation:

Center Number	Atomic Number	Atomic Type	Coordinates (Angstroms) X	Y	Z
1	7	0	2.906508	-0.006660	1.896620
2	7	0	2.106423	0.257719	3.059492
3	6	0	0.949509	-0.319711	3.243383
4	6	0	0.142993	-1.332894	2.527274
5	6	0	0.224789	-1.614324	1.148695
6	6	0	-0.590476	-2.614943	0.589391
7	6	0	-1.490129	-3.363299	1.376623
8	6	0	-1.580143	-3.075135	2.750074
9	6	0	-0.786936	-2.062760	3.316484
10	8	0	-0.447942	-2.871102	-0.779951
11	16	0	-1.826852	-2.222115	-1.936615
12	6	0	-2.112021	-0.476942	-1.314261
13	8	0	-1.108158	-2.233993	-3.377804
14	6	0	-3.388237	-0.170202	-0.825055
15	6	0	-3.614069	1.136405	-0.366435
16	6	0	-2.564174	2.076585	-0.392599
17	6	0	-1.288062	1.735515	-0.874693
18	6	0	-1.057779	0.438528	-1.363489
19	8	0	-3.185011	-3.056080	-1.652456
20	6	0	2.726296	0.555339	0.646545
21	8	0	1.610957	0.971744	0.228154
22	6	0	3.962025	0.589890	-0.214623
23	6	0	3.818124	0.448381	-1.609997
24	6	0	4.964592	0.496128	-2.422040
25	7	0	6.216464	0.688410	-1.930012
26	6	0	6.347298	0.843651	-0.589017
27	6	0	5.258843	0.799964	0.302444
28	6	0	-2.806414	3.451796	0.166059

29	9	0	-4.107831	3.911457	-0.091288
30	9	0	-1.927479	4.410046	-0.347667
31	9	0	-2.657543	3.494169	1.568623
32	1	0	3.859310	-0.217067	2.176742
33	1	0	0.524843	-0.016567	4.203450
34	1	0	-2.272559	-3.632612	3.374406
35	1	0	-0.877082	-1.840803	4.377889
36	1	0	-4.169280	-0.923590	-0.809052
37	1	0	-4.594991	1.419527	0.002111
38	1	0	-0.474507	2.451602	-0.864312
39	1	0	-0.077517	0.165291	-1.735820
40	1	0	2.835831	0.305825	-2.047236
41	1	0	4.889078	0.378594	-3.499309
42	1	0	7.356685	1.014179	-0.224653
43	1	0	5.438302	0.972127	1.360851
44	1	0	0.864590	-1.041691	0.495413
45	1	0	-2.098099	-4.134683	0.913537

Table S12. The stationary orientations of compound **12** using the DFT/B3LYP/LANL2DZ level by using Gaussian 09.

Standard orientation:

Center Number	Atomic Number	Atomic Type	Coordinates (Angstroms) X	Y	Z
1	7	0	5.724756	-1.060104	-0.120110
2	7	0	5.467324	-2.397115	0.260868
3	6	0	4.262152	-2.807916	0.565461
4	6	0	2.912300	-2.218367	0.471086
5	6	0	1.882435	-2.845609	1.217986
6	6	0	0.558588	-2.398857	1.141222
7	6	0	0.250381	-1.317505	0.288601
8	6	0	1.250753	-0.706533	-0.499099
9	6	0	2.574227	-1.145589	-0.394206

10	8	0	-1.052270	-0.816836	0.256046
11	16	0	-2.247208	-1.580497	-1.038540
12	6	0	-3.717360	-0.549782	-0.552180
13	8	0	-1.757040	-1.163005	-2.525425
14	6	0	-4.564688	-1.044096	0.444602
15	6	0	-5.678446	-0.266715	0.801092
16	6	0	-5.899684	0.967314	0.159913
17	6	0	-5.030498	1.437379	-0.842866
18	6	0	-3.912723	0.669448	-1.208425
19	8	0	-2.518689	-3.131005	-0.653938
20	6	0	5.352563	0.075242	0.609189
21	8	0	4.540910	0.032852	1.563173
22	6	0	6.012929	1.356125	0.171671
23	6	0	6.054895	2.425361	1.089896
24	6	0	6.655935	3.634325	0.699142
25	7	0	7.196629	3.832340	-0.532071
26	6	0	7.138701	2.805138	-1.416523
27	6	0	6.560373	1.557953	-1.114115
28	6	0	-7.118242	1.773611	0.518210
29	9	0	-8.243925	1.414743	-0.251655
30	9	0	-6.933273	3.146942	0.314209
31	9	0	-7.497996	1.605778	1.856465
32	1	0	6.603181	-1.009603	-0.624749
33	1	0	4.280912	-3.842968	0.911567
34	1	0	-0.232835	-2.865369	1.719397
35	1	0	0.977551	0.104760	-1.166684
36	1	0	3.338997	-0.675547	-1.001474
37	1	0	-4.371293	-2.004290	0.912183
38	1	0	-6.357243	-0.611670	1.574307
39	1	0	-5.214957	2.392438	-1.323836
40	1	0	-3.228838	0.998708	-1.984301
41	1	0	5.621138	2.303706	2.076923
42	1	0	6.710998	4.476256	1.383530
43	1	0	7.564597	2.996392	-2.397853

44	1	0	6.519009	0.792939	-1.884653
45	1	0	2.129079	-3.682430	1.867517

Table S13. The stationary orientations of compound **13** using the DFT/B3LYP/LANL2DZ level by using Gaussian 09.

Standard orientation:

Center Number	Atomic Number	Atomic Type	Coordinates (Angstroms) X	Y	Z
1	7	0	3.547892	-0.780453	-0.954071
2	7	0	4.130724	0.090449	-1.834967
3	6	0	3.698055	1.307500	-2.046400
4	6	0	2.615921	2.177327	-1.520598
5	6	0	1.606012	1.815092	-0.500521
6	6	0	0.730878	2.941199	0.133166
7	6	0	0.624832	4.196922	-0.696175
8	6	0	1.486369	4.415211	-1.720942
9	6	0	2.500119	3.435980	-2.085661
10	8	0	1.404918	0.640970	-0.091698
11	16	0	-0.895450	1.274070	1.769065
12	6	0	-2.276511	0.415362	0.837269
13	8	0	-1.701485	2.169353	2.870650
14	6	0	-1.955949	-0.501096	-0.175284
15	6	0	-3.004544	-1.155995	-0.840951
16	6	0	-4.337991	-0.876727	-0.480709
17	6	0	-4.638361	0.044835	0.540079
18	6	0	-3.593958	0.702110	1.210772
19	8	0	-0.629179	2.504099	0.328311
20	6	0	4.097624	-2.060151	-0.808709
21	8	0	5.073682	-2.477075	-1.465926
22	6	0	3.404942	-2.930695	0.211887
23	6	0	3.659805	-4.317192	0.164561
24	6	0	3.043553	-5.158901	1.105678
25	7	0	2.211639	-4.702185	2.078988

26	6	0	1.981002	-3.366139	2.128776
27	6	0	2.546779	-2.447501	1.224328
28	6	0	-5.451796	-1.613909	-1.169346
29	9	0	-5.169371	-1.877779	-2.518359
30	9	0	-6.665273	-0.914024	-1.138157
31	9	0	-5.714097	-2.869615	-0.581208
32	1	0	2.678763	-0.511942	-0.478003
33	1	0	4.307835	1.816140	-2.793744
34	1	0	1.434166	5.335857	-2.295369
35	1	0	3.193435	3.708506	-2.879865
36	1	0	-0.922671	-0.695210	-0.446860
37	1	0	-2.787513	-1.864221	-1.634402
38	1	0	-5.671220	0.252795	0.800583
39	1	0	-3.781354	1.419029	2.005413
40	1	0	4.332059	-4.710248	-0.590872
41	1	0	3.215485	-6.231612	1.091629
42	1	0	1.324942	-3.026211	2.925984
43	1	0	2.327821	-1.391272	1.346285
44	1	0	-0.150450	4.902466	-0.416059
45	8	0	1.421979	3.144598	1.399833
46	6	0	0.978988	4.282621	2.235208
47	1	0	-0.100488	4.230032	2.413994
48	1	0	1.518486	4.153053	3.175242
49	1	0	1.260849	5.235115	1.772938

Table S14. The stationary orientations of compound **14** using the DFT/B3LYP/LANL2DZ level by using Gaussian 09.

Standard orientation:

Center Number	Atomic Number	Atomic Type	Coordinates (Angstroms) X	Y	Z
1	7	0	3.076900	-1.203831	-0.916429
2	7	0	1.961919	-1.028832	-1.725795

3	6	0	1.260292	0.066476	-1.613986
4	6	0	1.534938	1.263665	-0.773371
5	6	0	0.494843	1.867620	-0.010006
6	6	0	0.693428	3.045779	0.721950
7	6	0	1.963200	3.657006	0.721155
8	6	0	3.019210	3.082109	-0.020414
9	6	0	2.794861	1.900797	-0.751127
10	8	0	-0.744373	1.220487	0.087656
11	16	0	-2.130202	1.768719	-1.103616
12	6	0	-3.386248	0.546744	-0.481002
13	8	0	-2.588809	3.272986	-0.726143
14	6	0	-3.409130	-0.725726	-1.060172
15	6	0	-4.367745	-1.639064	-0.590277
16	6	0	-5.253174	-1.252864	0.433284
17	6	0	-5.206821	0.038259	0.994522
18	6	0	-4.253845	0.961133	0.534932
19	8	0	-1.682959	1.387383	-2.613027
20	6	0	3.884542	-2.330692	-1.076567
21	8	0	3.774658	-3.137593	-2.022847
22	6	0	4.957534	-2.486832	-0.024882
23	6	0	6.097627	-3.246970	-0.357872
24	6	0	7.108017	-3.420125	0.603378
25	7	0	7.040454	-2.896938	1.856388
26	6	0	5.932716	-2.183425	2.178460
27	6	0	4.873917	-1.952554	1.279627
28	6	0	-6.306789	-2.216683	0.908816
29	9	0	-7.540090	-2.025563	0.253258
30	9	0	-6.574875	-2.079928	2.278307
31	9	0	-5.953229	-3.552795	0.691824
32	1	0	3.285089	-0.535477	-0.176907
33	1	0	0.372005	0.092328	-2.240123
34	1	0	3.999544	3.545730	-0.049818
35	1	0	3.605151	1.498779	-1.354349
36	1	0	-2.719241	-0.992592	-1.854459

37	1	0	-4.415119	-2.639166	-1.008205
38	1	0	-5.895874	0.316813	1.785353
39	1	0	-4.198106	1.966850	0.939305
40	1	0	6.174151	-3.692522	-1.344155
41	1	0	8.001728	-3.994453	0.375701
42	1	0	5.894886	-1.793584	3.192330
43	1	0	4.003184	-1.401665	1.625481
44	1	0	-0.117800	3.484882	1.292293
45	8	0	2.067955	4.813740	1.479181
46	6	0	3.338544	5.532640	1.507162
47	1	0	3.622336	5.871353	0.501855
48	1	0	3.162341	6.395798	2.150734
49	1	0	4.136506	4.909407	1.932949

Table S15. The stationary orientations of compound **15** using the DFT/B3LYP/LANL2DZ level by using Gaussian 09.

Standard orientation:

Center Number	Atomic Number	Atomic Type	Coordinates (Angstroms) X	Y	Z
1	7	0	-0.054430	3.846539	-0.073095
2	7	0	-1.142526	4.341126	-0.858208
3	6	0	-2.254731	3.672572	-1.022442
4	6	0	-2.840552	2.417179	-0.523497
5	6	0	-2.157816	1.381791	0.150220
6	6	0	-2.853165	0.253773	0.612600
7	6	0	-4.257248	0.117770	0.392347
8	6	0	-4.943369	1.139945	-0.294031
9	6	0	-4.237830	2.261814	-0.743438
10	8	0	-2.164956	-0.749033	1.265718
11	16	0	-2.005976	-2.387088	0.186886
12	6	0	-0.153113	-2.419430	-0.040588
13	8	0	-2.423689	-3.659634	1.090453

14	6	0	0.413668	-1.568789	-0.997194
15	6	0	1.806778	-1.615929	-1.167320
16	6	0	2.575983	-2.499825	-0.384744
17	6	0	1.976941	-3.342998	0.570625
18	6	0	0.584922	-3.304877	0.751944
19	8	0	-2.690100	-2.074262	-1.251720
20	6	0	0.816932	2.827674	-0.423471
21	8	0	0.507265	1.885426	-1.202494
22	6	0	2.172213	2.911500	0.233215
23	6	0	2.862834	1.716209	0.518055
24	6	0	4.138533	1.784660	1.104951
25	7	0	4.752866	2.958426	1.409115
26	6	0	4.092364	4.106621	1.117395
27	6	0	2.811956	4.134258	0.533703
28	6	0	4.070615	-2.499116	-0.536441
29	9	0	4.484654	-2.250927	-1.849971
30	9	0	4.660330	-3.704364	-0.140017
31	9	0	4.681901	-1.493283	0.254037
32	1	0	0.328153	4.602201	0.485474
33	1	0	-2.942191	4.251064	-1.644431
34	1	0	-6.007304	1.058940	-0.489570
35	1	0	-4.777524	3.040352	-1.278999
36	1	0	-0.191707	-0.893139	-1.591981
37	1	0	2.276842	-0.972292	-1.903772
38	1	0	2.584325	-4.023958	1.157782
39	1	0	0.086770	-3.946974	1.471629
40	1	0	2.411124	0.759289	0.284239
41	1	0	4.692559	0.879865	1.334772
42	1	0	4.613925	5.030273	1.353784
43	1	0	2.367209	5.095802	0.289478
44	1	0	-1.088776	1.407424	0.294572
45	8	0	-4.812360	-1.043921	0.861673
46	6	0	-6.188189	-1.387558	0.503394
47	1	0	-6.894600	-0.669910	0.940197

48	1	0	-6.303044	-1.425821	-0.586773
49	1	0	-6.349123	-2.377262	0.931559

Table S16. The stationary orientations of compound **16** using the DFT/B3LYP/LANL2DZ level by using Gaussian 09.

Standard orientation:

Center Number	Atomic Number	Atomic Type	Coordinates (Angstroms) X	Y	Z
1	7	0	-5.498887	-1.348148	0.895090
2	7	0	-4.774199	-2.510689	1.251065
3	6	0	-3.476126	-2.578372	1.098447
4	6	0	-2.429634	-1.592041	0.763575
5	6	0	-1.193337	-2.095694	0.295985
6	6	0	-0.126822	-1.234005	0.023455
7	6	0	-0.272954	0.162767	0.253584
8	6	0	-1.495703	0.663870	0.756351
9	6	0	-2.568765	-0.197153	0.991271
10	8	0	0.772084	1.018286	-0.028746
11	16	0	2.002042	1.328807	1.487531
12	6	0	3.632045	0.847709	0.714857
13	8	0	2.035093	2.919456	1.783694
14	6	0	3.946025	-0.512867	0.619299
15	6	0	5.192764	-0.854862	0.072175
16	6	0	6.069285	0.160651	-0.360480
17	6	0	5.722710	1.520037	-0.253678
18	6	0	4.479860	1.877843	0.296019
19	8	0	1.605093	0.258405	2.637870
20	6	0	-5.453787	-0.712397	-0.345011
21	8	0	-4.523360	-0.898090	-1.166608
22	6	0	-6.585606	0.241724	-0.630389
23	6	0	-6.377744	1.210476	-1.634711
24	6	0	-7.409221	2.116150	-1.935773
25	7	0	-8.615724	2.103511	-1.310799

26	6	0	-8.817014	1.160665	-0.356611
27	6	0	-7.841265	0.217248	0.015758
28	6	0	7.379655	-0.225981	-0.988898
29	9	0	7.944475	-1.364598	-0.396914
30	9	0	8.337665	0.791718	-0.914482
31	9	0	7.243963	-0.534110	-2.359831
32	1	0	-6.359824	-1.295509	1.425945
33	1	0	-3.104601	-3.576824	1.335691
34	1	0	-1.575839	1.729916	0.949453
35	1	0	-3.500546	0.198918	1.378154
36	1	0	3.243879	-1.273172	0.946736
37	1	0	5.480969	-1.898352	-0.006605
38	1	0	6.414052	2.288911	-0.582525
39	1	0	4.185726	2.916481	0.410312
40	1	0	-5.431156	1.238018	-2.163790
41	1	0	-7.276936	2.877462	-2.699433
42	1	0	-9.794758	1.163059	0.117634
43	1	0	-8.100066	-0.520423	0.771032
44	1	0	-1.052404	-3.160959	0.132931
45	8	0	1.090010	-1.765020	-0.421860
46	6	0	1.440100	-1.465569	-1.826097
47	1	0	2.405203	-1.946792	-1.997670
48	1	0	0.683835	-1.892964	-2.496430
49	1	0	1.518382	-0.383980	-1.977962

Table S17. The stationary orientations of compound **17** using the DFT/B3LYP/LANL2DZ level by using Gaussian 09.

Standard orientation:

Center Number	Atomic Number	Atomic Type	Coordinates (Angstroms) X	Y	Z
1	7	0	-2.399851	-2.031711	-0.339252
2	7	0	-1.024045	-2.066214	-0.536831
3	6	0	-0.350098	-0.943043	-0.546780

4	6	0	-0.779502	0.440116	-0.271213
5	6	0	-0.133875	1.532602	-0.926473
6	6	0	-0.464263	2.870568	-0.676010
7	6	0	-1.462190	3.215347	0.278622
8	6	0	-2.099243	2.128543	0.957363
9	6	0	-1.774202	0.798178	0.674460
10	8	0	0.820964	1.252882	-1.905633
11	16	0	2.650673	1.758382	-1.523840
12	6	0	3.353149	0.237031	-0.700747
13	8	0	3.301730	1.925180	-2.988908
14	6	0	3.414083	0.214378	0.697085
15	6	0	3.959180	-0.926550	1.308194
16	6	0	4.415504	-1.993491	0.511006
17	6	0	4.342042	-1.941041	-0.894347
18	6	0	3.800049	-0.806252	-1.519870
19	8	0	2.635702	2.958090	-0.432094
20	6	0	-3.115565	-3.221643	-0.212927
21	8	0	-2.584209	-4.339442	-0.042896
22	6	0	-4.616833	-3.055748	-0.241315
23	6	0	-5.400880	-4.052744	0.375070
24	6	0	-6.800795	-3.932346	0.352968
25	7	0	-7.449657	-2.899252	-0.247263
26	6	0	-6.691864	-1.953290	-0.856273
27	6	0	-5.284633	-1.987445	-0.879637
28	6	0	5.046150	-3.187419	1.176158
29	9	0	6.430067	-3.006509	1.386283
30	9	0	4.919488	-4.356360	0.419019
31	9	0	4.497427	-3.449668	2.437671
32	1	0	-2.902771	-1.148334	-0.389453
33	1	0	0.689556	-1.084180	-0.828501
34	1	0	0.072800	3.628644	-1.229685
35	1	0	-2.837777	2.320591	1.726749
36	1	0	-2.258465	0.021260	1.260443
37	1	0	3.066944	1.060496	1.281552

38	1	0	4.019531	-0.986469	2.390055
39	1	0	4.692831	-2.775477	-1.492772
40	1	0	3.741281	-0.731230	-2.601215
41	1	0	-4.914118	-4.899627	0.847284
42	1	0	-7.432193	-4.680291	0.824549
43	1	0	-7.235472	-1.149314	-1.345676
44	1	0	-4.750084	-1.211089	-1.420968
45	7	0	-1.797674	4.540041	0.525658
46	6	0	-2.859904	4.891595	1.490130
47	6	0	-2.397939	4.936665	2.965585
48	1	0	-3.694956	4.187862	1.380646
49	1	0	-3.252218	5.875344	1.206125
50	1	0	-3.247963	5.169376	3.620817
51	1	0	-1.974416	3.976393	3.282031
52	1	0	-1.632907	5.707451	3.114255
53	6	0	-1.034572	5.645253	-0.099045
54	6	0	0.362113	5.893287	0.517688
55	1	0	-1.640046	6.553274	0.000833
56	1	0	-0.938116	5.458888	-1.177534
57	1	0	0.854674	6.725161	-0.003236
58	1	0	0.280032	6.156748	1.578901
59	1	0	1.009864	5.014197	0.431401

Table S18. The stationary orientations of compound **18** using the DFT/B3LYP/LANL2DZ level by using Gaussian 09.

Standard orientation:

Center Number	Atomic Number	Atomic Type	Coordinates (Angstroms) X	Y	Z
1	7	0	0.752418	-1.672150	0.907773
2	7	0	0.033929	-0.495469	0.626835
3	6	0	-1.262750	-0.526894	0.771593
4	6	0	-2.112979	0.607737	0.369482
5	6	0	-1.945925	1.253668	-0.875090

6	6	0	-2.836247	2.289454	-1.299891
7	6	0	-3.894771	2.666757	-0.497389
8	6	0	-4.121805	2.030721	0.764107
9	6	0	-3.226722	0.988617	1.203620
10	8	0	-0.997480	0.801092	-1.781133
11	16	0	0.479749	1.993596	-2.220604
12	6	0	1.911427	1.500589	-1.132301
13	8	0	0.818721	1.566347	-3.739122
14	6	0	2.119651	2.221107	0.046901
15	6	0	3.203844	1.848182	0.856217
16	6	0	4.030727	0.778427	0.462017
17	6	0	3.806099	0.083112	-0.742350
18	6	0	2.725477	0.447950	-1.561043
19	8	0	0.028572	3.495723	-1.811622
20	6	0	0.435168	-3.026762	0.745621
21	8	0	1.144208	-3.880641	1.337261
22	6	0	-0.649172	-3.450787	-0.208837
23	6	0	-1.295596	-4.678971	0.040443
24	6	0	-2.248490	-5.146105	-0.881863
25	7	0	-2.567323	-4.478789	-2.021276
26	6	0	-1.924054	-3.307805	-2.264912
27	6	0	-0.967520	-2.752992	-1.394325
28	6	0	5.138572	0.330567	1.373939
29	9	0	5.650279	1.370310	2.158819
30	9	0	6.216880	-0.233509	0.684035
31	9	0	4.695789	-0.659692	2.281530
32	1	0	1.666929	-1.491183	1.311926
33	1	0	-1.774395	-1.407409	1.169902
34	1	0	-2.663100	2.753185	-2.264928
35	1	0	-4.573855	3.451204	-0.821762
36	1	0	1.466613	3.044338	0.317748
37	1	0	3.405162	2.385186	1.777536
38	1	0	4.466974	-0.724497	-1.040788
39	1	0	2.534400	-0.056285	-2.503068

40	1	0	-1.041827	-5.256447	0.923386
41	1	0	-2.771371	-6.084112	-0.716464
42	1	0	-2.187516	-2.800736	-3.188710
43	1	0	-0.501381	-1.808443	-1.654021
44	6	0	-3.465501	0.386259	2.482228
45	1	0	-2.787288	-0.373980	2.858193
46	6	0	-4.540432	0.779926	3.269218
47	1	0	-4.704306	0.311970	4.236407
48	6	0	-5.429947	1.801032	2.822438
49	6	0	-5.217509	2.414797	1.595132
50	1	0	-5.885145	3.200924	1.250177
51	1	0	-6.267039	2.097939	3.448572

Table S19. Interactions types and distances of compounds **1**, **4**, and **5** for PC3; **4**, **6**, **9**, **10**, and **14** for DLD-1, and cisplatin, as control compound for both target models. ADP: adenosine-5'-diphosphate as co-crystal ligand of PC3-FAK; PTR: (2*S*)-2-amino-3-(4-phosphonooxyphenyl)propanoic acid as co-crystal ligand of PC3-Scr; K56: 3-[[1-(6,7-dimethoxyquinazolin-4-yl)piperidin-4-yl]methyl]-1,4-dihydroquinazolin-2-one as co-crystal ligand of DLD-1-TNKS.

Distance Å	Bonding	Binding site of target (PC3-FAK)	Binding site of ligand (ADP)
4.9975	Electrostatic	A:LYS454:NZ	A:ADP:O2B
3.4545	Electrostatic	A:LYS454:NZ	A:ADP:O3B
5.0904	Electrostatic	A:LYS454:NZ	A:ADP:O2A
1.8900	Hydrogen Bond	A:GLN432:HN	A:ADP:O2B
1.8586	Hydrogen Bond	A:LYS454:HZ1	A:ADP:O1B
1.9904	Hydrogen Bond	A:LYS454:HZ3	A:ADP:O1A
1.8632	Hydrogen Bond	A:CYS502:HN	A:ADP:N1
1.5649	Hydrogen Bond	A:GLU506:OE1	A:ADP:HO2'
1.9282	Hydrogen Bond	A:GLU500:O	A:ADP:H62
3.0832	Hydrogen Bond	A:GLY431:HA1	A:ADP:O3A
2.7200	Hydrogen Bond	A:GLY431:HA2	A:ADP:O3A
2.1682	Hydrogen Bond	A:CYS502:O	A:ADP:H2
2.8088	Hydrophobic	A:VAL436:HG11	A:ADP
4.5845	Hydrophobic	A:ALA452	A:ADP

4.7790	Hydrophobic	A:LEU553	A:ADP
5.3780	Hydrophobic	A:ILE428	A:ADP
3.9249	Hydrophobic	A:ALA452	A:ADP
4.9154	Hydrophobic	A:CYS502	A:ADP
4.4336	Hydrophobic	A:LEU553	A:ADP
**Distance Å**	**Bonding**	**Binding site of target (PC3-FAK)**	**Binding site of ligand (cisplatin)**
2.8504	Hydrogen Bond	A:LYS454:HE2	:cisplatin:CL1
4.2737	Hydrophobic	A:VAL436	:cisplatin:CL1
3.9874	Hydrophobic	A:LYS454	:cisplatin:CL1
4.4233	Hydrophobic	A:VAL436	:cisplatin:CL4
4.7543	Hydrophobic	A:LEU553	:cisplatin:CL4
**Distance Å**	**Bonding**	**Binding site of target (PC3-FAK)**	**Binding site of ligand (1)**
2.3022	Hydrogen Bond;Halogen	A:ARG426:HH12	:1:F
1.9891	Hydrogen Bond;Halogen	A:ARG426:HH22	:1:F
3.0160	Hydrogen Bond	A:CYS502:HN	:1:O
1.7625	Hydrogen Bond	A:CYS502:HN	:1:O
2.2994	Hydrogen Bond	A:LEU501:HA	:1:O
2.2546	Hydrogen Bond	A:GLU500:O	:1:H9
3.1172	Halogen	A:ARG426:CZ	:1:F
3.2284	Halogen	A:THR503:O	:1:F
2.8878	Halogen	A:THR503:O	:1:F
4.2100	Hydrophobic	A:ILE428	:1:C
3.5009	Hydrophobic	A:ILE428	:1
5.1373	Hydrophobic	A:VAL436	:1
4.0284	Hydrophobic	A:ALA452	:1
5.0972	Hydrophobic	A:LYS454	:1
5.2369	Hydrophobic	A:MET499	:1
5.0117	Hydrophobic	A:LEU553	:1
**Distance Å**	**Bonding**	**Binding site of target (PC3-FAK)**	**Binding site of ligand (4)**
2.2699	Hydrogen Bond;Halogen	A:ARG426:HH12	:4:F
1.9530	Hydrogen Bond;Halogen	A:ARG426:HH22	:4:F
2.9905	Hydrogen Bond;Halogen	A:GLN438:HE22	:4:F
1.8323	Hydrogen Bond	A:CYS502:HN	:4:O
2.6985	Hydrogen Bond	A:LEU501:HA	:4:O
2.5855	Hydrogen Bond	A:GLU500:O	:4:H11
3.0803	Halogen	A:ARG426:CZ	:4:F
3.6755	Halogen	A:ARG426:CZ	:4:F
2.8831	Halogen	A:THR503:O	:4:F
3.3132	Halogen	A:THR503:O	:4:F
4.2233	Hydrophobic	A:ILE428	:4:C
4.1552	Hydrophobic	A:VAL436	:4:C
3.5859	Hydrophobic	A:ILE428	:4
4.6911	Hydrophobic	A:VAL436	:4
4.3785	Hydrophobic	A:ALA452	:4
5.0635	Hydrophobic	A:LYS454	:4
5.0046	Hydrophobic	A:LEU553	:4
**Distance Å**	**Bonding**	**Binding site of target (PC3-FAK)**	**Binding site of ligand (5)**

1.9237	Hydrogen Bond	A:LYS454:HZ3	:5:O
2.2402	Hydrogen Bond	A:LYS454:HZ3	:5:O
2.0859	Hydrogen Bond	A:CYS502:HN	:5:O
1.8093	Hydrogen Bond	A:ASP564:HN	:5:O
2.6072	Hydrogen Bond	A:GLY563:HA1	:5:O
2.5126	Hydrogen Bond	A:GLY563:HA2	:5:O
2.6842	Hydrogen Bond	A:ASP564:HA	:5:O
2.8124	Hydrogen Bond	A:ASP564:O	:5:H8
2.3271	Hydrogen Bond	A:CYS502:O	:5:H9
3.1857	Halogen	A:GLU506:CD	:5:F
2.7367	Halogen	A:GLU506:OE1	:5:F
2.4876	Halogen	A:ARG550:O	:5:F
2.6631	Halogen	A:ARG550:O	:5:F
5.9866	Other	A:MET499:SD	:5
5.2388	Hydrophobic	A:ILE428	:5:C
5.3756	Hydrophobic	A:LEU501	:5:C
4.4553	Hydrophobic	A:CYS502	:5:C
4.2588	Hydrophobic	A:LEU553	:5:C
4.9543	Hydrophobic	A:LEU553	:5
3.9067	Hydrophobic	A:ALA452	:5
4.7850	Hydrophobic	A:VAL484	:5
5.1734	Hydrophobic	A:CYS502	:5
4.5862	Hydrophobic	A:LEU553	:5

**Distance Å**	**Bonding**	**Binding site of target (PC3-Src)**	**Binding site of ligand (cisplatin)**
1.8722	Hydrogen Bond	A:GLU147:OE2	:cisplatin:H1
2.70131	Halogen	A:CYS245:O	:cisplatin:CL4
5.31332	Hydrophobic	A:VAL244	:cisplatin:CL4
5.16797	Hydrophobic	A:PHE150	:cisplatin:CL4
**Distance Å**	**Bonding**	**Binding site of target (PC3-Src)**	**Binding site of ligand (1)**
1.8622	Hydrogen Bond;Halogen	A:LEU89:HA	:1:F
2.3574	Hydrogen Bond	A:LYS104:HE1	:1:F
2.9958	Halogen	A:ALA88:O	:1:F
2.8539	Halogen	A:LEU89:O	:1:F
2.7724	Halogen	A:ALA145:O	:1:F
2.2850	Hydrophobic	A:GLU147:HA	:1
4.4912	Other	A:PHE150	:1:S
2.7620	Other	A:TYR149:O	:1
4.4905	Hydrophobic	A:LEU89	:1:C
4.6376	Hydrophobic	A:LYS104	:1:C
4.0845	Hydrophobic	A:LEU89	:1
**Distance Å**	**Bonding**	**Binding site of target (PC3-Src)**	**Binding site of ligand (4)**
2.9293	Hydrogen Bond	A:LYS104:HZ1	:4:O
2.8495	Hydrogen Bond	A:LEU89:HA	:4:O
2.3025	Hydrogen Bond	A:LYS104:HE2	:4:O
2.0301	Hydrogen Bond;Halogen	A:PRO246:HA	:4:F

2.4858	Hydrogen Bond	A:ALA145:O	:4:H11
2.7789	Halogen	A:VAL244:O	:4:F
2.5984	Halogen	A:VAL244:O	:4:F
2.7801	Halogen	A:PRO246:N	:4:F
2.8642	Hydrogen Bond;Electrostatic	A:LYS104:HZ1	:4
3.5325	Electrostatic	A:GLU147:OE2	:4
3.9663	Hydrophobic	A:GLU146:C,O;GLU147:N	:4
4.6554	Hydrophobic	A:VAL244	:4:C
4.4226	Hydrophobic	A:PRO246	:4:C
3.4542	Hydrophobic	A:PHE150	:4:C
5.3461	Hydrophobic	A:VAL244	:4
4.2988	Hydrophobic	A:LEU89	:4
**Distance Å**	**Bonding**	**Binding site of target ((PC3-Src)**	**Binding site of ligand (5)**
2.6365	Hydrogen Bond	A:LEU89:HA	:5:O
2.6889	Hydrogen Bond	A:LYS104:HE1	:5:O
2.3139	Hydrogen Bond	A:LYS104:HE2	:5:O
2.7247	Hydrogen Bond	A:PHE150:HA	:5:O
2.2358	Hydrogen Bond;Halogen	A:PRO246:HA	:5:F
2.3993	Hydrogen Bond	A:ALA145:O	:5:H8
2.6974	Halogen	A:VAL244:O	:5:F
2.5487	Halogen	A:VAL244:O	:5:F
2.8923	Halogen	A:PRO246:N	:5:F
2.8212	Hydrogen Bond;Electrostatic	A:LYS104:HZ1	:5
3.4391	Electrostatic	A:GLU147:OE2	:5
4.5808	Hydrophobic	A:VAL244	:5:C
3.6743	Hydrophobic	A:PHE150	:5:C
5.3434	Hydrophobic	A:VAL244	:5
4.4497	Hydrophobic	A:LEU89	:5

**Distance Å**	**Bonding**	**Binding site of target (DLD-1)**	**Binding site of ligand (K56)**
2.4420	Hydrogen Bond	A:ASP1198:HN	A:K56:N2
1.8570	Hydrogen Bond	A:TYR1213:HN	A:K56:O3
2.3067	Hydrogen Bond	A:HIS1184:HE1	A:K56:O3
2.6094	Hydrogen Bond	A:ILE1212:HA	A:K56:O3
2.3729	Hydrogen Bond	A:GLY1211:O	A:K56:H9
2.3577	Hydrogen Bond	A:ASP1198:O	A:K56:H13
2.0066	Hydrogen Bond	A:GLY1196:O	A:K56:H19
4.8647	Hydrophobic	A:PHE1188	A:K56
4.8001	Hydrophobic	A:HIS1201	A:K56
3.6711	Hydrophobic	A:HIS1201	A:K56
4.8690	Hydrophobic	A:ALA1202	A:K56
5.0386	Hydrophobic	A:ILE1212	A:K56
3.7908	Hydrophobic	A:PHE1188	A:K56:C13
4.6408	Hydrophobic	A:HIS1201	A:K56:C13

5.1838	Hydrophobic	A:ILE1228	A:K56
4.6010	Hydrophobic	A:ALA1191	A:K56
4.9115	Hydrophobic	A:ILE1192	A:K56
4.4604	Hydrophobic	A:ALA1191	A:K56
**Distance Å**	**Bonding**	**Binding site of target (DLD-1)**	**Binding site of ligand (cisplatin)**
2.3851	Hydrogen Bond	A:GLY1211:O	:cisplatin:H4
2.6449	Hydrogen Bond	A:HIS1184:HE1	:cisplatin:CL4
4.4581	Hydrophobic	A:ALA1202	:cisplatin:CL1
4.5372	Hydrophobic	A:ILE1212	:cisplatin:CL1
4.3411	Hydrophobic	A:HIS1184	:cisplatin:CL4
**Distance Å**	**Bonding**	**Binding site of target (DLD-1)**	**Binding site of ligand (4)**
2.2489	Hydrogen Bond;Halogen	A:TYR1203:HN	:4:F
2.9138	Hydrogen Bond;Halogen	A:GLY1211:HN	:4:F
2.6758	Hydrogen Bond;Halogen	A:GLY1206:HA2	:4:F
1.7894	Hydrogen Bond	A:ASP1198:O	:4:H10
3.1387	Halogen	A:TYR1203:O	:4:F
3.1548	Halogen	A:TYR1203:O	:4:F
2.8652	Halogen	A:GLY1209:O	:4:F
2.9829	Halogen	A:GLY1211:O	:4:F
5.7794	Hydrophobic	A:PHE1188	:4
5.1690	Hydrophobic	A:HIS1201	:4
4.3792	Hydrophobic	A:ALA1202	:4:C
3.8666	Hydrophobic	A:ILE1212	:4:C
4.9706	Hydrophobic	A:TYR1213	:4:C
4.0650	Hydrophobic	A:ALA1202	:4
5.2233	Hydrophobic	A:ALA1191	:4
4.8248	Hydrophobic	A:ILE1192	:4
**Distance Å**	**Bonding**	**Binding site of target (DLD-1)**	**Binding site of ligand (6)**
1.7759	Hydrogen Bond	A:GLY1185:HN	:6:O
1.8881	Hydrogen Bond;Halogen	A:TYR1203:HN	:6:F
2.4273	Hydrogen Bond;Halogen	A:GLY1206:HN	:6:F
2.0328	Hydrogen Bond	A:TYR1213:HN	:6:O
2.2725	Hydrogen Bond	A:HIS1184:HA	:6:O
1.9628	Hydrogen Bond	A:HIS1184:HE1	:6:O
1.9143	Hydrogen Bond	A:SER1186:HB2	:6:O
2.6174	Hydrogen Bond;Halogen	A:GLY1206:HA1	:6:F
2.1912	Hydrogen Bond;Halogen	A:GLY1206:HA2	:6:F
2.7215	Halogen	A:TYR1203:O	:6:F
3.0729	Halogen	A:TYR1203:O	:6:F
3.6881	Halogen	A:GLY1205:C	:6:F
2.9002	Halogen	A:GLY1209:O	:6:F
4.7509	Other	A:HIS1184	:6:S
5.0448	Hydrophobic	A:TYR1213	:6
3.7327	Hydrophobic	A:TYR1224	:6
5.3829	Hydrophobic	A:TYR1213	:6:C
3.8693	Hydrophobic	A:TYR1213	:6:C

4.5956	Hydrophobic	A:TYR1224	:6:C
**Distance Å**	**Bonding**	**Binding site of target (DLD-1)**	**Binding site of ligand (9)**
2.0410	Hydrogen Bond;Halogen	A:TYR1203:HN	:9:F
2.3588	Hydrogen Bond;Halogen	A:GLY1206:HN	:9:F
2.0515	Hydrogen Bond	A:MET1207:HN	:9:O
2.1737	Hydrogen Bond	A:HIS1184:HE1	:9:O
2.6803	Hydrogen Bond;Halogen	A:GLY1205:HA2	:9:F
2.8067	Hydrogen Bond	A:GLY1185:O	:9:H11
2.7716	Halogen	A:TYR1203:O	:9:F
3.5167	Halogen	A:TYR1203:O	:9:F
3.2179	Halogen	A:GLY1205:C	:9:F
4.2409	Other	A:TYR1213	:9:S
4.5193	Hydrophobic	A:TYR1213	:9
3.6960	Hydrophobic	A:TYR1224	:9
4.4352	Hydrophobic	A:TYR1224	:9
4.2476	Hydrophobic	A:GLY1205:C,O;GLY1206:N	:9
5.1987	Hydrophobic	A:ILE1228	:9
5.0514	Hydrophobic	A:ALA1215	:9
**Distance Å**	**Bonding**	**Binding site of target (DLD-1)**	**Binding site of ligand (10)**
2.1472	Hydrogen Bond	A:TYR1203:HN	:10:O
2.8026	Hydrogen Bond	A:TYR1213:HN	:10:O
2.1841	Hydrogen Bond	A:GLY1185:O	:10:H
2.4503	Hydrogen Bond	A:HIS1184:HE1	:10:O
2.3535	Hydrogen Bond	A:SER1186:HB2	:10:N
2.5023	Hydrogen Bond	A:ALA1202:HA	:10:O
2.7082	Hydrogen Bond	A:GLU1291:OE2	:10:H12
3.1131	Halogen	A:HIS1184:NE2	:10:F
4.3586	Hydrophobic	A:TYR1213	:10
4.2263	Hydrophobic	A:TYR1224	:10
5.3642	Hydrophobic	A:TYR1224	:10
3.9482	Hydrophobic	A:ILE1212	:10:C
4.8705	Hydrophobic	A:HIS1184	:10:C
5.2361	Hydrophobic	A:PHE1197	:10:C
4.9276	Hydrophobic	A:ILE1228	:10
4.1287	Hydrophobic	A:ALA1202	:10
4.8497	Hydrophobic	A:ILE1212	:10
**Distance Å**	**Bonding**	**Binding site of target (DLD-1)**	**Binding site of ligand (14)**
1.9310	Hydrogen Bond	A:ASP1198:HN	:14:N
1.8816	Hydrogen Bond	A:TYR1213:HN	:14:N
1.9719	Hydrogen Bond	A:HIS1184:HE1	:14:N
2.7246	Hydrogen Bond	A:SER1186:HB2	:14:O
3.0242	Hydrogen Bond	A:SER1186:HB2	:14:O
2.7318	Hydrogen Bond;Halogen	A:PHE1214:HA	:14:F
2.5995	Hydrogen Bond	A:LYS1220:HE2	:14:F
2.4053	Hydrogen Bond	A:ASP1198:O	:14:H12
2.3739	Hydrogen Bond	A:TYR1203:O	:14:H15
3.3136	Halogen	A:TYR1213:C	:14:F
2.9833	Halogen	A:TYR1213:O	:14:F
3.1391	Halogen	A:PHE1214:C	:14:F

2.6669	Halogen	A:PHE1214:O	:14:F
3.4727	Halogen	A:GLU1291:OE2	:14:F
5.1137	Hydrophobic	A:TYR1213	:14
3.6852	Hydrophobic	A:TYR1224	:14
4.6748	Hydrophobic	A:TYR1224	:14:C
4.6463	Hydrophobic	A:ALA1202	:14
5.2916	Hydrophobic	A:ILE1192	:14
4.8413	Hydrophobic	A:ILE1212	:14
